# Supplementary material for: Epidemiological, serological, and viral genomic analysis of an outbreak of elephant hemorrhagic disease in Switzerland
Source: PLoS One. 2025 Apr 7;20(4):e0301247. doi: 10.1371/journal.pone.0301247 (PMC11975098; doi:10.1371/journal.pone.0301247)
Supplement: S1 File — (DOCX) [file pone.0301247.s001.docx]

Supplementary information: comparison of EEHV genomes

Recent partial and full genomic DNA sequence analysis of many distinct EEHV species and subtypes have revealed large genetic differences between them (4, 10, 17-19). Furthermore, even within strains of the EEHV1 species itself there are at least 28 viral-encoded proteins that fall into multiple highly-diverged subtype clusters, including most of the likely envelope glycoproteins such as gB, gH, gL and the three adjacent spliced glycoproteins ORF-O, ORF-P and ORF-Q that are expected to play major roles in immunity and serology.

The first extensive descriptions of genetic divergence between prototype EEHV1B (Emelia) strain compared to those of the prototype EEHV1A (Kimba and Raman) strains were given in Wilkie et al (17), Ling et al (5)and Richman et al (4). These reports revealed several non-adjacent multigene protein-encoding segments that exhibited often extraordinarily large genetic differences. In particular, Richman et al (4) identified three major diverged multi-gene chimeric domains referred to as CD-I (gB/POL), CD-II (ORF-J/gN/gO/gH/TK) and CD-III (UDG/gL/ORF-O/ORF-P/ORF-Q) that map within the central portions of the genome and together encompass part or all of 12 viral genes. These together occupy nearly 15 kb with all but the more conserved POL locus here differing by between 21% and 37% at the protein level between the EEHV1A and EEHV1B versions (4, 5). Despite being widely dispersed these three loci are fully linked and nearly identical in all eight known examples of the EEHV1B diaspora that have been fully evaluated, including EEHV1B (Emelia) and EEHV1B (Kiba) (16) as the prototypes.

Both simplified cartoon genetic maps dividing the prototype EEHV1A (Kimba) genome into L1/L2/L3/C1/C2/R1 and R2 gene-blocks (28) as well as a detailed to-scale gene map of Kimba (5) have been published previously. Overall, comparative analyses at both the single gene or localized sub-genomic level by PCR sequencing, as well as in some cases at the complete genome level, have revealed multiple patterns of genetic diversity not only between different AT-rich and GC-rich branch EEHV species, but also among individual isolates (i.e. strains and subtypes) of each EEHV species (4, 5, 10, 19).

Later, when numerous additional strains of EEHV1A were examined in greater detail the CD-II and CD-III regions especially proved instead to display multiple tight cladal clusters of not just subtype A but also many more variants (designated as subtypes C, D, E, F and G etc.) within individual proteins such as U48 (gH) and U82 (gL) as well as E37 (ORF-O), E38 (ORF-P) and E39 (ORF-Q) for example. Moreover, there are also several other subsequently identified hypervariable loci with multiple subtypes mapping across other parts of both the EEHV1A and EEHV1B genomes that are neither linked to each other nor to the classical A versus B diaspora. These encode proteins such as E3(vGPCR6), E5 (vGPCR5), E23/E24/E25 (vOX2-2/3/4) = (CD-VII), E30/31(= CD-VI) and U51(vGPCR1 (= CD-VIII), as well as up to six more hypervariable genes within the R2-segment that also either fall into multiple highly-diverged clusters, or sometimes include being fragmented or having otherwise inactivated versions. Finally, there are also eight alternative groups of inserted triplex R2-segment gene ‘cassettes’ that have different gene contents and insertion points (see below).

Some details about individual gene subtyping among multiple strains have been presented previously as phylogenetic trees and/or as polymorphism charts. For example, for U39 (gB) and U38 (POL) in Richman et al (4) for U51(vGPCR1) in Long et al (1) for E5 (vGPCR5) and E54 (vOX2-1) in Oo et al (29), in U48.5 (TK) and U48 (gH) (= CD-II) in Zachariah et al (28) and also for E39 (ORF-Q) (= CD-III) in Fuery et al (7). On the other hand, details about the levels of variability and subtype clustering for several other loci e.g. CD-VI (E30/31), CD-VII (E23/24/25) as well as for E37 (ORF-O) and E38 (ORF-P) and especially about the multiple alternative triplex gene ‘cassettes’ within the R2-segment of EEHV1 strains have not been described previously.

Our analysis of these new cases included a full evaluation of the single novel EEHV1 (Umesh, EP55) strain involved and its patterns of divergence at both the intact genomic and single protein-coding gene levels from other previously encountered or published EEHV1 reference strains.
