# Supplementary material for: Epidemiological, serological, and viral genomic analysis of an outbreak of elephant hemorrhagic disease in Switzerland
Source: PLoS One. 2025 Apr 7;20(4):e0301247. doi: 10.1371/journal.pone.0301247 (PMC11975098; doi:10.1371/journal.pone.0301247)
Supplement: S1 Table — (DOCX) [file pone.0301247.s002.docx]

**Table S1**. EEHV1A (Umesh) Variable Gene Subtype Comparisons.

**Section I: Main Genome, Protein Level.**

| **Gene/CaseName** | **Kimba** | **Raman** | **Emelia** | **Pathiri** | **Kala** | **Xian** | **Ganesh** | **Singgah** | **Preya** | **Umesh** |
| --- | --- | --- | --- | --- | --- | --- | --- | --- | --- | --- |
| E3/vGPCR6 | A1 | B3 | B2 | A1 | B1 | B1 | C2 | B1 | B1 | A1 |
| E5/vGPCR5 | A | A | C | B | A | B | A | A | A | C |
| E23/vOX2-4 | A | A | A | A | A | A | A | A | A | A |
| E24/vOX2-3 | A | A | A | A | A | A | A | A | A | A |
| E25/vOX2-2 | A | A | A | A | A | A | A | C | A | A |
| E26/vGPCR3 | A | A | A | A | A | B | B | A | A | B |
| E30ex1/2 | A1 | A1 | B | ? | C1 | C2 | ? | F | D | C1 |
| E31ex1/2 | A | A | B | A | C | C | ? | F | D | C |
| U39/gB(CD-I) | A1 | A2 | B | ? | A1 | C2 | A1 | C3 | C2 | C1 |
| E35A/ORF-J(CD-II) | C | C | B | A | A | A | C | C | A | A |
| U46/gN(CD-II) | C | C/A | B | A | A | A | C | C | A | C |
| U47/gO(CD-II) | C | A | B | A | C | A | C | C | A | A |
| U48/gH(CD-II) | A2 | C1 | B | E | A1 | E | A2 | F | C2 | D |
| U48.5/TK(CD-II) | A | A | B | A | A | A | A | A | C | A |
| U51(vGPCR1) | A | D2 | B | E/A | A1 | E/A | D1/A | D2 | A2 | E |
| U81/UDG(CD-III) | A | A | B | A | A | A | C | A | A | A |
| U82/gL(CD-III) | A1 | A2 | B | D | A1 | C | D | C | C | A1 |
| E37/ORF-O(CD-III) | A1 | C | B | A2 | C | E | A2 | G | F | C |
| E38/ORF-P(CD-III) | A | E | B | F | D | G | F | I | H2 | E |
| E39/ORF-Q(CD-III) | A | C | B | A | A | D1 | A | F | E1 | C |

**Section II: R2 Segment, Protein Level.**

| **Gene/CaseName** | **Kimba** | **Raman** | **Emelia** | **Pathiri** | **Kala** | **Xian** | **Ganesh** | **Singgah** | **Preya** | **Umesh** |
| --- | --- | --- | --- | --- | --- | --- | --- | --- | --- | --- |
| E47/vFUT9 | A | A | B | B | A | B | B | B | B | B |
| #E48/vGPCR7 | A | A | - | - | - | - | - | - | - | - |
| #E49 | + | + | - | - | - | - | - | - | - | - |
| #E50/vIgFam1 | + | + | - | - | - | - | - | - | - | - |
| E50.5/vGPCR8/9 | fr | fr | B1 | C | del | C | C | B1 | B3 | C |
| E51 | A1 | A1 | B1 | D1 | H | D1 | D1 | B2 | B3 | D1fs |
| E52/vIgFam2 | A1 | A1 | fr | fr | A2 | fr | fr | del | fr | del |
| #E56/vGPCR12 | - | - | - | - | F | - | - | - | - | - |
| #E57/vIgFam4 | - | - | - | - | + | - | - | - | - | - |
| #E58/vIgFam5 | - | - | - | - | + | - | - | - | - | - |
| #E59/vGPCR10) | - | - | - | - | - | D | D | - | - | D |
| #E60(vIgFam6) | - | - | - | - | - | + | + | - | - | + |
| #E61(vIgFam7) | - | - | - | - | - | + | + | - | - | fs |
| #E68 | - | - | B1 | B3 | - | - | - | B3 | B4 | - |
| #E69/vIgFam12 | - | - | + | del | - | - | - | del | del | - |
| #E70/vIgFam13 | - | - | + | + | - | - | - | + | + | - |
| E52.5/vIgFam2.3 | A | D | B | del | E | B | del | C | del | del |
| E53/(vIgFam2.5 | fs | + | + | fs | + | + | + | + | fs | + |
| E54/vOX2-1 | o | o | o | o | o | o | o | o | o | o |
| E55/vIgFam3 | A1 | B1 | B1 | A2 | D | B5 | B3 | C | A1 | A2 |

**Section III: Conserved Core Genes, Low DNA Level Variation Only.**

| **Gene/CaseName** | **Kimba** | **Raman** | **Emelia** | **Pathiri** | **Kala** | **Xian** | **Ganesh** | **Singgah** | **Preya** | **Umesh** |
| --- | --- | --- | --- | --- | --- | --- | --- | --- | --- | --- |
| U38/POL(CD-I) | A | A | B | A | A | A | A | A | A | A |
| U60TERex3 | A | C | B | AB | A | A | A | A | A | A |
| U71/gM | A | A | B | A | A | A | A | C | A | A |
| U73/OBP | A | A | B | A | A | A | A | A | C | A |
| U77/HEL | A | A | B | C | A | C | A | A | A | C |

**Footnotes to Table S1.** Listing of all the hypervariable protein cluster subtypes designated for each EEHV1 strain are shown in comparison with the EEHV1A (Umesh) subtype designations. Section I, Main genome comparing the 22 most hypervariable protein encoding loci. Section II, R2-segment, 8 variable protein-encoding genes plus four alternative triple gene cassettes; Section III, Conserved core gene subtypes with low DNA level variability only.

+ = Inserted alternative gene cassettes that do not display subtyping.

- = Absence of any inserted gene cassettes at these loci.

Del, fr or fs = deleted, fragmented or frame-shifted protein versions.

O = Intact E54 proteins which display up to 15% variability at the aa level, but without any clustal subtyping patterns.

* E37(ORF-O), E38 (ORF-P) and E39 (ORF-Q) proteins are also part of the CD-III chimeric domain.

# Just four of the eight known alternative inserted three-gene cassette groups (A, B, D and F) in the R2-segment were selected for inclusion here (from a total of 38 EEHV1 strains examined).
