# Supplementary material for: Epidemiological, serological, and viral genomic analysis of an outbreak of elephant hemorrhagic disease in Switzerland
Source: PLoS One. 2025 Apr 7;20(4):e0301247. doi: 10.1371/journal.pone.0301247 (PMC11975098; doi:10.1371/journal.pone.0301247)
Supplement: S2Table — (DOCX) [file pone.0301247.s003.docx]

**Table S2. Details about the Ten EEHV1 Genomes Used in Table S1**

| Virus Strain | Code | Year | Age | Sex | Country | Complete Genome | gB/POL  (CD-I) | vGPCR1 | E23/25 | gH/TK  (CD-II) | ORF-OPQ  (CD-III) | R2 segment (E47-E55) |
| --- | --- | --- | --- | --- | --- | --- | --- | --- | --- | --- | --- | --- |
| EEHV1A(Kimba) | NAP23 | 2004 | 13Y | F | USA | KC618527 |  |  |  |  |  |  |
| EEHV1A(Raman) | EP22 | 2009 | 32m | M | UK | KC462165 |  |  |  |  |  |  |
| EEHV1B(Emelia) | EP18 | 2006 | 33m | F | UK | KC462164 |  |  |  |  |  |  |
| EEHV1A(Pathiri) | IP11 | 2008 | ? | F | India |  | JX011036 | JX011051 | MG958690 | KT832531 | MH287547 | MH287516 |
| EEHV1A(Kala) | NAP18 | 2000 | 30m | M | USA |  | HM568523 | GU350758 | MG958682 | HM56825 | JX011080 | MH287526 |
| EEHV1A(Xian) | EP07 | 1999 | 28m | M | Swi |  | JF692769 | KT705233 | MG958687 | KM458135 | MH287543 | MH287538 |
| EEHV1A(Ganesh) | NAP26 | 2005 | 7Y | M | USA |  | JF692756 | KT705227 | MG958667 | KM458122 | MH287547 | MH287521 |
| EEHV1A(Singgah) | NAP17 | 2000 | 6Y | F | USA |  | JF692750 | KT705221 | MG958677 | KM458117 | MH287560 | MH287537 |
| EEHV1A(Preya) | NAP21 | 2003 | 38m | F | USA |  | JF692752 | KT705223 | MG958686 | KM458118 | MH287559 | MH287535 |
| EEHV1A(Umesh) | EP55 | 2022 | 28m | M | Swi | OR543011 |  |  |  |  |  |  |

**Footnote to Table S2.** Additional information about the ten selected EEHV1 strains and Asian elephant hosts included in Table S1. All had been diagnosed with lethal EHD and viral genomic DNA sequence data was obtained by either selective PCR locus DNA gene sequencing or by whole genome Illumina DNA sequencing from post-mortem blood or necropsy tissue samples. Swi = Switzerland. GenBank accession numbers are given for either whole genome files or for representative selected sub-genomic loci including U39/38(gB/POL}, U51(vGPCR1), E23/24/25(vOX2-2/3/4), U48/48.5(gH/TK), E37/38/39(ORF-O/P/Q) and the R2-segment (E47-E55).
