# Supplementary material for: Epidemiological, serological, and viral genomic analysis of an outbreak of elephant hemorrhagic disease in Switzerland
Source: PLoS One. 2025 Apr 7;20(4):e0301247. doi: 10.1371/journal.pone.0301247 (PMC11975098; doi:10.1371/journal.pone.0301247)
Supplement: Attachment — Submitted filename: PONE-D-24-09462_R1 - review 2.pdf [file pone.0301247.s006.pdf]

PLOS ONE

Epidemiological, Serological, and Viral Genomic Analysis of an Outbreak of Elephant Hemorrhagic Disease in Switzerland

--Manuscript Draft--

**Manuscript Number:**	PONE-D-24-09462R1
**Article Type:**	Research Article
**Full Title:**	Epidemiological, Serological, and Viral Genomic Analysis of an Outbreak of Elephant Hemorrhagic Disease in Switzerland
**Short Title:**	Epidemiological, serological, and viral genomic analysis of EHD in Switzerland
**Corresponding Author:**	Mathias Ackermann<br>University of Zurich Institute of Virology: Universitat Zurich Virologisches Institut<br>Zurich, SWITZERLAND
**Keywords:**	Elephant hemorrhagic disease (EHD); Elephant endotheliotropic herpesviruses (EEHV); Epidemiology; Serology; genomic sequences, strain variation and subtyping
**Abstract:**	Elephant hemorrhagic disease (EHD), caused by several Elephant endotheliotropic herpesviruses (EEHV), represents a frequently lethal syndrome, affecting both captive and free-living elephants. In the summer of 2022, three young Asian elephants (Elephas maximus) succumbed to EHD in a zoo in Switzerland, despite of considerable preventive efforts and early detection of EEHV1A viremia. In this communication, we describe the extent of preventive measures in terms of prior virus detection, active survey of viremia, and antibody status. The results show that: (1) A previously undetected EEHV1A strain had remained unrecognized among these elephants. Probably, the virus re-emerged after almost 40 years of latency from one of the oldest elephants in the zoo. (2) While two of the three affected animals had prior immune responses against EEHV1, their strain-specific immunity proved insufficient to prevent EHD. ~~We have also determined~~ the complete genomic DNA sequence of the EEHV1A strain involved and provide detailed comparisons with the numerous chimeric and hypervariable subtypes of individual genes from multiple other EEHV1 strains revealing a much greater extent of divergence and level of complexity among the encoded proteins than previously described.
**Order of Authors:**	Mathias Ackermann
	Jakub Kubacki
	Sarah Heaggans
	Gary S. Hayward
	Julia Lechmann
**Response to Reviewers:**	All queries have been addressed and the responses are included in table format included with the resubmission
**Additional Information:**	
**Question**	**Response**
**Financial Disclosure**<br><br>Enter a financial disclosure statement that describes the sources of funding for the work included in this submission. Review the submission guidelines for detailed requirements. View published research articles from *PLOS ONE* for specific examples.	Yes

Powered by Editorial Manager® and ProduXion Manager® from Aries Systems Corporation

This statement is required for submission and **will appear in the published article** if the submission is accepted. Please make sure it is accurate.<br><br>**Funded studies**<br>Enter a statement with the following details:<br>• Initials of the authors who received each award<br>• Grant numbers awarded to each author<br>• The full name of each funder<br>• URL of each funder website<br>• Did the sponsors or funders play any role in the study design, data collection and analysis, decision to publish, or preparation of the manuscript?<br><br>Did you receive funding for this work?	
Please add funding details.<br>as follow-up to "**Financial Disclosure**<br><br>Enter a financial disclosure statement that describes the sources of funding for the work included in this submission. Review the submission guidelines for detailed requirements. View published research articles from *PLOS ONE* for specific examples.<br><br>This statement is required for submission and **will appear in the published article** if the submission is accepted. Please make sure it is accurate.<br><br>**Funded studies**<br>Enter a statement with the following details:<br>• Initials of the authors who received each award<br>• Grant numbers awarded to each author<br>• The full name of each funder<br>• URL of each funder website<br>• Did the sponsors or funders play any role in the study design, data collection and analysis, decision to publish, or preparation of the manuscript?<br><br>Did you receive funding for this work?"	This work was supported by a private donation of the late Prof. Dr. Robert Wyler to M.A. (D-52601-01-01) as well as by general funds allocated to J.L. by the Institute of Virology, University of Zurich.<br>The funders had no role in study design, data collection and analysis, decision to publish, or preparation of the manuscript.

Please select the country of your main research funder (please select carefully as in some cases this is used in fee calculation).<br>as follow-up to "**Financial Disclosure**<br><br>Enter a financial disclosure statement that describes the sources of funding for the work included in this submission. Review the submission guidelines for detailed requirements. View published research articles from *PLOS ONE* for specific examples.<br><br>This statement is required for submission and **will appear in the published article** if the submission is accepted. Please make sure it is accurate.<br><br>**Funded studies**<br>Enter a statement with the following details:<br>• Initials of the authors who received each award<br>• Grant numbers awarded to each author<br>• The full name of each funder<br>• URL of each funder website<br>• Did the sponsors or funders play any role in the study design, data collection and analysis, decision to publish, or preparation of the manuscript?<br><br>Did you receive funding for this work?"	SWITZERLAND - CH
**Competing Interests**<br><br>Use the instructions below to enter a competing interest statement for this submission. On behalf of all authors, disclose any competing interests that could be perceived to bias this work—acknowledging all financial support and any other relevant financial or non-financial competing interests.<br><br>This statement is required for submission and **will appear in the published article** if the submission is accepted. Please make sure it is accurate and that any funding	The authors have declared that no competing interests exist.

Powered by Editorial Manager® and ProduXion Manager® from Aries Systems Corporation

<table>
<tr><td>sources listed in your Funding Information later in the submission form are also declared in your Financial Disclosure statement.

View published research articles from PLOS ONE for specific examples.

NO authors have competing interests

Enter: The authors have declared that no competing interests exist.

Authors with competing interests

Enter competing interest details beginning with this statement:

I have read the journal's policy and the authors of this manuscript have the following competing interests: [insert competing interests here]

* typeset</td><td></td></tr>
<tr><td>Ethics Statement

Enter an ethics statement for this submission. This statement is required if the study involved:

• Human participants
• Human specimens or tissue
• Vertebrate animals or cephalopods
• Vertebrate embryos or tissues
• Field research

Write "N/A" if the submission does not require an ethics statement.

General guidance is provided below. Consult the submission guidelines for detailed instructions. Make sure that all information entered here is included in the Methods section of the manuscript.</td><td>Viremia samples, tissue samples and sera from elephants were analyzed by order of the contributing zoos. Ethical approval was not sought as the present study only made use of samples that were taken for specific diagnostic purposes, the results of which are described in this manuscript. The samples were taken under routine veterinary care by veterinary staff. The contributing zoos as well as their scientific advisors consider it important to sharing critical medical, husbandry, training, and scientific information with colleagues across the globe.</td></tr>
</table>

**Format for specific study types**<br><br>**Human Subject Research (involving human participants and/or tissue)**<br>• Give the name of the institutional review board or ethics committee that approved the study<br>• Include the approval number and/or a statement indicating approval of this research<br>• Indicate the form of consent obtained (written/oral) or the reason that consent was not obtained (e.g. the data were analyzed anonymously)<br><br>**Animal Research (involving vertebrate animals, embryos or tissues)**<br>• Provide the name of the Institutional Animal Care and Use Committee (IACUC) or other relevant ethics board that reviewed the study protocol, and indicate whether they approved this research or granted a formal waiver of ethical approval<br>• Include an approval number if one was obtained<br>• If the study involved *non-human primates*, add *additional details* about animal welfare and steps taken to ameliorate suffering<br>• If anesthesia, euthanasia, or any kind of animal sacrifice is part of the study, include briefly which substances and/or methods were applied<br><br>**Field Research**<br><br>Include the following details if this study involves the collection of plant, animal, or other materials from a natural setting:<br>• Field permit number<br>• Name of the institution or relevant body that granted permission	
**Data Availability**<br><br>Authors are required to make all data underlying the findings described fully available, without restriction, and from the time of publication. PLOS allows rare exceptions to address legal and ethical concerns. See the PLOS Data Policy and FAQ for detailed information.	Yes - all data are fully available without restriction

A Data Availability Statement describing where the data can be found is required at submission. Your answers to this question constitute the Data Availability Statement and **will be published in the article**, if accepted.<br><br>**Important:** Stating 'data available on request from the author' is not sufficient. If your data are only available upon request, select 'No' for the first question and explain your exceptional situation in the text box.<br><br>Do the authors confirm that all data underlying the findings described in their manuscript are fully available without restriction?	
**Describe where the data may be found in full sentences. If you are copying our sample text, replace any instances of XXX with the appropriate details.**<br><br>• If the data are **held or will be held in a public repository**, include URLs, accession numbers or DOIs. If this information will only be available after acceptance, indicate this by ticking the box below. For example: *All XXX files are available from the XXX database (accession number(s) XXX, XXX.*).<br>• If the data are all contained **within the manuscript and/or Supporting Information files**, enter the following: *All relevant data are within the manuscript and its Supporting Information files.*<br>• If neither of these applies but you are able to provide **details of access elsewhere**, with or without limitations, please do so. For example:<br><br>*Data cannot be shared publicly because of [XXX]. Data are available from the XXX Institutional Data Access / Ethics Committee (contact via XXX) for researchers who meet the criteria for access to confidential data.*<br><br>*The data underlying the results presented in the study are available from (include the name of the third party*	All raw sequencing data generated were uploaded to the Sequence Read Archive (SRA) as BioProject ID: PRJNA1052582<br>The full sequence of EP55 has been submitted to GenBank: GenBank accession number OR543011

and contact information or URL).<br>• This text is appropriate if the data are owned by a third party and authors do not have permission to share the data.<br><br>* typeset	
Additional data availability information:	

Powered by Editorial Manager® and ProduXion Manager® from Aries Systems Corporation

Revised Manuscript
Click here to access/download;Manuscript;Revised manuscript.docx

Epidemiological, Serological, and Viral Genomic Analysis of an Outbreak of Elephant Hemorrhagic Disease in Switzerland

Mathias Ackermann[1*], Jakub Kubacki[1,2,4], Sarah Heaggans[3], Gary S. Hayward[3], Julia Lechmann[1]

[1]Institute of Virology, Vetsuisse Faculty, University of Zurich, 8057 Zurich, Switzerland

[2] Institute of Virology and Immunology, Mittelhäusern, Switzerland

[3] Viral Oncology Program, Johns Hopkins School of Medicine, Baltimore, Maryland, USA

[4] Department of Infectious Diseases and Pathobiology (DIP), Vetsuisse Faculty, University of Bern, Bern, Switzerland.

*Corresponding author

E-mail: mathias.ackermann@uzh.ch

Short Title: Epidemiological, serological, and viral genomic analysis of EHD in Switzerland

Key words: Elephant hemorrhagic disease (EHD); Elephant endotheliotropic herpesviruses (EEHV); Epidemiology; Serology; genomic sequences, strain variation and subtyping

Abstract

Elephant hemorrhagic disease (EHD), caused by several Elephant endotheliotropic herpesviruses (EEHV), represents a frequently lethal syndrome, affecting both captive and free-living elephants. In the summer of 2022, three young Asian elephants (*Elephas maximus*) succumbed to EHD in a zoo in Switzerland, despite of considerable preventive efforts and early detection of EEHV1A viremia. In this communication, we describe the extent of preventive measures in terms of prior virus detection, active survey of viremia, and antibody status. The results show that: (1) A previously undetected EEHV1A strain had remained unrecognized among these elephants. Probably, the virus re-emerged after almost 40 years of latency from one of the oldest elephants in the zoo. (2) While two of the three affected animals had prior immune responses against EEHV1, their strain-specific immunity proved insufficient to prevent EHD. ~~We have also determined~~ the complete genomic DNA sequence of the EEHV1A strain involved and provide detailed comparisons with the numerous chimeric and hypervariable subtypes of individual genes from multiple other EEHV1 strains revealing a much greater extent of divergence and level of complexity among the encoded proteins than previously described.

Introduction

Elephant hemorrhagic disease (EHD) can be caused by several Elephant endotheliotropic herpesviruses (EEHV, members of the genus *Proboscivirus* within the family of *Orthoherpesviridae*) and represents a frequently lethal syndrome, affecting both captive and free-living elephants (1-4). Seven distinct but genetically related ~~types (~~species~~)~~ of EEHV that fall into both an AT-rich and GC-rich branch are known to infect either Asian (*Elephas maximus*) or African elephants (*Loxodonta africana*) (5, 6). EEHV type 1 (EEHV1), which has been most frequently associated with the occurrence of EHD in both captive and Asian elephants, can be further divided into the chimeric subtypes A (EEHV1A) and B (EEHV1B), as well as into cladal ORF-Q protein subtype clusters encoded by gene E39 (4, 6, 7). Reagents

capable of detecting ORF-Q clades A, B, C or D have been generated and used successfully at both the genetic as well as on the serological level (7), although the existence of at least three further ORF-Q subtypes (clades E1, E2, and F) has now been documented by GenBank entries (E1: QBP78682; E2: QOE74866.1 and QOE74629; F: QBP78717).

The genetic counterpart of EEHV1 in African elephants has been named EEHV6 (8). Among others, EEHV6 has been linked with several cases of EHD in African elephants in both Europe and the USA (1). The other five EEHV species are represented by EEHV2, EEHV3 and EEHV7 in African elephants (4, 9, 10) plus EEHV4 and EEHV5 in Asian elephants (1, 6, 10).

The study of epidemiology, pathogenesis, treatment, and prevention of EEHV-related diseases has been hindered because, as yet, none of the EEHV species or strains can be propagated in conventional cell cultures. Moreover, animal models for EEHV and EHD do not exist because no other susceptible species have been identified. Recent partial and full genomic DNA sequence analysis of many EEHV species and strains have revealed large genetic differences between them (4, 9, 10, 11, 18). Furthermore, even within strains of the EEHV1 species itself there are at least 28 viral-encoded proteins that fall into multiple highly-diverged subtype clusters, including most of the likely envelope glycoproteins such as gB, gH, gL and the three adjacent spliced glycoproteins ORF-O, ORF-P and ORF-Q that are expected to play major roles in immunity and serology.

As with all herpesviruses, the reservoir of EEHV is in latently infected animals, which may intermittently reactivate and shed virus for transmission to herdmates (1, 11). The pathogenesis of EHD is poorly understood. While all known EEHV species replicate in the respiratory tract for virus excretion and transmission, viremia may also occur intermittently, which is the major precondition for developing ~~symptomatic~~ EHD (12). Another critical parameter for developing EHD is the level of specific anti-EEHV antibodies in the serum. Very young animals are thought to be protected by maternal antibodies. During this period,

subclinical infections may take place, which induces active immunological protection against the disease later in life (7, 13, 14). Accordingly, the highest risk for young animals to succumb to EHD is during and right after weaning (2-8y), in association with the waning of maternal antibodies.

Viral genomic sequences have been very useful to determine transmission chains among elephants, which eventually lead to interconnected cases of EHD. For example, the PCR DNA sequences were shown to be identical over at least 3000 bp encompassing the E38/POL, U48/gH-TK), U51/vGPCR1 and U71/gM loci between three cases of EHD at the Berlin Zoo that occurred 11 years apart: Plai Kiri (European *Proboscivirus* case number 14; EP14) in the year 2000, Shaina Pali (EP23) and Ko-Raya (EP25) in 2011 (e.g. GenBank KT705234.1, KT705238.1 and KT705239.1). The knowledge of the current viral sequences is also important for prognosis and treatment: subtype 1A is considered the most virulent EEHV affecting Asian elephants, whereas subtype 1B is known to frequently cause milder courses of EHD (1, 15). Only recently, the value of serology has also been established as a useful tool in the context of EEHV epidemiology as well as in EHD risk management (2, 7, 13, 14).

Albeit highly diverged, all known species of EEHV encode thymidine kinase (Tk) as well as conserved protein kinase enzymes (CPK), i.e. U48.5/EE7 encoding the EEHV version of Tk (ETk) and U69 encoding the EEHV version of CPK (ECPK), respectively. It has been speculated that these viral enzymes may be capable of phosphorylating nucleoside analogues such as ganciclovir (GCV), aciclovir (ACV), and penciclovir (PCV) or famciclovir (FCV, the PCV-derivate for oral application), which is an essential prerequisite for ~~their potential antiviral activity against EEHV~~ (16-20). Indeed, nucleoside analogues such as ACV, FCV, and GCV have been used to treat elephants with clinical and virological EHD, but the clinical efficacy of these drugs remains debatable (1, 21-23), although intravenous treatment with GCV coincided at least in one case with decreasing viremia (24). However, in the absence of

animal models and suitable cell cultures, the true value of treatment against EEHV-infections with nucleoside analogues remains unknown (1).

Despite all these uncertainties, the desire to prevent lethal cases of EHD among young elephants in captivity increased with the growing knowledge about its causative agents and their biological properties. Accordingly, many zoos initiated surveillance programs to identify prevalent EEHV types and strains, and to monitor their animals at risk for early signs of viremia (12, 25). It was expected that typing of the prevalent EEHV species and strains may help to focus on particularly dangerous isolates, while screening for viremia may allow early onset of antiviral treatment in cases of emergency (15, 21, 24, 26).

Prior to 2022, three cases of lethal EHD had been recorded among Asian elephants in Switzerland, all of them attributable to EEHV1A, the first in 1988 (partial sequences deposited in Genbank as EP04, Lohimi: KT705308.1), the second in 1999 (partial sequences in GenBank as EP07, Xian: MH287538.1) and the third in 2003 (EP16, Aishu; no sequence entries in GenBank but unpublished U48.5/EE7 sequences, encoding for ETk, classified the isolate as subtype 1A) (25, 27). In 2014, after many years of successful breeding in a closed collection, a 10-year-old male (Thai, see Materials and Methods) was introduced to the herd, with the aim to replace the aging prior breeding bull (Maxi) and to avoid inbreeding. As a calf had been born in the same year (Omysha), a protocol was established, which included risk assessment and active surveillance. Initially, EEHV-shedding was assessed, which resulted in the detection of two additional circulating viruses among these elephants, namely an EEHV1B isolate from Omysha as well as an EEHV4 isolate from Thai (25). In 2017 and 2020, respectively, two additional elephant calves were born in the zoo, both of which had been fathered by the newly acquired breeding bull. Starting at the age of one year, the young elephants were considered as being at risk to succumbing to EHD. Accordingly, they were included into the preventive program and screened on a weekly basis for viremia by EEHV1 and EEHV4 (26). Serology was established "*en route*" ~~as it became available from literature~~

(2, 7, 13). Moreover, stocks of GCV and FCV were acquired for treatment of clinical EHD cases.

In summer 2022, the three juvenile elephants from this herd died of EHD caused by a previously undetected EEHV1A strain. This paper describes our attempts at identification of the source of this outbreak through genomic DNA sequencing of the causative virus as well as to evaluate its relationship to other well-characterized EEHV1 strains and for identifying encoded antigenic proteins relevant for developing specific serological assays.

Recent partial and full genomic DNA sequence analysis of many distinct EEHV species and subtypes have revealed large genetic differences between them (4, 10, 17-19). Furthermore, even within strains of the EEHV1 species itself there are at least 28 viral-encoded proteins that fall into multiple highly-diverged subtype clusters, including most of the likely envelope glycoproteins such as gB, gH, gL and the three adjacent spliced glycoproteins ORF-O, ORF-P and ORF-Q that are expected to play major roles in immunity and serology.

The first extensive descriptions of genetic divergence between prototype EEHV1B (Emelia) strain compared to those of the prototype EEHV1A (Kimba and Raman) strains were given in Wilkie et al (10), Ling et al (11) and Richman et al (4). These reports revealed several non-adjacent multigene protein-encoding segments that exhibited often extraordinarily large genetic differences. In particular, Richman et al (4) identified three major diverged multi-gene chimeric domains referred to as CD-I (gB/POL), CD-II (ORF-J/gN/gO/gH/TK) and CD-III (UDG/gL/ORF-O/ORF-P/ORF-Q that map within the central portions of the genome and together encompass part or all of 12 viral genes. These together occupy nearly 15 kb with all but the more conserved POL locus here differing by between 21% and 37% at the protein level between the EEHV1A and EEHV1B versions (4, 5). Despite being widely dispersed these three loci are fully linked and nearly identical in all eight known examples of the EEHV1B diaspora that have been fully evaluated, including EEHV1B (Emelia) and EEHV1B (Kiba) (16) as the prototypes.

Both simplified cartoon genetic maps dividing the prototype EEHV1A (Kimba) genome into L1/L2/L3/C1/C2/R1 and R2 gene-blocks (28) as well as a detailed to-scale gene map of Kimba (5) have been published previously. Overall, comparative analyses at both the single gene or localized sub-genomic level by PCR sequencing, as well as in some cases at the complete genome level have revealed multiple patterns of genetic diversity not only between different AT-rich and GC-rich branch EEHV species, but also among individual isolates (i.e. strains and subtypes) of each EEHV species (4, 5, 10, 19).

Later, when numerous additional strains of EEHV1A were examined in greater detail the CD-II and CD-III regions especially proved instead to display multiple tight cladal clusters of not just subtype A but also many more variants (designated as subtypes C, D, E, F and G etc.) within individual proteins such as U48 (gH) and U82 (gL) as well as E37 (ORF-O), E38 (ORF-P) and E39 (ORF-Q) for example. Moreover, there are also several other subsequently identified hypervariable loci with multiple subtypes mapping across other parts of both the EEHV1A and EEHV1B genomes that are neither linked to each other nor to the classical A versus B diaspora. These encode proteins such as E3(vGPCR6), E5 (vGPCR5), E23/E24/E25 (vOX2-2/3/4) = (CD-VII), E30/31(= CD-VI) and U51(vGPCR1 (= CD-VIII), as well as up to six more hypervariable genes within the R2-segment that also either fall into multiple highly-diverged clusters, or sometimes include being fragmented or having otherwise inactivated versions. Finally, there are also eight alternative groups of inserted triplex R2-segment gene 'cassettes' that have different gene contents and insertion points (see below).

Some details about individual gene subtyping among multiple strains have been presented previously as phylogenetic trees and/or as polymorphism charts. For example, for U39 (gB) and U38 (POL) in Richman et al (4) for U51(vGPCR1) in Long et al (1) for E5 (vGPCR5) and E54 (vOX2-1) in Oo et al (29), in U48.5 (TK) and U48 (gH) (= CD-II) in Zachariah et al (28) and also for E39 (ORF-Q) (= CD-III) in Fuery et al (7). On the other hand, details about the levels of variability and subtype clustering for several other loci e.g. CD-VI (E30/31),

CD-VII (E23/24/25) as well as for E37 (ORF-O) and E38 (ORF-P) and especially about the multiple alternative triplex gene 'cassettes' within the R2-segment of EEHV1 strains have not been described previously.

Our analysis of these new cases included a full evaluation of the single novel EEHV1 (Umesh, EP55) strain involved and its patterns of divergence at both the intact genomic and single protein-coding gene levels from other previously encountered or published EEHV1 reference strains.

Materials and Methods

Ethical statement

Viremia samples, tissue samples and sera from elephants were analyzed by order of the contributing zoos. Ethical approval was not sought as the present study only made use of samples that were taken for specific diagnostic purposes, the results of which are described in this manuscript. The samples were taken under routine veterinary care by veterinary staff. The contributing zoos as well as their scientific advisors consider it important to sharing critical medical, husbandry, training, and scientific information with colleagues across the globe.

Elephants

At the time of the outbreak, the herd of Asian elephants (*Elephas maximus*) consisted of eight animals, divided into two family lines and one breeding bull, who had been introduced from outside in 2014. Also in 2014, the management of the elephants was changed from "hands on" to "protected contact" principles, which implied that all elephant-human interactions, including all methods of sampling, were determined by the individual elephant's decision to interact (30). The two family lines lived separately but were able to directly interact with each other. Fig 1 gives an overview of the relationships between the individual elephants as well as their life span and origin, if they were born outside of the Zurich zoo. In addition to the present animals, three additional males are indicated in the figure, one (Maxi) being the

former breeding bull and the other two representing two previous cases of EHD (Xian, EP07 and Aishu, EP16, respectively) in the same zoo.

The first family line, whose founder Ceyla had been born in 1975 in Sri Lanka and transferred to the zoo in 1976, consisted of three individuals, including the founder's daughter Farha and her daughter (Ruwani), born to Farha and Thai, the new breeding bull. Notably, Xian and Aishu had also been members of this family line but had been fathered by the previous breeding bull Maxi.

The second family line, whose founder Indi had been born in 1988 in Myanmar and transferred to the zoo in 1999, consisted of four individuals, including the founder's two daughters from Maxi (Chandra and Omysha) and her recent son (Umesh) from Thai.

Thus, all individuals except of the two founders and the bulls had been born in the Zurich zoo.

Fig 1. **Family trees of the elephants relevant for the present study**. Green and blue: founder females with year and country of birth. Red and orange: breeding bulls. Multicolored: color coded offspring with year of birth and year of death (if applicable). Two prior cases of EHD (Xian, Aishu) are also integrated. EHD in brackets: animal was lost to EHD.

Samples

Viremia samples. For weekly viremia screens, one blood droplet (approx. 20 µL) was drawn from the elephant's feet during pedicure by using a small capillary, which then was placed into an Eppendorf tube containing 80 µL nuclease-free water and thoroughly mixed. Due to the "protected contact" principles, the animals chose to occasionally refuse the sampling procedure. Omysha's sampling period started on January 21, 2018, whereas sampling of Ruwani and Umesh started on July 3, 2018 and February 3, 2021, respectively. Routine screening ended with the first detection of serious EEHV1 viremia in Umesh's sample on June 23, 2022.

Historic EDTA blood samples from EHD cases in 2018 (H1 Anjuli and H2 Kanja) were kindly provided by the Hagenbeck zoo in Hamburg, Germany. Cellular fractions were taken as source of DNA extraction for PCR, whereas plasma fractions were used in serology.

Serum samples. Sera from Swiss elephants are routinely collected and archived for health monitoring purposes. Samples from these archives were provided for serological analyses by order of the Basel zoo and in the Zurich zoo, respectively. Samples from the Zurich zoo were collected throughout March 2022. Four sera (A1, A2, A3, A4) from African elephants in Basel zoo were collected throughout January 2022.

Tissue samples. After necropsy of each of the three elephants, 15 mg from each of five tissues (heart, spleen, liver, kidney, lung; kindly provided by Dr. Monika Hilbe, Institute of Veterinary Pathology, University of Zurich) were combined from each individual and minced with 300 µL of body fluid and 300 µL blood using a TissueLyser II (Qiagen).

In addition, a few slices of historic heart and liver tissues from Xian, son to Maxi and Ceyla, who had been lost to EHD in 1999 (partially sequenced genome in GenBank as Xian, EP07), were kindly made available for the present study by Prof. Franco Guscetti, Institute of Veterinary Pathology, University of Zurich.

Real-time PCR detection of EEHV1, EEHV4, and ETNF

The previously described real-time PCRs for EEHV1 (targeting U41, the major DNA-binding protein locus) and EEHV4 (targeting U60, the terminase subunit 1 locus), respectively, ~~had been designed for broad range applications,~~ identifying all known subtypes of a particular virus type (12, 25). The elephant TNF-alpha (ETNF) locus was additionally targeted for two purposes, (1) to test for amplifiable elephant DNA in the sample and (2) as a means to quantify the relative amount of viral DNA in the sample compared to host DNA (12). Briefly, 5 µL aliquots from diluted blood droplet samples were used as template per well. In addition, each 20 µL reaction volume contained 10 µL PrimeTime Gene Expression Master Mix (Integrated DNA Technologies), 0.9 µM of each primer and 200 nM probe, and 5 µL template or nuclease-free water as negative control. Samples were tested in duplicate on a QuantStudio 3 Real-Time PCR System (Applied Biosystems) using the following cycling conditions: 2 min at 50°C, 10 min at 95°C, 40 cycles of 15 sec at 95°C and 1 min at 60°C.

A test was considered valid if ETNF was detected at a Ct value of 39 or lower and if the negative controls for each assay did not yield a Ct value. To compensate for sample variation in volume and elephant cells, the Ct value of ETNF was used as an approximate measure for the number of elephant cells within the sample. ~~Although~~, the virus target is present at one copy per virus genome, and the host target is present at two copies per diploid genome, the ETNF value is proportional to the cell number in the assay and, thus, can be used to measure the proportion of viral genomes and cellular genomes, ~~respectively~~. Therefore, the Ct value of the ETNF signal was subtracted from the Ct value for EEHV to get a ΔCt value (ΔCt = Ct_{EEHV}-Ct_{ETNF}). Accordingly, the ΔCt values grew on the negative side with increasing numbers of EEHV genomes per cell.

Conventional PCRs

Sequence analysis of conventional PCR products can provide rapid information about the identity of the current virus strain, even very early during an outbreak. In a first round, ~~we used~~ a previously established PCR targeting the E36/U79 locus to discriminate between EEHV1A and EEHV1B strains (25). In a second round, ~~we targeted~~ the E54/EE51 vOX2-1 locus, which is expected to remain stable in separate isolates from the same source, while isolates from differing sources may differ from each other by diverging up to 15% at both the nucleotide and the aa level (28). The oligonucleotides used in these PCRs are listed in Table 1. The PCRs were run in a volume of 20 µL, comprising 5 µL template, 10 nM dNTP, 5x Phusion GC buffer, 2 U of Phusion High-Fidelity DNA Polymerase (Thermo Scientific) and each primer at a concentration of 0.5 µM (Table 1). After an initial denaturation for 2 min at 98°C, the samples were cycled 39 times at 98°C for 10 sec, 58.4°C for 20 sec, and 72°C for 7 sec. The runs were completed with 10 min at 72°C before cooling to 10°C. The PCR products were evaluated for their size by agarose gel electrophoresis before being extracted from the gel for cycle sequencing by a commercial company (Microsynth, Balgach, Switzerland).

Table 1. **Oligonucleotides used for conventional PCR**

Target	Oligo	5' to 3' sequences	Amplicon size
E36/U79	F-E36<br>R-E36	TCC AGG GAT TTC TCC AGT TG<br>GCC ACC TTC TTC TGC TTT TG	126 bp
E54/EE51	AttB1_vOX_R1	GGGGACAAGTTTGTACAAAAAAGCAGGCT<br>ATGCT[T/**C**]CAGAGAAAGTACAGGTAC[a]	930 bp
	AttB2_vOX_L1	GGGGACCACTTTGTACAAGAAAGCTGGGT<br>GTGTTGCCG[T/**C**]CACGATGC**C**TTCTACG[b]	

The primers targeting the vOX2-1 locus, encoded by E54/EE51, were elongated by att-recombination sequences (grey) for facilitated cloning and sequencing after PCR amplification. Moreover, an equimolar mixture of two different forward (R1 with wobbling at position 6)[a] and reverse (L1 with wobbling at position 10)[b] primers was used to compensate for published sequence variations at this site.

LIPS assays

LIPS antigens. The selection and production of LIPS antigens was modified from a previously published protocol (7). Antigen for LIPS serology was harvested from transfected COS7 cells, using synthetic and codon-optimized DNA (GenScript Europe, the Netherlands). For this purpose, the published amino acid sequences of E39 clades A (Kimba), B (Emelia), C (Raman), and D (Daizy) encoding ORF-Q (7) as well as of the gB glycoprotein (EEHV1A, Kimba, (18)) were reverse translated, codon-optimized for expression in human cells, and cloned under the control of the cmvIE promoter into the pcDNA3.1(+) vector. Moreover, each construct was fused with the following sequence of C-terminal tags: V5-epitope, nanoLuciferase (nLuc), and 7-his. Except for ORF-Q clade D, each construct possessed its native signal peptide, which was included into the gene synthesis design. As clade D was not predicted to possess a functional signal sequence but two short transmembrane regions close to the N-terminus, the molecule was shortened to start only at its original amino acid No. 60, which was preceded by the clade A signal peptide. Expression of the desired antigens following transfection was verified by measuring nLuc activity (relative light units, RLU) in the cell culture supernatants as well as by detecting the corresponding proteins in cell lysates after standard Western immunoblotting using a commercial anti-V5 monoclonal antibody (SV5-Pk1 from Invitrogen).

LIPS assay. The protocol for the LIPS assay was adapted from a previously published overview article (31). A commercial kit (Immunoprecipitation Kit, Abcam, Cambridge, UK),

which provided buffers for protein extraction and solubilization as well as Protein A/G sepharose beads and washing buffers, was used according to the supplier's instructions. Briefly, the secreted antigens were harvested from the cell culture supernatants and mixed with non-denaturing solubilization buffer (NDSB) and protease inhibitors before non-soluble proteins were removed by centrifugation. Prior to the assay, the nLuc activity of each antigen was adjusted to comprise 10^7 RLU per second (RLU/s) in a volume of 50 µL (NanoGlow, Promega, Madison, Wisconsin, USA). The sera were prediluted with phosphate buffered saline (PBS) to 1:100 in a polystyrene microtiter plate (Nunc F96 MicroWell flat bottom) and mixed well by shaking. These were kept in these plates at -20°C for a maximum of three weeks. A second polystyrene plate was used for the actual assay. First, 40 µL of NDSB were pipetted into each well before 10 µL of prediluted sera and 50 µL of freshly diluted nLuc-antigen were added. The mixture was incubated by shaking (300 rpm) for one hour at ambient temperature. Meanwhile, a slurry comprising 30% sepharose A/G beads was prepared in PBS and 5 µL were added to each serum sample to incubate for one more hour with shaking. Eventually, all samples were transferred to a filter plate (MultiScreen, Merck, Zug, Switzerland) and washed on a vacuum manifold. Each well was washed eight times with 100 µL of wash buffer, followed by two times washing with PBS. Following the last wash, the vacuum was turned off. The filter plate was removed and blotted dry using a stack of filter paper making sure to remove moisture on the top and bottom of the plate. Eventually, 50 µL of NanoGlow substrate (Promega) was added to each well before measuring in a luminometer (MicroLumat Plus, LB 96 V, up-version 2.0; software WinGlow v. 1.25.000003, Berthold Technologies, Bad Wilbad, DE). Since horses are expected to be resistant to EEHV-infections and, therefore, free of antibodies against any EEHV, two horse sera (P1 and P2), both known to have antibodies against equine herpesvirus 1 (EHV-1) were used as controls to determine the negative cut-off values of each test. As no true EEHV-negative elephant sera are

available, the cut-off decisions were based on a previous publication on marine turtle serology, ~~where a similar problem exists~~ (32).

Sample Preparation and DNA Sequencing

DNA was extracted from necropsy samples for whole genome sequencing. A previously established viral particle enrichment protocol was used to increase the recovery of viral genetic material (33). ~~Then, e~~enrichment of viral nucleic acids was followed by reverse transcription for any RNA viruses (to exclude other causes of death) and sequence-independent single primer amplification for DNA. The nucleic acids were sheared to 500 bp length using the E220 Focused-ultrasonicator (Covaris, Woburn, MA, USA) and prepared with the NEBNext Ultra II DNA Library Prep Kit for Illumina (New England Biolabs, Ipswich, MA, USA) according to the manual. Sequencing libraries were sequenced at the Functional Genomics Center Zurich (FGCZ) in a paired-end 2 × 150 bp, SP flow cell sequencing run using the NovaSeq 6000 (Illumina, San Diego, CA, USA). The PhiX Control v3 Library (Illumina, San Diego, CA, USA) was added as the control.

Data analysis

Sequenced reads were analyzed using the *de novo* assembly pipeline described previously (34). Illumina sequencing adapters and low-quality sequencing ends were trimmed using Trimmomatic (v. 0.39) (35). Subsequently, trimmed reads with average quality above 20 and longer than 40 nt were further cleaned up by trimming away the SISPA primers using cutadapt (v. 3.2). The quality-checked reads were then assembled using metaspades (v. 3.12.0) (36). Assembled *de novo* contigs were compared to the National Center for Biotechnology Information (NCBI) non-redundant database (nt) using BLAST-N (v. 2.10.1+) and annotated using the best BLAST-N hits. Quality-checked reads were mapped back to the assembled sequences using bwa (v0.7.17) mem (37). In addition, contigs from EEHVs were aligned in a metagenomic pipeline of the SeqMan NGen v.17 (DNAStar, Lasergene, Madison, WI, USA) to all available viral genomes of other EEHVs downloaded from the

NCBI database (08.2022) and visualized in the SeqMan Ultra (DNAStar, Lasergene, Madison, WI, USA).

To determine genome similarity values between our assembled strain and several other available complete EEHV1A strains, as well as EEHV1B (Emelia), and EEHV6 (North American Proboscivirus case number 35; NAP35) we performed BLAST-N analysis using the VIRIDIC tool (settings: '-word_size 7 -reward 2 -penalty -3 -gapopen 5 -gapextend 2') (38).

Annotation of EEHV Genomes

Typical EEHV genomes comprise about 120 genes (1, 11, 12, 19). Almost 40 of these are also found as conserved but characteristically diverged versions within the majority of other mammalian herpesvirus sub-families and are referred to as “core” genes with an applied U number nomenclature system derived from that used originally for human HHV6 genome analysis (39).

The remaining 80 or so *Proboscivirus*-specific protein encoding genes that have not been found in any other herpesviruses have been given a sequential unique E number nomenclature system based on that originally applied to the prototype EEHV1A (Kimba) genome (4, 5, 12). Universally agreed upon three-letter abbreviations were used to name proteins with previously available functional or homology data from other well-studies herpesvirus species. Otherwise, we used a combination of either an extended ORF-A to ORF-S system as originally suggested by Ehlers et al (16) or just left them with the same E number as for the designated gene name where little else is known for a novel *Proboscivirus*-specific protein. In terms of detailed gene-by-gene analyses, there are two alternative published physical genetic maps of prototype EEHV1A genomes that Umesh can be compared to with that of EEHV1A (Raman) from Wilkie et al (17) being drawn in the opposite orientation and using a different (EE number) gene nomenclature system. Furthermore, the Umesh genome described here and most other intact annotated AT-rich branch genomes of EEHV1, EEHV2A, EEHV5B and

EEHV6 strains in GenBank have been given the same orientation and E number nomenclature as that used for Kimba (18), except for having their R2-segments switched to the extreme left-hand-side compared to our original inadvertent placement on the right-hand-side within a linearized version of the circular EEHV1A (Kimba) genome after the actual packaging cleavage site in Raman DNA was determined (17). Curiously, none of the GC-rich branch *Probosciviruses* EEHV3A, EEHV3B nor EEHV4A or EEHV4B complete genomes even have an R2-segment (19). Note that based on the precedent from Fuery et al (7) of using the term "clades" rather than "subtypes" for multiple E39 (ORF-Q) gene variants, as well as for their corresponding ORF-Q antigens and antibodies, ~~we have used~~ that descriptor ~~throughout this paper, but only~~ for this particular locus because of its now proven usefulness for differential detection of particular EEHV1 strains in serological and immune response assays.

Sequence sources

Publicly available nucleotide as well as protein sequences from NCBI were used for comparative analysis of our data. For the full genomic nucleotide sequences of reference strains used, their case numbers and names as well as origins and year of collection of the individual infected elephants and sources to reference sequence information are listed in Table 2**.** Otherwise, the sources of selected relevant protein and nucleotide sequences used from incomplete sub-genomic loci are provided in the corresponding text passages or figures. Similarly, for those EEHV cases associated with supplementary Table S1 that data for both complete genomes and relevant sub-genomic PCR DNA sequence file accession numbers is provided in Table S2.

Table 2. Used EEHV full genomic sequences

Virus	Case[1]	Individual	Origin	Year	Source
EEHV1A	NAP23	Kimba	USA	2004	KC618527.1
EEHV1A	EP24	Raman	UK	2009	KC462165
EEHV1A	EP55	Umesh	Switzerland	2022	OR543011
EEHV1B	EP18	Emelia	UK	2006	KC462164

EEHV6	NAP35	Miss Bets1	USA	2009	MZ822422

[1]NAP#, North American *Proboscivirus* case number; EP#, European *Proboscivirus* case number

Results

Weekly screening for EEHV-viremia

Between January 2018 and June 2022, capillary blood of the juvenile elephants was analyzed on a weekly basis by TaqMan real-time PCR for the presence of EEHV1 and EEHV4 DNA, respectively. The results of this monitoring program are summarized in Table 3. The average Ct value for ETNF amounted to 31.5 with a standard deviation of 3.1. Although rarely and at low titers (average Ct values ranging between 35.3 and 40), transient (never on subsequent testings) and subclinical viremia by EEHV1 was detected in two of the three monitored animals. Notably, EEHV4 DNA was never detected throughout the screening period.

Table 3. Results of TaqMan real-time PCR targeting EEHV1, EEHV4 and ETNF.

Animal	Screening	Tests (% valid)	EEHV1	EEHV4
**Omysha**	231 weeks	211 (98.2%)	6 times pos	Never detected
**Ruwani**	209 weeks	189 (99.5%)	3 times pos	Never detected
**Umesh**	60 weeks	59 (98.3%)	Never detected	Never detected

EEHV serology using LIPS assay

Based on the negative cut-off value determined by horse sera, a LIPS assay using the conserved glycoprotein B of EEHV1 (gB1) as antigen, revealed that all adult individuals possessed antibodies against gB1 (Fig 2A). Interestingly, even adult African elephants reacted positively in this assay, which can be attributed to the close antigenic relationship (89% amino acid identity) of gB1 and EEHV6-gB (gB6). In contrast, the sera of two young elephants from Germany who previously had succumbed to EHD did not reveal such antibodies. Interestingly, two of Zurich elephants at risk (Omysha; Umesh) had detectable levels of anti-gB1 antibodies in their sera, whereas the third animal (Ruwani) appeared seronegative.

Next, the elephant sera were analyzed against ORF-Q clade A, B, C, and D antigens (Fig 2B-E). In general, the antibody levels against the ORF-Q antigens were considerably lower than against the conserved glycoprotein B. However, at least one individual (Indi) reacted clearly above background levels against ORF-Q clade A antigen, whereas two individuals (Omysha and Thai) were clearly seropositive against ORF-Q clade B antigen. Moreover, two of the individuals at risk (Indi, Chandra) reacted positively against ORF-Q clade C antigen. Interestingly, two of our control sera (one of the German individuals who had died from EHD and one African elephant) also reacted above the background level. Although at least two individuals (Indi, Ceyla) had been present during a previous outbreak of an ORF-Q clade D virus, none of the individuals at risk was presently deemed seropositive against ORF-Q clade D antigen.

As all negative cut-off values were calculated by using horse sera, it seemed sensible to analyze the same panel of sera against an antigen (glycoprotein D, gD), which is suitable to detect antibodies against equine alphaherpesvirus 1 (EHV-1), a highly prevalent pathogen of horses. Indeed, both horse sera tested reacted positively, whereas all elephant sera had no detectable antibodies against the equine viral antigen (Fig 2F).

Together, these serological analyses confirmed both the presence and replication of various EEHV1 subtypes among the animals at risk.

Fig 2. **Prior antibody responses against EEHV1 antigens.** LIPS assays were performed to detect antibodies against various EEHV1 antigens in the sera of elephants and horses. In each panel, the individual elephants are lined up on the x-axis, whereas the reaction of their sera is given in relative light units (RLU) on the y-axis. Yellow bars: Members of the Indi family; green bars: members of the Ceyla family; grey bar: Thai, most recent outside acquisition to the herd, introduced in 2014; red bars: sera from previous EHD cases in the Hagenbeck zoo, Hamburg, Germany; mandarin-colored bars: African elephants from the Basel zoo, Switzerland; blue bars: horse sera, which were used to determine the negative cut-off value of the test.

Panel A. Antibody responses against the conserved glycoprotein gB1; **Panel B**: antibodies against ORF-Q clade A; **Panel C**: antibodies against ORF-Q clade B; **Panel D**: antibodies against ORF-Q clade C; **Panel E**: antibodies against ORF-Q clade D; **Panel F**: antibodies against equine alphaherpesvirus 1 gD.

Viremia upon EHD

On June 16, 2022, all three of the monitored animals tested negative for both EEHV1 and EEHV4, but one week later, on June 23, Umesh tested EEHV1-positive with a ΔCt value of +3.8. Samples from the two following days confirmed its increasingly viremic status with ΔCt values of -0.75 and -2.75, respectively, upon which intravenous GCV treatment was initiated. Nevertheless, the ΔCt value of the same animal dropped to -4.1 and to -4.65 before coming back to +0.5 at the day of ~~its~~ death ~~due to EHD,~~ five days after initial detection of viremia (Fig 3). The other two animals remained EEHV1-negative until slight viremia was first detected on ~~June 26. Both went back to negative~~ in their next two samples. On July 3, Omysha's ΔCt value was +6.15 and, after a brief recovery dropped to -1.4 before the animal died 10 days after initial detection of viremia. Beginning from June 30, Ruwani went forth and back with short periods of viremia. Eventually, the ΔCt also dropped to negative values ~~and this animal was also lost to EHD~~ within four weeks (Fig 3).

Fig 3. Individual EEHV1 loads before and throughout development of EHD. Delta Ct values are shown on the y-axis, whereas the date of blood sampling is given on the x-axis.

Analysis of the causative EEHV

Sequencing of the E36/U79 locus

~~Having established an ongoing viremia with EEHV1,~~ a conventional PCR targeting the E36/U79 locus showed (Fig. 4) that the resulting sequence obtained from an early Umesh sample was 100% identical to Xian's previous EEHV1A isolate. In contrast, 11 different nucleotides in comparison to Omysha's previous EEHV1B isolate were observed, which identified the current isolate clearly as EEHV1A.

Fig 4. **Sequence alignments**. Alignment of the present E36/U79 sequence (Umesh, EP55) against previous ones. (A) Comparison against the 1A subtype (Xian, EP07). (B) Comparison against the 1B subtype (trunk wash from Omysha).

Initial analysis of the E54/EE51 vOX2-1 locus

Having established that Umesh had been affected by an EEHV1A strain and knowing Xian's E54/EE51 vOX2-1 sequences from 1999 (GenBank: MH287538), ~~it was of immediate interest to determine the identity level among the two viruses. Therefore, we amplified~~ DNA

from Umesh's blood sample by conventional PCR targeting the E54 locus. Sanger sequencing of the PCR product yielded 840 nucleotides, which were compared against Xian's previously determined vOX2-1 sequence as well as against an American (Kimba, 2004) and a European EEHV1A strain (Raman, 2009) and rooted against the subtype 1B prototype (Emelia,2006) (17, 18). On the nucleotide level, the vOX2-1 sequences of the present Umesh isolate (2022) shared only 92.34 – 93.84% identity with any of the compared sequences, corresponding to a minimum of 54 to a maximum of 67 nucleotide polymorphisms per sequence. The closest relationship was observed with the European 1A prototype (Raman), the least with the American 1A prototype (Kimba) (Fig 5). Overall, the present Umesh isolate differed clearly from the previous Xian isolate.

Fig 5. vOX2-1 phylogenetic tree. A phylogenetic tree was obtained from the alignment of the nucleotide sequences of the vOX2-1 locus (E54/EE51) from the American prototype of EEHV1A (Kimba), the European EEHV1A prototype (Raman), the European prototype of EEHV1B (Emelia) in comparison to the corresponding sequences of the present case (Umesh) and the 1999 case of EHD in the Zurich zoo (Xian).

Determination of the full viral sequences from all three cases

Eventually, the full sequence of the present EEHV1A virus was determined from tissue samples after necropsy by a next-generation Illumina paired-end procedure Sequences covering the complete viral genome were obtained from Umesh's samples, while the sequencing data obtained from samples from Omysha and Ruwani were less complete. Upon comparing the contigs obtained with the Umesh genome against the contigs from Omysha and Ruwani, 27,488 and 4,679 nucleotides, respectively, were unreadable in the two incomplete EEHV genomes. Excluding non-sequenced fragments, there were also 56 ambiguous positions in the Ruwani data, but otherwise the three genomes were virtually identical within the sequenced blocks. All raw sequencing data generated were uploaded to the Sequence Read Archive (SRA) as BioProject ID: PRJNA1052582. The main focus of our further analysis was on Umesh's complete genome.

Overall genome structure and gene content of EEHV1A (Umesh, EP55)

The size of the assembled single complete intact genomic scaffold of Umesh (EP55) was 179,847-bp (annotated as GenBank accession number OR543011). This included two near identical copies of the 2.9-kb direct terminal redundancy (TR), one located at each end. ~~Probably the most distinctive features of EEHV1 genomes are~~ the two large-scale (20-kb and 40-kb) inverted core gene segments compared to cytomegaloviruses, the putative inverted repeat stem-loop origin of lytic DNA replication (ori-Lyt, 1190-bp), the two most likely novel nuclear immediate-early transcriptional control proteins E40 (ORF-K, 743-aa) and E44 (ORF-L, 1209-aa) and the largest likely tegument protein E34 (ORF-C, 1886-aa), which are all present and intact with close to the same structures and aa sequences as those found in all previously studied EEHV1 genomes. Other ~~unusual but~~ consistent features of EEHV1 strains include the likely captured cellular genes E4 (vGCNT1) and E47 (vFUT9), plus four versions of vOX2-like proteins E23 (vOX2-4), E24 (vOX2-3), E25 (vOX2-2), and E54 (vOX2-1), as well as the UL9-like U73 (OBP) and both U27.5 (RRB) and U48.5 (TK) (also all missing in cytomegaloviruses) and eight conserved *Proboscivirus*-specific vGPCR-like proteins including E3 (vGPCR6), E5 (vGPCR5), E15 (vGPCR4), E20 (vGPCR4A), E21 (vGPCR4B), E26 (vGPCR3), U12 (vGPCR2) and U51 (vGPCR1) within a large family of 26 retinoic acid inducible protein 3 (RAIP3)-like 7xTM domain receptors. These are all present within the Umesh genome and with just a small number of exceptions, the same applies to the majority of the rest of the expected total of close to 120 genes of the virus.

Fig 6. Similarity heatmap. BLAST-N alignment of the complete Umesh (EP55) genome against closely related EEHV genomes in the GenBank database using the VIRIDIC tool. The heatmap shows the similarity values for each genome pair, the fraction of genomes aligned and the genome length.

Comparison with other EEHV1A and EEHV1B genomes

Previous experience in EEHV1 whole genome analysis has revealed that all individual independent strains display high levels of overall genetic divergence associated with a large subset of hypervariable genes. Across the entire population of EEHV1 strains examined the

most striking feature is that many of these variable genes themselves cluster into multiple highly diverged cladal subsets that have been designated as specific subtypes. Furthermore, most (but not all) EEHV1 variable genes often show these large and complex differences at both the protein (amino acid) level as well as at the DNA (nucleotide) level. Figure 6 shows the results of a search for the overall level of intergenomic DNA sequence divergence, ~~which revealed that~~ the closest match among the other nine complete EEHV1A genomes contained within the GenBank database (May 2024 version) is the Indian strain EEHV1A (IP43) [MN366291]. ~~The latter differs~~ from Umesh (EP55) by 2.7% at the nucleotide level (4,856-bp mismatch) as determined by direct pairwise comparisons using the VIRIDIC tool (38). It also differs from ~~our~~ prototype EEHV1A (Kimba) genome [KC618527] by 4.6% (9,712-bp mismatch) and is ~~least closely~~ related to the EEHV1A (IP165) genome [MN366292] with 7.5% divergence (13,488-bp mismatch). All of the other intact EEHV1A genomes fall within the range between IP43 and IP165. For the prototype chimeric subtype EEHV1B (Emelia) genome [KC462165] from Asian elephants the divergence is 8.2% (14,747-bp mismatch) and for the orthologous prototype EEHV6 (NAP35) genome [MZ822422] from African elephants the divergence is 26.2% (47,120-bp mismatch).

Analysis of open reading frames and likely splicing motifs within the EEHV1A (Umesh, EP55) complete genome revealed that it encodes a total of 121 predicted viral proteins that are named and listed under GenBank accession numbers WNZ34470.1 to WNZ34590.1 including 17 that involve in-frame splicing. After detailed BLAST-X and BLAST-P analysis plus clustal-W and visual six-frame inspection of all potential individual ORFs at the DNA and protein level relative to all other well-characterized EEHV1 strains, the Umesh genome proved to display similar features and gene organization as well as the typical patterns of gene content divergence, but ~~it~~ does not have any extra or novel genes not seen before in at least a subset of EEHV1 strains. Overall, Umesh consists of a complex mosaic pattern of mostly highly-conserved proteins intermingled together with a distinctive and unique pattern of

subtypes for the 30 or so variable proteins typically found across EEHV1A genomes (1, 28), but has none of the characteristic features typical of EEHV1B diaspora strains (4). Although EEHV1B (Emelia) is the only completely sequenced member of the 1B subtype there are seven other partially characterized EEHV1B strains that have nearly identical EEHV1B-specific characteristics at each of these same three CD loci. On the other hand, Umesh does contain the novel E36A (ORF-N) vCXCL chemokine-like protein. The latter is present in all EEHV1A strains as well as in EEHV2, EEHV5 and EEHV6, ~~but is absent from all EEHV1B strains as well as from EEHV3 and EEHV4~~.

Comparison with main segment EEHV1 variable gene subtype assignments

~~A list of the~~ EEHV1A (Umesh) assigned subtypes at each of the 20 most variable protein loci from the main segment of the genome i~~s shown in the upper Section I of supplementary Table S1~~. The particular virus genomes chosen as illustrative examples here include the original three complete genome prototypes from GenBank of EEHV1A (Kimba), EEHV1A (Raman) and EEHV1B (Emelia), plus accumulated sub-genomic locus data from six of the best characterized but incomplete EEHV1A genomes determined previously by conventional PCR amplification-based DNA sequencing of older lethal EHD cases as carried out by the Johns Hopkins University Viral Oncology group. Specifically, this data was derived from one sample from India (IP11), one from Europe (EP07, Xian) and four from the United States (NAP18, NAP26, NAP17 and NAP21).

Other key details about these ten EEHV1 strains are listed in Table S2**,** including where appropriate GenBank accession numbers for complete genomes as well as for six selected representative sub-genomic loci. With the exception of the prototype EEHV1A (Kimba) strain, which (although it does have three Cs within CD-II) was otherwise designated to have just A or A1-subtype variable genes across the entire genome, all other non-1B diaspora

strains fall into complex mosaic patterns like this with largely unlinked variable gene subtypes.

The most useful region to evaluate here has proven to be the combined ORF-O/ORF-P/ORF-Q complex (part of CD-III) for which we recognize a total of thirteen different inserted alternative three-gene patterns, with the same C-E-C subtypes as in Umesh being found also in eleven out of a total of 52 other EEHV1A or EEHV1B strains that have been fully evaluated previously at this locus. Those include the viruses from European cases Raman and Plai Kiri as well as from IP43, NAP16, NAP24, NAP43, NAP73, NAP75, NAP88, NAP91 and NAP93. Although only two of those examples are available as complete genomes (Raman and IP43), none of them occur within known EEHV1B strains. In addition, only one of the seven India cases evaluated here (IP43) has that same C-E-C group pattern.

Comparison with other EEHV1 R2-segment gene arrangements

Similarly, many of the eleven potential protein coding positions located within the short 10.5-kb R2-segment of all EEHV1 genomes display high levels of variability and subtyping that have proven to be completely unrelated to the linked 1A/1B chimeric domain pattern explained above. These latter non-1A/1B diaspora related hypervariable gene loci all map within the novel *Proboscivirus*-specific segments located near both ends of the linear EEHV1 genomes. Although the R2-segment mapping between E47 (vFUT9) to E55 (vIgFam3) encompasses just 5% of the length of the EEHV1 genome, it has multiple levels of additional complexity including involving alternative three-gene “cassettes” that define eight distinct clustered R2 sub-groups designated A, B, C, D, E, F, G and H. Each strain carries just one of these triplex cassettes and there are three different potential locations where they can be inserted. Therefore, these clustered groupings represent major determinants of EEHV1 genomic organization.

Unlike the 20 known available “optional” R2-family genes present within the cassettes, which are each found in just a small subset of the overall EEHV1A strain population, there are also eight remaining “constant” R2-segment genes (see Table S1) that are almost always all present but are also mostly highly diverged and form many additional subtype clusters of their own. The latter include two interesting captured host genes E47 (vFUT9) and E54 (vOX2-1) plus six members of multigene paralogous R2-segment gene families.

Although each individual EEHV1 genome usually contains a total of just eleven genes (often including an average of two or three fragmented or inactivated ORFs) within the entire R2-segment, the overall population of 38 distinct independent EEHV1A plus EEHV1B virus strains that have been evaluated fully across the R2-segment encodes a total of 28 different available alternative genes. The presence (+) or absence (-) as well as the relative location and subtype designation of 20 of these alternate genes found within the R2-segments of either Umesh itself or one or more of the set of nine reference EEHV1 genomes are summarized in Section II of the supplementary Table S1**.** Note that only four of the total of eight known organizational patterns of alternative R2-segment insertion cassettes are represented here (i.e. A, B, D and F groups, but not any examples of the C, E, G or H groups).

These other R2-segment genes include seven more alternative versions of vGPCR-receptors, namely E48 (vGPCR7), E50.5 (vGPCR8/vGPCR9/vGPCR13), E56 (vGPCR12), E59 (vGPCR10 and E62 (vGPCR11) from within the large 7 x TM RAIP3-like family that each occurs in just a subset of strains. Every EEHV1 R2-segment includes either just one or two of the latter with Umesh itself having versions of both E50.5 (vGPCR9) and E59 (vGPCR10) but not any of the others. There are also a total of 18 paralogous diverged alternative versions of mostly membrane-bound immunoglobulin-domain (Ig) family proteins (designated vIgFam1 to vIgFam15) distributed amongst known EEHV1 strains. Umesh encodes its own versions of five of these, including three that are constant e.g. E50 (vIgFam1), E53(vIgFam2.5) and E55 (vIgFam3) plus two of the alternates E60 (vIgFam6) and E61

(vIgFam7). Some of these are detectably related to host cell protein CD48. Finally, there is also a small third family of four other rather non-descript alternative R2-segment membrane proteins (E49, E51, E64 and E68).

From among the total of 28 known alternative R2-genes, the Umesh pattern most closely matches that of the Ganesh (NAP26) and IP43 by having a typical group-D E59/60/61 three-gene triplex cassette among the total of eleven R2-segment encoded proteins overall (see Table S1). Unlike the other R2 genes described here three of the vIgFam proteins (E60, E61 and E53) do not show any significant subtype variability, whereas in contrast the 43 examples of E54 (vOX2-1) proteins evaluated despite collectively exhibiting up to 15% overall variability at the aa level do not cluster into any readily definable subtype groupings. In contrast there are just two tight major subtype clusters of E47 (vFUT9) in which the prototype EEHV1A (Kimba) version (subtype-A) and the prototype EEHV1B (Emelia) version (subtype-B) differ by as much as 32% at the protein level. Nevertheless, this locus shows no obvious linkage to the classic 1A/1B-subtype diaspora. In fact, from among the 35 total E47 (vFUT9) enzyme-encoding versions evaluated here there are 24 examples of the B-subtype (including Umesh) and only eleven of the A-subtype, with both being randomly assorted among EEHV1A and EEHV1B diaspora genomes.

Overall, the eight different alternative three-gene inserted cassette groups provide the most definitive way to classify EEHV1 genome patterns within the R2-segment. Here the Umesh strain clusters within the largest set (= group-D) along with ten other group-D examples (namely Daisy, IP43, Xian, EP20 = EP21, NAP30, NAP31 and NAP41 = NAP47), including three from Europe (Xian, EP20 = EP21), but just one from India (IP43). Moreover, there are no known group-D inserted cassette patterns (i.e. E59/E60/E61) that fall amongst the six EEHV1B genomes for which R2-segment data is available. The next two most abundant cassette patterns are seven group-As and six group-Bs, with the remaining 15 examples being three -Cs, two -Es, four -Fs, two -Gs and four -Hs. Among the latter, there are several other

anomalous situations where a total of ten virus strains display either just a two-gene cassette (groups-C, -E and -G) or four others have both a group -B like three-gene cassette plus a two-gene group -H cassette (not shown).

~~Finally, for completeness note that~~ Section III of Table S1 includes examples of subtype designations applied previously to five core conserved genes from these and many other EEHV1 genomes based on selected small PCR-sequenced loci including E38 (POL) that just display low nucleotide level variability without significant associated protein level variation (1, 3, 4, 35).

~~Overall,~~ the sequencing results confirmed that all three EHD cases here had been infected by the same novel strain of EEHV subtype 1A. ~~More specific Umesh data relating to both E39 (ORF-Q) serology and three viral-encoded enzymes of interest in the context of epidemiology and antiviral treatment are presented below.~~

Fig 7. ORF-Q phylogenetic tree. A maximum likelihood phylogenetic tree was obtained from the alignment of ORF-Q amino acid sequences using the Neighbor Joining method and 100 replicates to perform bootstrap analysis. The branch length is indicated for major branches, dividing the tree into the known clades A through F. Each strain is identified by its case number followed by its GenBank accession number.

Further analysis of the Umesh E39/EE3 locus and comparison to other ORF-Q subtypes

Antibodies against a particular ORF-Q clade have previously been associated with a mild course of EEHV1 infection, whereas their absence has been linked to severe EHD (7). ~~Having established that the present virus is different from the two previously observed ones, it was of interest to determine Umesh's complete ORF-Q protein sequence. Furthermore, because ORF-Q proteins clearly have significant and relevant antigenic properties as well as providing one of the most extreme and important examples of EEHV1 divergence and subtyping~~, an updated ORF-Q phylogenetic tree was a~~lso~~ generated showing the alignment results for the intact translated protein sequences from 33 independent strains ~~as presented in Figure 7.~~ The year 2022 Umesh isolate proved to be nearly identical to other ORF-Q clade C isolates such as Plai Kiri and Raman plus NAP43, 73, 75 and IP43), but highly diverged from clade A

(NAP11, 16, 18, 23, 26, 32, 39, 41, 47, 80; IP11, 164), clade D (Xian, EP21; NAP20, 29, 30, 31, 72; IP07) and clade B (NAP45, 49) strains as well as from clade E1 (NAP21), E2 (IP91, 165) and F (NAP17), and further still from EEHV6 (NAP35). Quantitatively, the average percentage protein identity values when compared against the 364 aa version encoded by Umesh as measured by the NCBI BLAST-P program proved to be 98-99% for the other members of clade C, 77-82% for clade A, 45-47% for clade D, 30% for clade F, 28% for clade E2, 26% for clade E1, 26% for clade B and 33% for EEHV6 (NAP35), whose sequence was used as outgroup for rooting the tree. Unfortunately, these results came too late to consider a serum transfer from one of the clade C-positive animals. ~~Together,~~ these epidemiologically relevant data indicate that a previously unrecognized, newly detected strain of EEHV1A had been the cause of the present EHD outbreak.

Analysis of the U48.5/EE7 ETk locus

As the ETk may contribute to the success of antiviral treatment with nucleoside analogues, it was also of interest to compare the predicted protein sequences of Umesh Etk against previously known EEHV1 ETks. ~~Indeed,~~ the aa sequence was highly conserved among reference EEHV1A isolates, with only one aa difference (H278N) against the sequence from the European EEHV1A prototype (Raman) and two aa (V152I and H278N) against the American prototype (Kimba).

This strong aa conservation of the predicted enzyme among a wide variety of EEHV1A strains strongly speaks for a biological role of the Etk, although its *in vitro* and *in vivo* activity against the presently used antiviral compounds remains to be determined.

Analysis of the U69 ECPK locus

The aa sequences of Umesh ECPK are highly conserved when compared to other EEHV1A strains~~. Not a single aa difference from the present sequence from Umesh was found in~~ the sequences of the European EEHV1A isolates Raman or Plai Kiri. One single aa exchange (T409A) was detected upon comparison against the EEHV1B isolate Emelia. The ECPK

sequence of Kimba contained two aa differences, namely T408P and T409P. Since all these differences map outside of the predicted catalytic domain of the enzyme (aa 166 to 306), it will be of interest to address their residual biological functionality in the future.

Analysis of the U38 DNA polymerase locus

The viral DNA polymerase enzyme (POL), encoded by U38 is essential for viral DNA replication. Phosphorylated nucleoside analogues may either block the enzyme thereby terminating its activity or integrate chain-terminating molecules into the growing DNA, which blocks further elongation. ~~Therefore, it was of interest to compare this locus from our three isolates against other published protein sequences in the databases.~~ The sequence conservation among ~~our~~ three isolates at this locus was 100%, whereas two to eight aa changes were observed upon comparison of the Umesh sequence against those from ten previously published cases. ~~Interestingly,~~ Leu 792 occurred in our sequence compared to Isoleucine at this position in all ten previously published isolates (L792I). This seeming mutation mapped to the center of a conserved domain, which had been associated with predicted enzymatic activities, including elongation, DNA-binding and dNTP binding. ~~All other differences had been previously described for other Etk proteins.~~

Discussion

Detailed knowledge of circulating viruses may influence treatment options.

With a history of two prior cases of EHD at the zoo and evidence of recurrent episodes of EEHV shedding and viremia between 2014 and June 2022, the risk for another outbreak of EHD was considered high. In particular, the prevalence of EEHV1A, EEHV1B, and EEHV4 among the adult animals, and the presence of two seronegative animals in the susceptible age were documented (25). During four years of weekly screening, seven instances of low grade,

transient viremia were detected, each caused by EEHV1, while viremia due to EEHV4 was never detected (Table 3).

EEHV serology has only recently established its proper role in addressing EEHV prevalence among elephants (2, 7, 13). Particularly LIPS assays have proved to allow us to discriminate between antibodies that cross-react among various EEHV types or strains and other antibodies that represent a specific immune response against a particular virus strain (7). We selected LIPS assays using the conserved, cross-reactive glycoprotein B- (gB) and the subtype-specific ORF-Q-antigens, respectively, to assess the serological status of the animals against EEHV. The use of ProteinA/ProteinG-beads in the LIPS assays brings a great advantage for the veterinary medical purpose with it because different animal species can be addressed using the same assay. We took this advantage in our case by using horse sera to determine the negative cut-off values for all of our LIPS assays.

All adult elephants had detectable levels of antibodies against gB of EEHV1A (gB1A), which indirectly confirms active circulation of EEHV1 among the elephants. Notably, sera from four African elephants, which were provided for control purposes by the Basel zoo, also yielded varying degrees of antibodies against gB1A. The published protein sequence of the EEHV6 gB (gB6) shares 88 to 89% aa identity with gB1 of the subtype 1A strains from Kimba and Raman, respectively. The protein sequence of gB subtype 1B (Emelia) shares only about 85% aa identity with either EEHV6 (NAP35) or EEHV1A (Kimba, Raman). Consequently, we assume that the gB-antibodies detected in the African elephants were possibly a result of cross-reacting subclinical infection with EEHV6.

The following observations were relevant for the three young animals at risk of EHD: At eight years of age and with a history of shedding EEHV1B (25) as well as several occasions of transient viremia (Tab. 3), the seropositive status of Omysha could be attributed to having mounted her own active immune response against EEHV1. At two years of age and without previously detected episodes of viremia, the seropositive status of Umesh may be explained

by low maternal antibodies, but as half-brother and playing companion to Omysha, it is possible that this individual had already mounted its own immune response against EEHV1 under the previous cover of maternal antibodies. The seronegative status of Ruwani at the age of five years was a matter of concern. No traces of maternal antibodies could be detected, and an active immunity seemed not to have developed, a~~lthough~~ three episodes of viremia ~~had previously been recorded~~ (Table 3).

LIPS assays using ORF-Q antigens detected fewer seropositive animals than gB-serology. As the subtype EEHV1B had been observed to be shed from the trunk of Omysha (25), it was not surprising that this animal showed the highest reaction among the members of the herd against the ORF-Q-B antigen. In contrast, the other young animals at risk did not have such antibodies nor were they seropositive against ORF-Q antigens A, C, or D. Of the older members of the herd, at least one female elephant (Ceyla) had significant antibody levels against ORF-Q-A, whereas two female elephants (Indi; Chandra) had preexisting antibodies against ORF-Q-C. Interestingly, none of the animals seemed to have significant levels of antibodies against ORF-Q-D antigen, although the isolate from a previous case of EHD in the zoo (Xian, 1999) had been classified to belong to ORF-Q clade D. These results suggested that antibodies against ORF-Q antigens may be short-lived and therefore that young elephants are at risk during an EEHV outbreak.

Thanks to the preventive viremia-screening program, the 2022 outbreak was detected early. However, due to the “protected contact” principles, any treatment option depended on the individual elephant’s decision to interact (30). Although Umesh agreed to intravenous GCV treatment (details to be published elsewhere), the virus burden in the bloodstream increased dramatically (Fig. 3) and six days after viremia was first detected, Umesh died of EHD. The second animal (Omysha) rebounded twice from viremic to aviremic and back but then still succumbed to EHD. Notably, Omysha did not agree to GCV treatment but consented occasionally to take up some oral dose of FCV. Neither GCV nor FCV had a noticeable effect

on the ongoing viremia (Fig. 3). The third animal (Ruwani) had its first positive reading of viremia at the same day as the second animal (Omysha) but had a survival time of three weeks after that, ~~even without consenting to any treatment option~~. Only after its fifth detection of viremia, the virus burden in the blood stream increased with almost every consecutive sample and the animal eventually succumbed to the disease.

Once the outbreak was ongoing, it was of immediate interest to subtype the causative virus more closely. Indeed, initial PCR-sequencing of the E36 gene locus suggested the involvement of a subtype 1A strain rather than a subtype 1B virus as the current disease-causing agent. This conclusion raised new concern about the eight-year-old Omysha, which had been found seropositive for antibodies against the ORF-Q-B antigen but not against other ORF-Q antigens. ~~Moreover~~, the possibility ~~to use her~~ antibody-rich serum for a potential treatment of the other animals at risk ~~was now ruled out~~. As maternal antibodies seem to be able to confer protection, a rapid analysis of the prevailing virus subtype may help to identify quickly a suitable herd mate as serum donor (7).

Subsequently, it was inviting to speculate that one of the previously detected 1A strains, dating back to 1999 and 2003 had re-emerged after long years of latency, which had been the case in Berlin, ~~with Shaina Pali and Ko-Raya (EP23 and EP25) being identical to Plai Kiri from~~ 11 years earlier (1). ~~Yet, a~~ comparison of E54 sequences, including the nucleotide sequences of the Xian (1999) isolate and Umesh revealed significant differences. Thus, the present EEHV1A virus was newly detected throughout this outbreak ruling out the re-emergence of a previously identified latent strain. These analyses during the ongoing outbreak had direct effects on our risk assessment and may have also expanded our treatment options. ~~However, rapid~~ identification of the ORF-Q subtype of the prevailing virus had not been established at the time of the outbreak and ORF-Q serology using subtype C and D antigens were only established in retrospect. Otherwise, Indi and Chandra, both seropositive for ORF-Q-C antibodies (Fig. 2), may have been used as serum donors for antibody transfer.

Evidence that the same new EEHV1A strain infected all three calves

Only once the outbreak of EHD was over, could the entire EEHV1 genomes from all three cases be determined and compared with the result of the initial PCR analysis. Moreover, the sequences of other genomic loci of interest then became available for interpreting the course and origin of the disease as well as the failure of treatment.

Concerning the epidemiological chain, it is important to re-emphasize that all three isolates had the exact same genomic sequence, belonging to the EEHV1A subtype. The E54 vOX2-1 locus was 100% conserved among the three current isolates, which confirmed their common origin, but was significantly different from the Xian (1999) isolate. Furthermore, analysis of the E39 locus of Umesh identified a virus belonging to the ORF-Q-C clade. This finding was very surprising, since no such virus had previously been identified among these animals. Instead, the previous lethal EEHV1A strain ~~responsible for the death of Xian~~, belonged to the ORF-Q-D clade, while serological evidence suggests that the EEHV1B isolate detected in earlier trunk-wash samples from Omysha belonged to the ORF-Q-B clade.

The ORF-P and ORF-Q genes of EEHV1, which arose by duplication and have some residual similarity, are replaced in EEHV3/4 by a single novel gene at the same location and with the same two exons (E39A, ORF-R), but with minimal or no homology to ORF-P or to ORF-Q itself (5). Similarly, EEHV2 and EEHV5 also lack ORF-Q but do have ORF-P. Accordingly, our serology was unable to evaluate the serological status of the animals towards EEHV3/4, although EEHV4 had been detected in the trunk wash sample from Thai the breeding bull and LIPS assays for the specific detection of antibodies against EEHV2 and EEHV3/4 have recently been established by others on the basis of the E34 encoded protein (14).

Unfortunately, the ORF-Q sequence from Aishu, who died from EHD in 2003, was not available. However, the few known parts from the Aishu virus genome, for example the

U48.5 ETk locus, differ considerably from the present sequences. Overall, these sequences confirmed the presence of a new previously undescribed virus strain among these elephants.

Potential antiviral drug targets

Three different EEHV1 encoded enzymes may be involved in the susceptibility or resistance of nucleoside-based antiviral treatment against EEHV: the viral thymidine kinase (U48.5, ETk), the conserved protein kinase (U69, ECPK), and the catalytic unit of the viral DNA polymerase (U38, DNA pol)(16, 40-42).

Identities and differences, respectively, in the coding sequences ~~within these three genes of our three isolates~~ might not only convey information about potential drug-resistance but also about the origin and sequence of transmission. ~~However,~~ since all sequences from our three isolates were identical, the source of infection ~~must be~~ the same for all three animals or the virus must have been transmitted from one case to the other without genomic alteration. ~~Therefore, it is safe to assume~~ that all three animals were infected from the same original shedder.

As its name suggests, the ECPK protein was even more conserved than ETk. Moreover, this enzyme did show some *in vitro* activity, converting GCV and PCV in a dose-dependent manner, ~~thus,~~ making it a prime candidate not only for rendering the virus sensitive to those antiviral drugs but also for a possible emergence of a drug-resistant mutant virus (1, 41, 42). Although a few variations against previous sequences were detected, all the differences had been identified before and mapped outside of the predicted catalytic domain of the enzyme (aa 166 to 306). In the present case, three different aspects have to be considered, which may explain the obvious inability of the antiviral drugs to confer any measurable effect on viral burdens as well as on the clinical outcome of the disease: (1) ECPK and ETk are less active than HSV Tk in converting the prodrugs to their active form, which may negatively affect the desired drug activities. (2) It is possible that the U38 DNA polymerase may be naturally

incompetent to incorporate certain phosphorylated nucleoside analogues into the growing viral DNA. Because of the lack of suitable cell cultures, this issue has not yet been addressed. Still, an interesting variation was observed within the U38 DNA polymerase sequence, where a single mutation (L729I) in all our new isolates opposed to the high conservation among ten randomly selected previous isolates. This seeming mutation mapped to the center of a conserved domain, which had been associated with predicted enzymatic activities, including elongation, DNA-binding, and dNTP binding. (3) Because the treatment efforts ~~strictly~~ depended on the affected animal's ~~consent~~, it is also possible that doses of antiviral drugs did not meet the needed concentrations in the bloodstream.

Presumed long-term quiescence in a herd-mate

Having established that all three animals had been infected most likely from the same source with the same but previously unrecognized virus, its most probable origin can be attributed to an elephant who was born outside of the Zurich zoo. Indeed, ORF-Q serology identified two individuals, Indi and her daughter Chandra, ~~which~~ had been ORF-Q-C-seropositive prior to the outbreak, ~~while all other members of the herd, in particular the newest member Thai and the oldest external animal, Ceyla (born 1975 in Sri Lanka, transferred to the Zurich zoo in 1976), had been seronegative~~. As one of the ORF-Q-C-positive animals Chandra had been born and raised in the zoo, whereas her equally seropositive mother (Indi) had been born 1986 in Myanmar, was ~~then~~ imported to Switzerland in 1988, before being transferred to the Zurich zoo in 1999~~)~~. ~~It is tempting to~~ speculate that Indi has acquired this "novel" virus in her youth but ~~kept it latent or~~ did not spread it until prior to the present outbreak.

Global protein subtype analysis of the intact genome of EEHV1A (Umesh, EP55)

The large DNA genomes of EEHV1 and the six other identified *Proboscivirus* species (EEHV2 to EEHV7) range between 168 and 206-kb in size encoding approximately 120 genes each and have amongst the most variable DNA genomes of all mammalian herpesviruses. This extreme hypervariability is largely confined to within between six and eight non-adjacent protein encoding loci that we refer to as chimeric domains (CD-I to CD-VIII) whose characteristic number, location and size differ somewhat across each species, as well as the gene content within the 4 to 10-kb R2-segment when present. In particular, extensive genomic analyses and comparisons of the hypervariable domains of the most variable EEHV1 proteins show that they often cluster tightly into multiple (3 to 8) distinct cladal patterns that have been designated as subtypes A, B, C, D, E etc. In the special situation of the chimeric EEHV1B genomes the individual gene subtype patterns of the non-adjacent CD-1, CD-II and CD-III variable loci across large sections of the genome are linked, but there is very little linkage observed between the subtype patterns across different EEHV1A strains. Here in the case of the lethal EEHV1A (Umesh, EP55) strain that infected three young Asian elephant calf mates within just a few days of one another, we have determined the complete 175-kb genomic DNA sequence with full annotation of the gene coding content and compared it with nine other selected representative well-studied EEHV1 genomes in supplementary Tables S1 and S2). In particular, we have focused especially on the designated subtypes of all of the 33 most significant hypervariable viral proteins including those contributing to the uniquely R2-segment gene content. ~~The results show that~~ the combined individual protein subtypes obtained for EEHV1A (Umesh) conform to the general expectations of a novel overall mosaic pattern with considerable subtype level divergence across just a scattered subset of hypervariable proteins compared to all other evaluated strains. For Umesh this includes falling into the D-subgroup R2-segment cluster together with two other reference strains (Xian and Ganesh) as defined by the presence of the adjacent E59

(vGPCR10) plus E60 (vIgFam6) and E61 (vIgFam7) genes as their characteristic alternative inserted R2-triplex cassettes. Among the particular ten reference EEHV genomes evaluated in Table S1**,** Umesh is also the only one to encode an E-subtype U51 (vGPCR1) protein as well as the only one to encode a D-subtype U48 (gH) protein. It also shares the C-E-C subtype pattern for the ORF-O, ORF-P, ORF-Q main segment glycoprotein complex only with EEHV1A (Raman).

The fact that EHD evidently involves primary infection in inappropriately or insufficiently immune calves (7, 14, 15) appears fully consistent with the universal finding of the identical species and strain of EEHV infecting two (or more) elephant calves that contracted DNA-positive confirmed EHD nearly simultaneously at the same housing facility. These occurrences have now been documented at least a dozen times previously, including in Germany and multiple times in the United Kingdom and USA plus three times also in Asian range countries India (3, 28), Indonesia (43) and Malaysia (44)~~, although~~ only in Switzerland and Malaysia have the episodes involved as many as three separate lethal EHD cases within young calves with the identical virus strain within a very short time period.

Conclusions

Despite considerable preventive efforts and early detection of EEHV1A viremia, the Zurich zoo lost three young elephants to EHD. The reasons for the failure of the preventive measures may be summarized in three points: (1) Presence of a previously undetected EEHV1A strain among the resident elephants. Possibly, it could be deduced that the new virus re-emerged after almost 40 years of latency from one of the oldest elephants in the zoo. (2) Insufficient or absent strain-specific immunity in the affected animals. (3) Lack of ~~efficient~~ antiviral drugs and protective vaccines, due to the unavailability of appropriate cell cultures and animal models. Having determined the complete genomic DNA sequence of EEHV1A (Umesh, EP55) during the course of this outbreak, we also took the opportunity to make detailed

comparisons of the overall gene content and hypervariable protein subtype clustering patterns with multiple other EEHV1 strains leading to a new level of appreciation of the often very high levels of polymorphic divergence within a subset of the proteins encoded among and across these genomes, especially within the R2-segment. The complexity of those differences with major and minor variations has to be accounted for in the planning of future strategies for prevention and treatment of EHD. Based on the obvious conservation of the three enzymes responsible for sensitivity and resistance to nucleoside analogues, it seems to be more straightforward and feasible to develop efficient antiviral drugs than to understand the role of all those proteins encoded in the chimeric domains and the R2 segment, which may have immunologically decisive effects on conferring protection against EHD upon immunization.

Acknowledgments

We are grateful to the zoos in Zurich, Basel, and Hamburg for their contributions. Equally, Dr. Monika Hilbe and Prof. Dr. Franco Guscetti, Institute of Veterinary Pathology, University of Zurich are greatly acknowledged for providing necropsy tissue. We also thank Prof. Dr. Cornel Fraefel for his constant support of the viremia screening and antibody testing. We thank Dr. Andreina Schramm for sharing her EHV1 positive horse reference sera. We also acknowledge the direct collaboration and assistance of Erin Latimer at the Elephant Herpesvirus Laboratory of the Smithsonian National Zoo and J.-C. Zong of the Viral Oncology Program at Johns Hopkins School of Medicine for much of the unpublished sub-genomic PCR sequencing analysis work carried out on earlier EHD cases collected worldwide that was drawn upon from GenBank accession files for the strain differences analyses provided here in Supplementary Material Tables S1 and S2.

References

1. Long SY, Latimer EM, Hayward GS. Review of Elephant Endotheliotropic Herpesviruses and Acute Hemorrhagic Disease. ILAR J. 2016;56(3):283-96.
2. Hoornweg TE, Schaftenaar W, Maurer G, van den Doel PB, Molenaar FM, Chamouard-Galante A, et al. Elephant Endotheliotropic Herpesvirus Is Omnipresent in Elephants in European Zoos and an Asian Elephant Range Country. Viruses. 2021;13(2).
3. Zachariah A, Zong JC, Long SY, Latimer EM, Heaggans SY, Richman LK, et al. Fatal herpesvirus hemorrhagic disease in wild and orphan asian elephants in southern India. J Wildl Dis. 2013;49(2):381-93.
4. Richman LK, Zong JC, Latimer EM, Lock J, Fleischer RC, Heaggans SY, et al. Elephant endotheliotropic herpesviruses EEHV1A, EEHV1B, and EEHV2 from cases of hemorrhagic disease are highly diverged from other mammalian herpesviruses and may form a new subfamily. J Virol. 2014;88(23):13523-46.
5. Ling PD, Long SY, Zong JC, Heaggans SY, Qin X, Hayward GS. Comparison of the Gene Coding Contents and Other Unusual Features of the GC-Rich and AT-Rich Branch Probosciviruses. mSphere. 2016;1(3).
6. Pellett PE. Trunkloads of viruses. J Virol. 2014;88(23):13520-2.
7. Fuery A, Pursell T, Tan J, Peng R, Burbelo PD, Hayward GS, et al. Lethal Hemorrhagic Disease and Clinical Illness Associated with Elephant Endotheliotropic Herpesvirus 1 Are Caused by Primary Infection: Implications for the Detection of Diagnostic Proteins. J Virol. 2020;94(3).
8. Latimer E, Zong JC, Heaggans SY, Richman LK, Hayward GS. Detection and evaluation of novel herpesviruses in routine and pathological samples from Asian and African elephants: identification of two new probosciviruses (EEHV5 and EEHV6) and two new gammaherpesviruses (EGHV3B and EGHV5). Vet Microbiol. 2011;147(1-2):28-41.
9. Zong JC, Heaggans SY, Long SY, Latimer EM, Nofs SA, Bronson E, et al. Detection of Quiescent Infections with Multiple Elephant Endotheliotropic Herpesviruses (EEHVs), Including EEHV2, EEHV3, EEHV6, and EEHV7, within Lymphoid Lung Nodules or Lung and Spleen Tissue Samples from Five Asymptomatic Adult African Elephants. J Virol. 2015;90(6):3028-43.
10. Zong JC, Latimer EM, Long SY, Richman LK, Heaggans SY, Hayward GS. Comparative genome analysis of four elephant endotheliotropic herpesviruses, EEHV3, EEHV4, EEHV5, and EEHV6, from cases of hemorrhagic disease or viremia. J Virol. 2014;88(23):13547-69.
11. Stanton JJ, Zong JC, Latimer E, Tan J, Herron A, Hayward GS, et al. Detection of pathogenic elephant endotheliotropic herpesvirus in routine trunk washes from healthy adult Asian elephants (*Elephas maximus*) by use of a real-time quantitative polymerase chain reaction assay. Am J Vet Res. 2010;71(8):925-33.
12. Stanton JJ, Zong JC, Eng C, Howard L, Flanagan J, Stevens M, et al. Kinetics of viral loads and genotypic analysis of elephant endotheliotropic herpesvirus-1 infection in captive Asian elephants (*Elephas maximus*). J Zoo Wildl Med. 2013;44(1):42-54.
13. Hoornweg TE, Perera VP, Karunarathne RNS, Schaftenaar W, Mahakapuge TAN, Kalupahana AW, et al. Young elephants in a large herd maintain high levels of elephant endotheliotropic herpesvirus-specific antibodies and do not succumb to fatal haemorrhagic disease. Transbound Emerg Dis. 2022;69(5):e3379-e85.
14. Pursell T, Spencer Clinton JL, Tan J, Peng R, Qin X, Doddapaneni H, et al. Primary Infection May Be an Underlying Factor Contributing to Lethal Hemorrhagic Disease Caused

by Elephant Endotheliotropic Herpesvirus 3 in African Elephants (*Loxodonta africana*). Microbiol Spectr. 2021;9(2):e0098321.

15. Fuery A, Tan J, Peng R, Flanagan JP, Tocidlowski ME, Howard LL, et al. Clinical Infection of Two Captive Asian Elephants (*Elephas Maximus*) with Elephant Endotheliotropic Herpesvirus 1b. J Zoo Wildl Med. 2016;47(1):319-24.

16. Ehlers B, Dural G, Marschall M, Schregel V, Goltz M, Hentschke J. Endotheliotropic elephant herpesvirus, the first betaherpesvirus with a thymidine kinase gene. J Gen Virol. 2006;87(Pt 10):2781-9.

17. Wilkie GS, Davison AJ, Watson M, Kerr K, Sanderson S, Bouts T, et al. Complete genome sequences of elephant endotheliotropic herpesviruses 1A and 1B determined directly from fatal cases. J Virol. 2013;87(12):6700-12.

18. Ling PD, Reid JG, Qin X, Muzny DM, Gibbs R, Petrosino J, et al. Complete Genome Sequence of Elephant Endotheliotropic Herpesvirus 1A. Genome Announc. 2013;1(2):e0010613.

19. Ling PD, Long SY, Fuery A, Peng RS, Heaggans SY, Qin X, et al. Complete Genome Sequence of Elephant Endotheliotropic Herpesvirus 4, the First Example of a GC-Rich Branch Proboscivirus. mSphere. 2016;1(3).

20. Atkins L, Zong JC, Tan J, Mejia A, Heaggans SY, Nofs SA, et al. Elephant endotheliotropic herpesvirus 5, a newly recognized elephant herpesvirus associated with clinical and subclinical infections in captive Asian elephants (*Elephas maximus*). J Zoo Wildl Med. 2013;44(1):136-43.

21. Dastjerdi A, Seilern-Moy K, Darpel K, Steinbach F, Molenaar F. Surviving and fatal Elephant Endotheliotropic Herpesvirus-1A infections in juvenile Asian elephants - lessons learned and recommendations on anti-herpesviral therapy. BMC Vet Res. 2016;12(1):178.

22. Brock AP, Isaza R, Hunter RP, Richman LK, Montali RJ, Schmitt DL, et al. Estimates of the pharmacokinetics of famciclovir and its active metabolite penciclovir in young Asian elephants (*Elephas maximus*). Am J Vet Res. 2012;73(12):1996-2000.

23. Schmitt DL, Hardy DA, Montali RJ, Richman LK, Lindsay WA, Isaza R, et al. Use of famciclovir for the treatment of endotheliotrophic herpesvirus infections in Asian elephants (*Elephas maximus*). J Zoo Wildl Med. 2000;31(4):518-22.

24. Wissink-Argilaga N, Dastjerdi A, Molenaar FM. Using in-House Hematology to Direct Decision-Making in the Successful Treatment and Monitoring of a Clinical and Subsequently Subclinical Case of Elephant Endotheliotropic Herpesvirus 1b. J Zoo Wildl Med. 2019;50(2):498-502.

25. Ackermann M, Hatt JM, Schetle N, Steinmetz H. Identification of shedders of elephant endotheliotropic herpesviruses among Asian elephants (*Elephas maximus*) in Switzerland. PLoS One. 2017;12(5):e0176891.

26. Ackermann M, Hatt J-M. To treat or not to treat? Veterinary Record Case Reports. 2019;7(3):e000847.

27. Ossent P, Guscetti F, Metzler AE, Lang EM, Rubel A, Hauser B. Acute and fatal herpesvirus infection in a young Asian elephant (*Elephas maximus*). Vet Pathol. 1990;27(2):131-3.

28. Zachariah A, Sajesh PK, Santhosh S, Bathrachalam C, Megha M, Pandiyan J, et al. Extended genotypic evaluation and comparison of twenty-two cases of lethal EEHV1 hemorrhagic disease in wild and captive Asian elephants in India. PLoS One. 2018;13(8):e0202438.

29. Oo ZM, Aung YH, Aung TT, San N, Tun ZM, Hayward GS, et al. Elephant Endotheliotropic Herpesvirus Hemorrhagic Disease in Asian Elephant Calves in Logging Camps, Myanmar. Emerg Infect Dis. 2020;26(1):63-9.

30. Rubel A, Zingg R. Kaeng Krachan Elephant Park for Asian elephants (*Elephas maximus Linnaeus*, 1758) at Zurich Zoo. (Kaeng Krachan Elefantenpark fuer Asiatische Elefanten (*Elephas maximus Linnaeus*, 1758) im Zoo Zuerich.) Zoologische Garten. 2015;84(1-2):1-12.

31. Burbelo PD, Ching KH, Klimavicz CM, Iadarola MJ. Antibody profiling by Luciferase Immunoprecipitation Systems (LIPS). J Vis Exp. 2009(32).

32. Work TM, Dagenais J, Balazs GH, Schettle N, Ackermann M. Dynamics of Virus Shedding and In Situ Confirmation of Chelonid Herpesvirus 5 in Hawaiian Green Turtles With Fibropapillomatosis. Vet Pathol. 2015;52(6):1195-201.

33. Kubacki J, Fraefel C, Bachofen C. Implementation of next-generation sequencing for virus identification in veterinary diagnostic laboratories. J Vet Diagn Invest. 2021;33(2):235-47.

34. Kubacki J, Flacio E, Qi W, Guidi V, Tonolla M, Fraefel C. Viral Metagenomic Analysis of Aedes albopictus Mosquitos from Southern Switzerland. Viruses. 2020;12(9).

35. Bolger AM, Lohse M, Usadel B. Trimmomatic: a flexible trimmer for Illumina sequence data. Bioinformatics. 2014;30(15):2114-20.

36. Nurk S, Meleshko D, Korobeynikov A, Pevzner PA. metaSPAdes: a new versatile metagenomic assembler. Genome Res. 2017;27(5):824-34.

37. Altschul SF, Gish W, Miller W, Myers EW, Lipman DJ. Basic local alignment search tool. J Mol Biol. 1990;215(3):403-10.

38. Moraru C, Varsani A, Kropinski AM. VIRIDIC-A Novel Tool to Calculate the Intergenomic Similarities of Prokaryote-Infecting Viruses. Viruses. 2020;12(11).

39. Dominguez G, Dambaugh TR, Stamey FR, Dewhurst S, Inoue N, Pellett PE. Human herpesvirus 6B genome sequence: coding content and comparison with human herpesvirus 6A. J Virol. 1999;73(10):8040-52.

40. Burrel S, Deback C, Agut H, Boutolleau D. Genotypic characterization of UL23 thymidine kinase and UL30 DNA polymerase of clinical isolates of herpes simplex virus: natural polymorphism and mutations associated with resistance to antivirals. Antimicrob Agents Chemother. 2010;54(11):4833-42.

41. Komatsu TE, Pikis A, Naeger LK, Harrington PR. Resistance of human cytomegalovirus to ganciclovir/valganciclovir: a comprehensive review of putative resistance pathways. Antiviral Res. 2014;101:12-25.

42. Topalis D, Gillemot S, Snoeck R, Andrei G. Thymidine kinase and protein kinase in drug-resistant herpesviruses: Heads of a Lernaean Hydra. Drug Resist Updat. 2018;37:1-16.

43. Stremme C, Priadi A, Hayward GS, Zachariah A. Identification of Two Lethal Cases of Elephant Endotheliotropic Herpesvirus Hemorrhagic Disease in Sumatran Elephant Calves in Indonesia. J Zoo Wildl Med. 2021;51(4):985-93.

44. Lee MH, Nathan S, Benedict L, Nagalingam P, Latimer E, Hughes T, et al. The first reported cases of elephant endotheliotropic herpesvirus infectious haemorrhagic disease in Malaysia: case report. Virol J. 2021;18(1):231.

Supporting information

Table S1. EEHV1A (Umesh) Variable Gene Subtype Comparisons.

Table S2. Details about the Ten EEHV1 Genomes Used in Table S1.

Revised Figure1

Click here to access/download;Figure;Revised Fig1_300dpi.tif

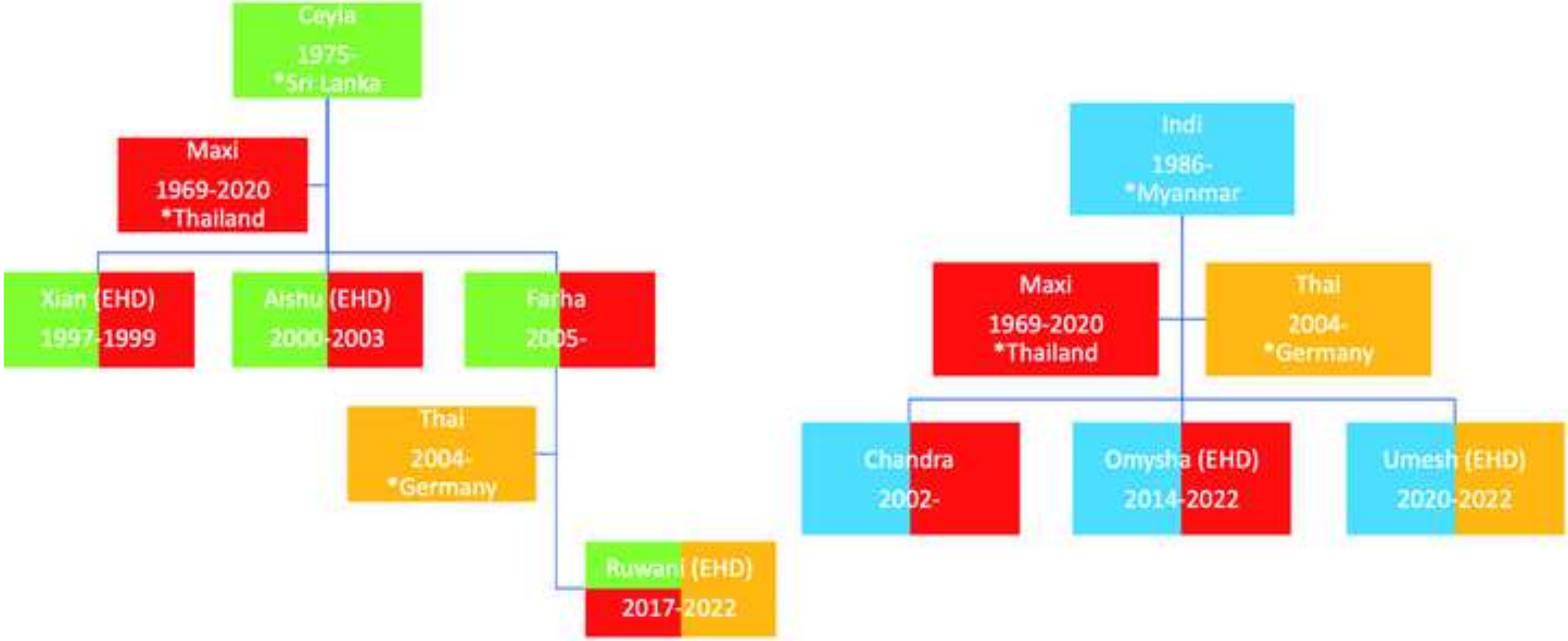

Figure2

Click here to access/download;Figure;Fig2_300.tiff

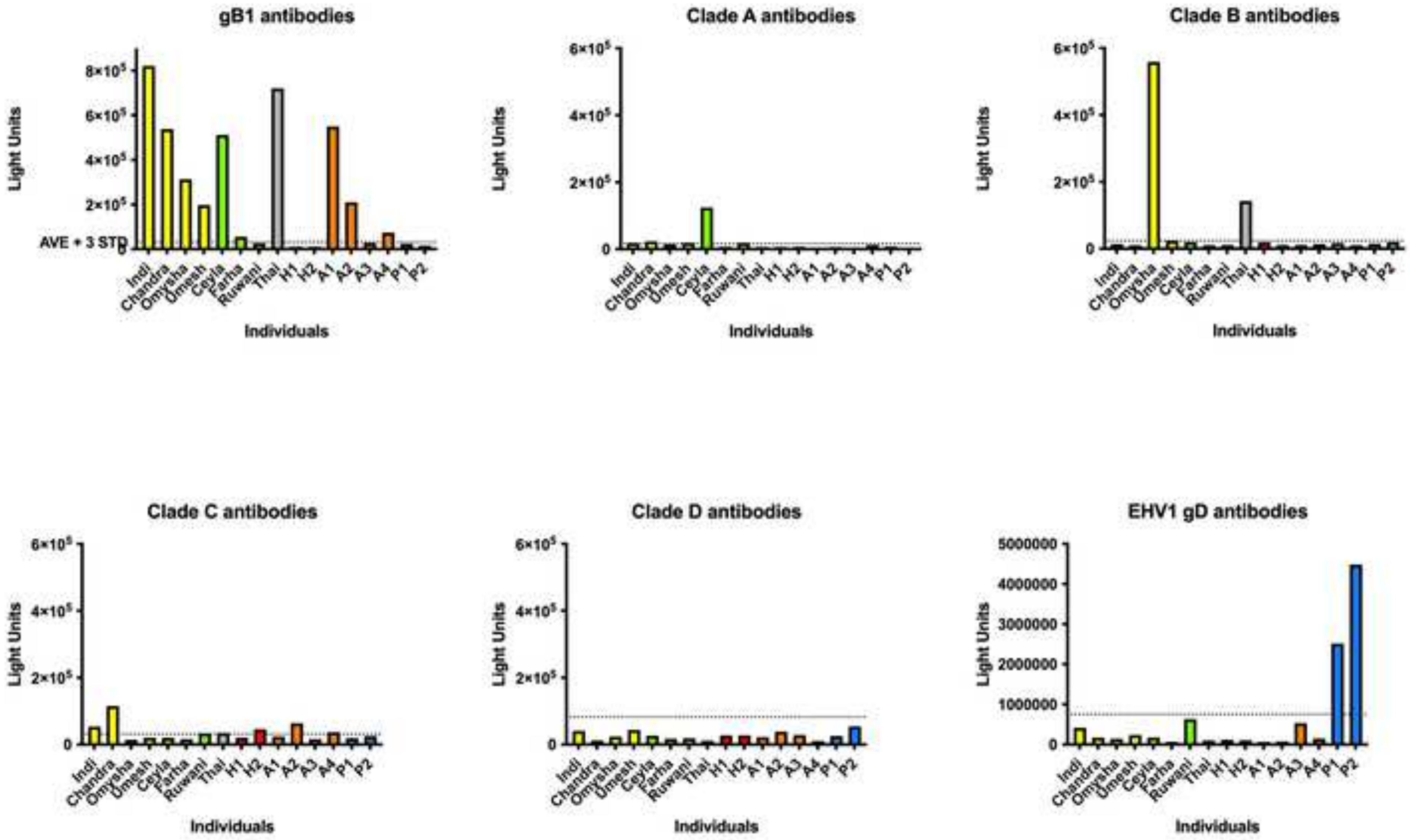

Figure3

Click here to access/download;Figure;Fig3_300.tiff

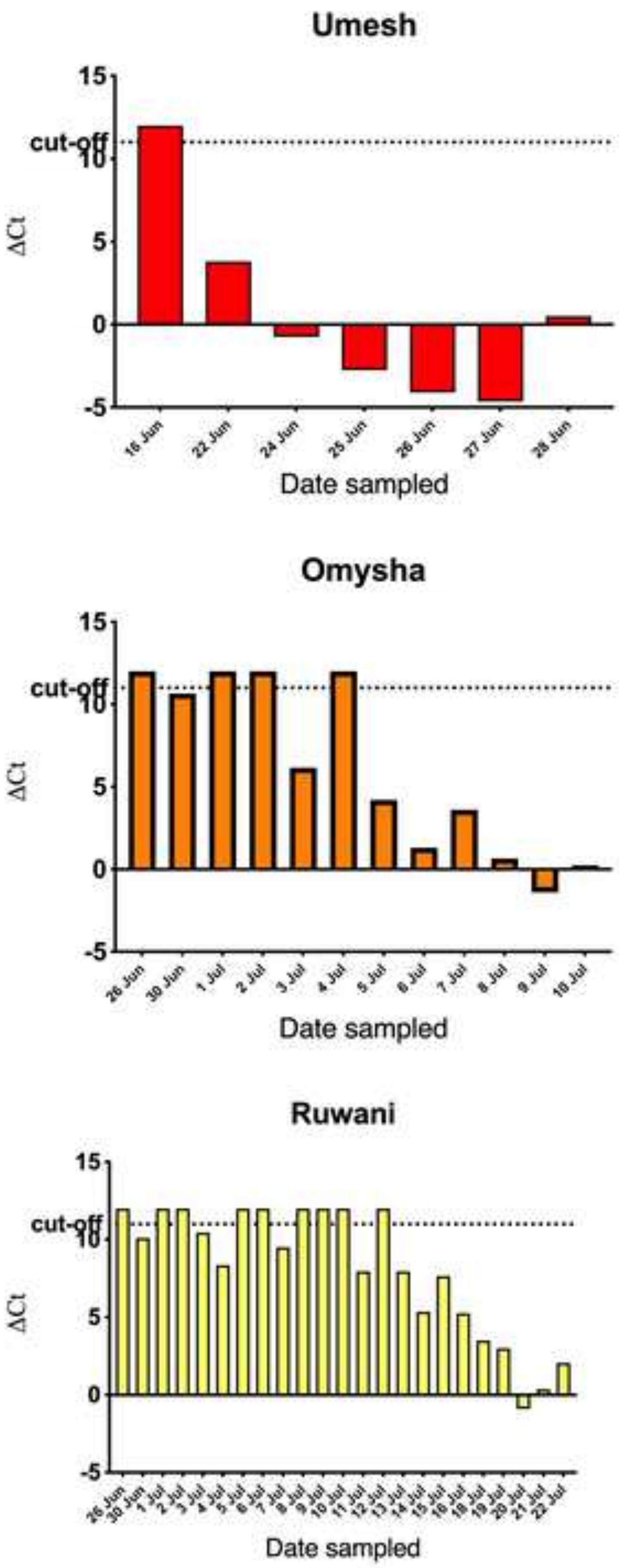

Revised Figure4

Click here to access/download;Figure;Revised Fig4_300dpi.tif

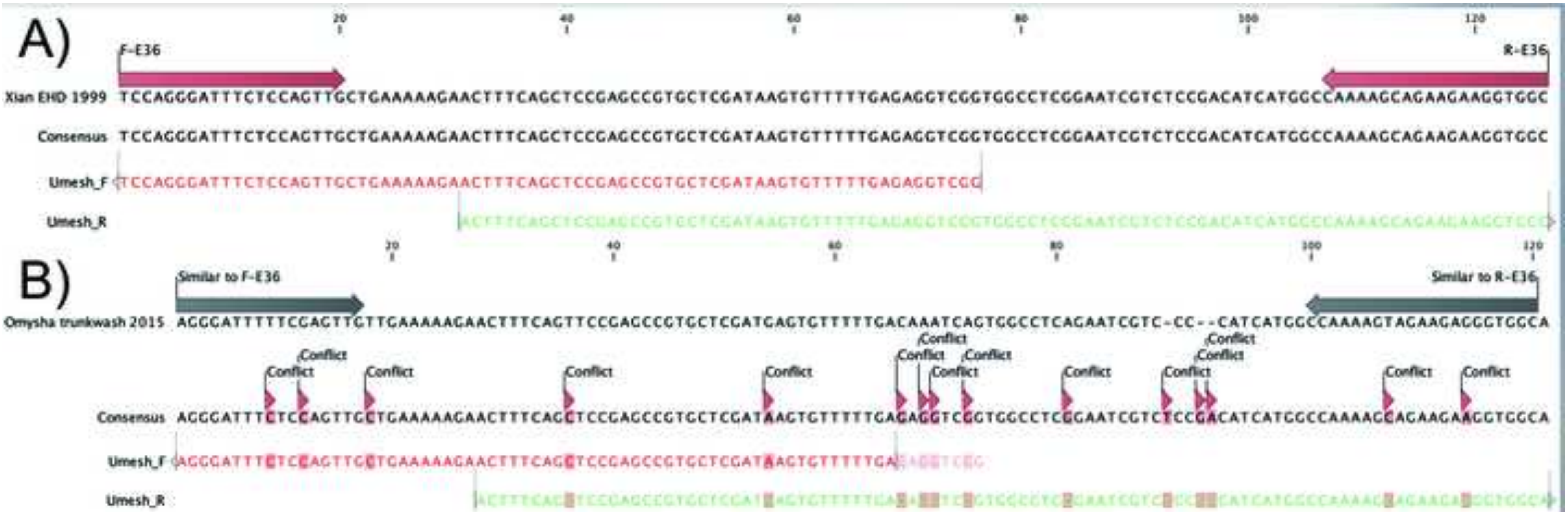

Revised Figure5

Click here to access/download;Figure;Revised Fig5_300dpi.tif

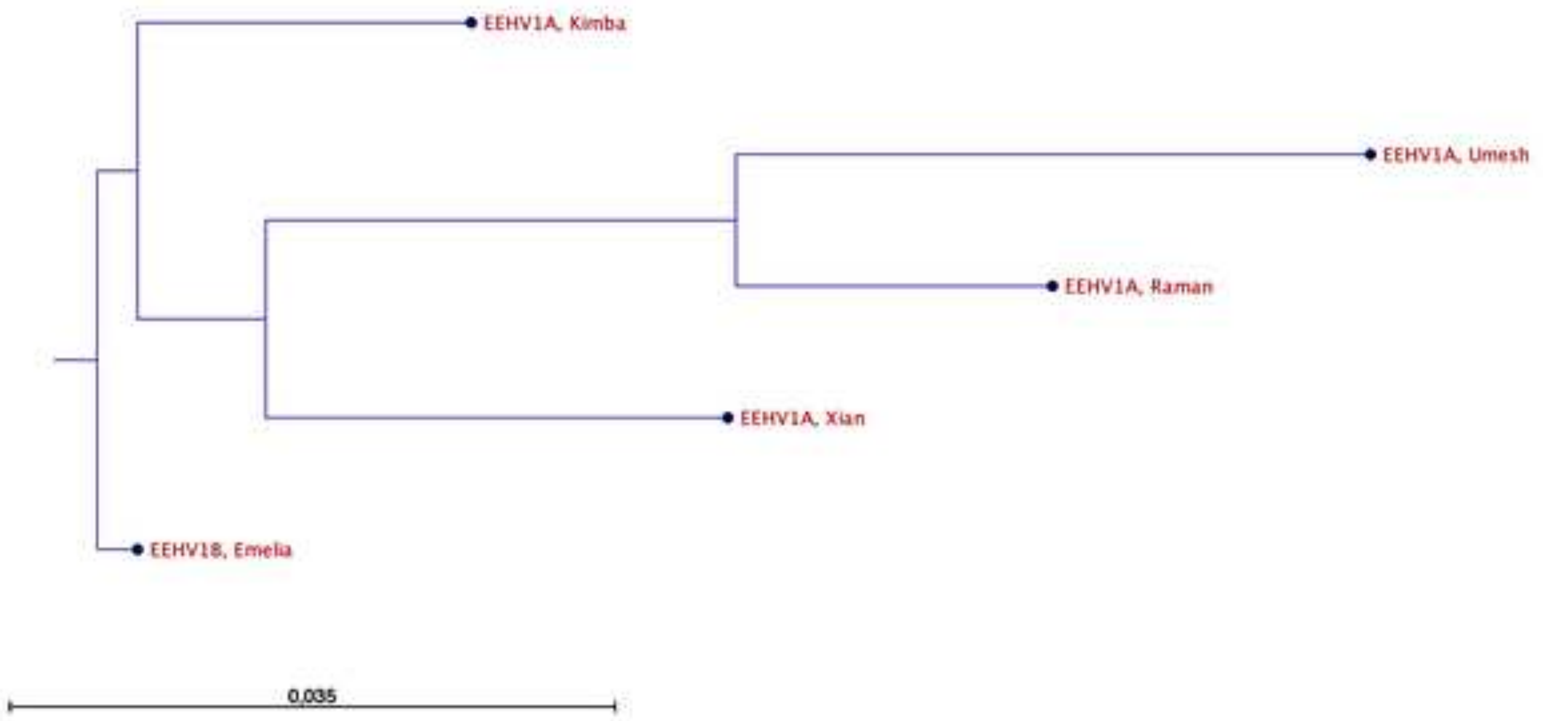

Click here to access/download;Figure;Revised Fig6_300dpi.tiff

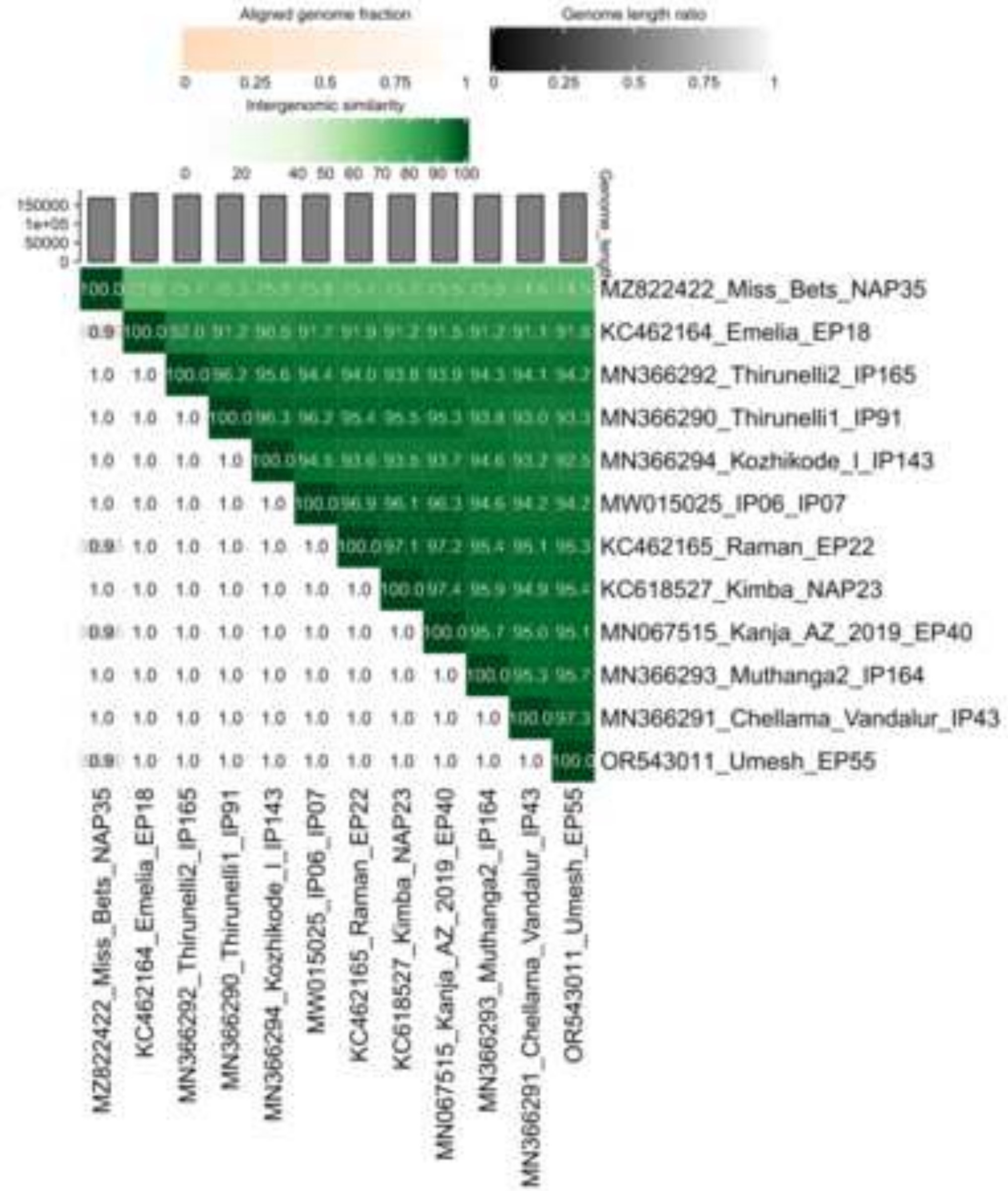

Table S1

Click here to access/download
Supporting Information
Revised TableS1.docx

Table S2

Click here to access/download
Supporting Information
New Table S2.docx

Epidemiological, Serological, and ~~Virological~~Viral Genomic Analysis of an Outbreak of Elephant Hemorrhagic Disease in Switzerland

Mathias Ackermann[1*], Jakub Kubacki[1,2,4], Sarah ~~Heaggans-Ebbeson[3]~~Heaggans[3], Gary S. Hayward[3], Julia Lechmann[1]

[1]Institute of Virology, Vetsuisse Faculty, University of Zurich, 8057 Zurich, Switzerland

[2] Institute of Virology and Immunology, Mittelhäusern, Switzerland

[3] Viral Oncology Program, Johns Hopkins School of Medicine, Baltimore, Maryland, USA

[4] Department of Infectious Diseases and Pathobiology (DIP), Vetsuisse Faculty, University of Bern, Bern, Switzerland.

*Corresponding author

E-mail: mathias.ackermann@uzh.ch

Short Title: Epidemiological, serological, and ~~virological~~viral genomic analysis of EHD in Switzerland

Key words: Elephant hemorrhagic disease (EHD); Elephant endotheliotropic herpesviruses (EEHV); Epidemiology; Serology; genomic sequences, strain variation and subtyping

Formatted: English (United States)

Formatted: English (United States)

Formatted: English (United States)

Formatted: English (United States)

Abstract

Elephant hemorrhagic disease (EHD), caused by several Elephant endotheliotropic herpesviruses (EEHV), represents a frequently lethal syndrome, affecting both captive and free-living elephants. In the summer of 2022, three young Asian elephants (*Elephas maximus*) succumbed to EHD in a zoo in Switzerland, despite of considerable preventive efforts and early detection of EEHV1A viremia. In this communication, we describe the extent of preventive measures in terms of prior virus detection, active survey of viremia, and antibody status. ~~In the course of the outbreak, the causative virus was concomitantly analyzed and eventually fully sequenced and compared to other EEHV types and strains.~~

~~The conclusions from these analyses may be summarized in three points~~The results show that: (1) A previously undetected EEHV1A strain had remained unrecognized among these elephants. Probably, the ~~new~~ virus re-emerged after almost 40 years of latency from one of the oldest elephants in the zoo. (2) While two of the three affected animals had prior immune responses against EEHV1, their strain-specific immunity proved insufficient to prevent EHD. ~~(3) There is an urgent need to develop efficient antiviral drugs and protective vaccines. In particular, ways need to be found to circumvent the present unavailability of appropriate cell cultures and animal models.~~ We have also determined the complete genomic DNA sequence of the EEHV1A strain involved and provide detailed comparisons with the numerous chimeric and hypervariable subtypes of individual genes from multiple other EEHV1 strains revealing a much greater extent of divergence and level of complexity among the encoded proteins than previously described.

Introduction

Elephant hemorrhagic disease (EHD) can be caused by several Elephant endotheliotropic herpesviruses (EEHV, members of the genus *Proboscivirus* within the family of *Orthoherpesviridae*) and represents a frequently lethal syndrome, affecting both captive and free-living elephants (1-4). ~~Pairs of~~Seven distinct but genetically related types (species) of

Formatted: Tab stops: 3.74", Left

EEHV ~~exist in~~that fall into both an AT-rich and GC-rich branch are known to infect either Asian (*Elephas maximus*) ~~and~~or African elephants (*Loxodonta africana*~~), respectively~~) (5, 6). EEHV type 1 (EEHV1), which has been most frequently associated with the occurrence of EHD in both captive and Asian elephants, can be further divided into the chimeric subtypes A (EEHV1A) and B (EEHV1B~~) and~~), as well as into ~~so-called~~cladal ORF-Q ~~clades~~protein subtype clusters encoded by gene E39 (4, 6, 7). ~~Clades~~ Reagents capable of detecting ORF-Q clades A, B, C~~, and~~ or D have been ~~firmly established,~~generated and used successfully at both ~~on~~ the genetic as well as on the ~~serologic~~serological level (7)~~. However,~~, although the existence of at least three further ORF-Q subtypes (clades ~~(~~E1, E2, and F~~ subtypes~~) has now been documented by GenBank entries (E1: QBP78682; E2: QOE74866.1 and QOE74629; F: QBP78717).

The genetic counterpart of EEHV1 in African elephants has been named EEHV6 (8). Among others, EEHV6 has been linked with several cases of EHD in African elephants ~~at~~ in both Europe and the ~~Schönbrunn zoo near Vienna.~~USA (1). The other ~~two pairs of~~five EEHV species are represented by EEHV2, EEHV3 and EEHV7 in African elephants (4, 9, 10) plus EEHV4 and EEHV5 in Asian elephants ~~as well as EEHV3 (in *Loxodonta africana*) and EEHV4 (in *Elephas maximus*)(1, 6, 9)~~(1, 6, 10).

The study of epidemiology, pathogenesis, treatment, and prevention of EEHV-related diseases has been hindered because, as yet, none of the EEHV ~~types,~~ species~~, and~~ or strains can be propagated in conventional cell cultures. Moreover, animal models for EEHV and EHD do not exist because no ~~susceptible animals, except elephants, have ever been detected. Not surprisingly, it took a very long time until full EEHV genomic sequences became available (10, 11).~~ other susceptible species have been identified. Recent partial and full genomic DNA sequence analysis of many EEHV species and strains have revealed large genetic differences between them (4, 9, 10, 11, 18). Furthermore, even within strains of the EEHV1 species itself there are at least 28 viral-encoded proteins that fall into multiple highly-

Formatted: Font color: Red

diverged subtype clusters, including most of the likely envelope glycoproteins such as gB, gH, gL and the three adjacent spliced glycoproteins ORF-O, ORF-P and ORF-Q that are expected to play major roles in immunity and serology.

As with all herpesviruses, the reservoir of EEHV is in latently infected animals, which may intermittently reactivate and shed virus for transmission to herdmates (1, ~~12~~11)~~. All~~. The pathogenesis of EHD is poorly understood. While all known EEHV ~~strains~~species replicate ~~primarily~~ in the respiratory tract ~~but,~~ for ~~poorly understood reasons, can cause~~virus excretion and transmission, viremia may also occur intermittently, which is the major precondition for developing symptomatic EHD (~~13~~12). Another critical parameter for developing EHD is ~~associated to~~ the level of specific anti-EEHV antibodies in the serum. Very young animals are thought to be protected by maternal antibodies ~~under whose cover~~. During this period, subclinical infections may take place ~~to induce~~, which induces active immunological protection against the disease later in ~~adult animals~~life (7, 13, 14~~, 15~~). Accordingly, the highest risk for young animals to succumb to EHD is ~~in the age of between two~~during and ~~eight years and~~right after weaning (2-8y), in association with the waning of maternal antibodies.

Viral genomic sequences have been very useful to determine transmission chains among elephants, which eventually lead to interconnected cases of EHD. For example, the ~~quite variable E54/EE51 locus (encoding vOX2-1) was~~PCR DNA sequences were shown to be identical over at least 3000 bp encompassing the E38/POL, U48/gH-TK), U51/vGPCR1 and U71/gM loci between three cases of EHD ~~in~~at the Berlin~~, which~~ Zoo that occurred 11 years apart: Plai Kiri (European *Proboscivirus* case number 14; EP14) in the year 2000, Shaina Pali (EP23) and Ko-Raya (EP25) in 2011~~. However, the~~ (e.g. GenBank KT705234.1, KT705238.1 and KT705239.1). The knowledge of the ~~present~~current viral sequences ~~are~~is also important for prognosis and treatment: subtype 1A is considered the most virulent EEHV

affecting Asian elephants, whereas subtype 1B is known to frequently cause milder courses of EHD (1, ~~16~~15).

Only recently, the value of serology has also been established as a useful tool in the context of EEHV epidemiology as well as in EHD risk management (2, 7, 13, 14~~, 15~~).

Albeit highly diverged, all known species of EEHV encode thymidine kinase (Tk) as well as conserved protein kinase enzymes (CPK), i.e. U48.5/EE7 encoding the EEHV version of Tk (ETk) and U69 encoding the EEHV version of CPK (ECPK~~,~~), respectively. It has been speculated that these viral enzymes may be capable of phosphorylating nucleoside analogues such as ganciclovir (GCV), aciclovir (ACV), and penciclovir (PCV) or famciclovir (FCV, the PCV-derivate for oral application), which is an essential prerequisite for their potential antiviral activity against EEHV (~~10, 11, 17-19~~16-20). Indeed, nucleoside analogues such as ACV, FCV, and GCV have been used to treat elephants with clinical and virological EHD, but the clinical efficacy of these drugs remains ~~at least~~ debatable (1, ~~20-22~~21-23), although intravenous treatment with GCV coincided at least in one case with decreasing viremia (~~23~~24). However, in the absence of animal models and suitable cell cultures, the true value of treatment against EEHV-infections with nucleoside analogues remains unknown (1).

Despite all these uncertainties, the desire to prevent lethal cases of EHD among young elephants in captivity increased with the growing knowledge about its causative agents and their biological properties. Accordingly, many zoos~~, including ours,~~ initiated surveillance programs to identify prevalent EEHV types and strains, and to monitor their animals at risk for early signs of viremia (~~13, 24~~12, 25). It was expected that typing of the prevalent EEHV species and strains may help to focus on particularly dangerous isolates, while screening for viremia may allow early onset of antiviral treatment in cases of emergency (~~16, 20, 23, 25~~15, 21, 24, 26).

Prior to 2022, three cases of lethal EHD had been recorded among Asian elephants in Switzerland, all of them attributable to EEHV1A, the first in 1988 (partial sequences

deposited in Genbank as EP04, Lohimi: KT705308.1), the second in 1999 (partial sequences in GenBank as EP07, Xian: MH287538.1) and the third in 2003 (EP16, Aishu; no sequence entries in GenBank but unpublished U48.5/EE7 sequences, encoding for ETk, classified the isolate as subtype 1A) (~~24, 26~~25, 27).

In 2014, after many years of successful breeding ~~and, thus, without the need to admitting elephants from outside,~~in a ~~young bull~~closed collection, a 10-year-old male (Thai, see Materials and Methods) was ~~integrated among the elephants in the Zurich zoo~~introduced to the herd, with the aim to replace the aging prior breeding bull (Maxi) and to avoid inbreeding. As a calf had been born in the same year (Omysha), ~~it seemed appropriate to establishing a preventive program~~a protocol was established, which included risk assessment and active surveillance. Initially, EEHV-shedding was assessed, which resulted in the detection of two additional circulating viruses among these elephants, namely an EEHV1B isolate from Omysha as well as an EEHV4 isolate from Thai ~~(24). Accordingly, the young animals were screened on a weekly basis for early detection of viremia by EEHV1 and EEHV4, respectively~~(25). In 2017 and 2020, respectively, two additional elephant calves were born in the zoo, both of which had been fathered by the newly acquired breeding bull. Starting at the age of one year, the young elephants were considered as being at risk to succumbing to EHD. Accordingly, they were included into the preventive program and screened on a weekly basis for viremia by EEHV1 and EEHV4 ~~(25).~~(26). Serology was established "*en route*" as it became available from literature (2, 7, ~~14~~13). Moreover, stocks of ~~antivirals, including~~ GCV and FCV were ~~stored~~acquired for treatment of clinical EHD cases ~~of emergency~~.

In ~~spite of all these precautions, disaster struck in~~ summer 2022, ~~when~~the three ~~of these young~~juvenile elephants from this herd died ~~due to~~of EHD caused by a previously undetected EEHV1A strain.

~~In this communication, we describe and evaluate~~ This paper describes our ~~preventive measures and come close to identify the~~ attempts at identification of the source of ~~the~~this

Formatted: Font: Not Bold

Formatted: Font: Not Bold

outbreak ~~by serology. With only three animals, our data cannot claim statistical significance but in~~ through genomic DNA sequencing of the ~~absence of suitable cell cultures and animal models, they still help us to improve our understanding and strategies against this dreadful disease~~causative virus as well as to evaluate its relationship to other well-characterized EEHV1 strains and for identifying encoded antigenic proteins relevant for developing specific serological assays.

Recent partial and full genomic DNA sequence analysis of many distinct EEHV species and subtypes have revealed large genetic differences between them (4, 10, 17-19). Furthermore, even within strains of the EEHV1 species itself there are at least 28 viral-encoded proteins that fall into multiple highly-diverged subtype clusters, including most of the likely envelope glycoproteins such as gB, gH, gL and the three adjacent spliced glycoproteins ORF-O, ORF-P and ORF-Q that are expected to play major roles in immunity and serology.

The first extensive descriptions of genetic divergence between prototype EEHV1B (Emelia) strain compared to those of the prototype EEHV1A (Kimba and Raman) strains were given in Wilkie et al (10), Ling et al (11) and Richman et al (4). These reports revealed several non-adjacent multigene protein-encoding segments that exhibited often extraordinarily large genetic differences. In particular, Richman et al (4) identified three major diverged multi-gene chimeric domains referred to as CD-I (gB/POL), CD-II (ORF-J/gN/gO/gH/TK) and CD-III (UDG/gL/ORF-O/ORF-P/ORF-Q that map within the central portions of the genome and together encompass part or all of 12 viral genes. These together occupy nearly 15 kb with all but the more conserved POL locus here differing by between 21% and 37% at the protein level between the EEHV1A and EEHV1B versions (4, 5). Despite being widely dispersed these three loci are fully linked and nearly identical in all eight known examples of the EEHV1B diaspora that have been fully evaluated, including EEHV1B (Emelia) and EEHV1B (Kiba) (16) as the prototypes.

Both simplified cartoon genetic maps dividing the prototype EEHV1A (Kimba) genome into L1/L2/L3/C1/C2/R1 and R2 gene-blocks (28) as well as a detailed to-scale gene map of Kimba (5) have been published previously. Overall, comparative analyses at both the single gene or localized sub-genomic level by PCR sequencing, as well as in some cases at the complete genome level have revealed multiple patterns of genetic diversity not only between different AT-rich and GC-rich branch EEHV species, but also among individual isolates (i.e. strains and subtypes) of each EEHV species (4, 5, 10, 19).

Later, when numerous additional strains of EEHV1A were examined in greater detail the CD-II and CD-III regions especially proved instead to display multiple tight cladal clusters of not just subtype A but also many more variants (designated as subtypes C, D, E, F and G etc.) within individual proteins such as U48 (gH) and U82 (gL) as well as E37 (ORF-O), E38 (ORF-P) and E39 (ORF-Q) for example. Moreover, there are also several other subsequently identified hypervariable loci with multiple subtypes mapping across other parts of both the EEHV1A and EEHV1B genomes that are neither linked to each other nor to the classical A versus B diaspora. These encode proteins such as E3(vGPCR6), E5 (vGPCR5), E23/E24/E25 (vOX2-2/3/4) = (CD-VII), E30/31(= CD-VI) and U51(vGPCR1 (= CD-VIII), as well as up to six more hypervariable genes within the R2-segment that also either fall into multiple highly-diverged clusters, or sometimes include being fragmented or having otherwise inactivated versions. Finally, there are also eight alternative groups of inserted triplex R2-segment gene 'cassettes' that have different gene contents and insertion points (see below).

Some details about individual gene subtyping among multiple strains have been presented previously as phylogenetic trees and/or as polymorphism charts. For example, for U39 (gB) and U38 (POL) in Richman et al (4) for U51(vGPCR1) in Long et al (1) for E5 (vGPCR5) and E54 (vOX2-1) in Oo et al (29), in U48.5 (TK) and U48 (gH) (= CD-II) in Zachariah et al (28) and also for E39 (ORF-Q) (= CD-III) in Fuery et al (7). On the other hand, details about the levels of variability and subtype clustering for several other loci e.g. CD-VI (E30/31),

CD-VII (E23/24/25) as well as for E37 (ORF-O) and E38 (ORF-P) and especially about the multiple alternative triplex gene ‘cassettes’ within the R2-segment of EEHV1 strains have not been described previously.

Our analysis of these new cases included a full evaluation of the single novel EEHV1 (Umesh, EP55) strain involved and its patterns of divergence at both the intact genomic and single protein-coding gene levels from other previously encountered or published EEHV1 reference strains.

Materials and Methods

Ethical statement

Viremia samples, tissue samples and sera from elephants were analyzed by order of the contributing zoos. Ethical approval was not sought as the present study only made use of samples that were taken for specific diagnostic purposes, the results of which are described in this manuscript. The samples were taken under routine veterinary care by veterinary staff. The contributing zoos as well as their scientific advisors consider it important to sharing critical medical, husbandry, training, and scientific information with colleagues across the globe.

Elephants

At the time of the outbreak, the herd of Asian elephants (*Elephas maximus*) consisted of eight animals, divided into two family lines and one ~~bull (Thai), the most recent admission to the herd~~breeding bull, who had been introduced from outside in 2014~~ in order to replace~~. Also in 2014, the management of the elephants was changed from “hands on” to “protected contact” principles, which implied that all elephant-human interactions, including all methods of sampling, were determined by the ~~previous~~ individual elephant’s decision to interact (30). The two family lines lived separately but were able to directly interact with each other. Fig 1 gives an overview of the relationships between the individual elephants as well as their life span and origin, if they were born outside of the Zurich zoo. In addition to the present animals, three additional males are indicated in the figure, one (Maxi) being the former breeding bull

(Maxi) (Fig 1). and the other two representing two previous cases of EHD (Xian, EP07 and Aishu, EP16, respectively) in the same zoo.

The first family line, whose founder Ceyla had been born in 1975 in Sri Lanka and transferred to the zoo in 1976, consisted of three individuals, including the founder's daughter Farha and her daughter (Ruwani), born to Farha and Thai. As two of Ceyla's sons (, the new breeding bull. Notably, Xian, EP07 and Aishu, EP16) had previously succumbed to EHD, these two individuals were also integrated into Fig 1.been members of this family line but had been fathered by the previous breeding bull Maxi.

The second family line, whose founder Indi had been born in 1988 in Myanmar and transferred to the zoo in 1999, consisted of four individuals, including the founder's two daughters from Maxi (Chandra and Omysha) and her recent son (Umesh) from Thai.

Thus, all individuals except of the two founders and the bulls had been born in the Zurich zoo.

Fig 1. **Family trees of the elephants relevant for the present study**. Green and yellowblue: founder females with designation and year and country of birth. Red and orange: breeding bulls. Multicolored: color coded offspring with designation, year of birth, and year of death (if applicable). Two prior cases of EHD (Xian, Aishu) are also integrated. EHD in brackets: animal was lost to EHD.

Samples

Viremia samples. For routineweekly viremia screens, one blood droplet (approx. 20 µL) was drawn from the elephant's feet during pedicure by using a small capillary, which then was placed into an Eppendorf tube containing 80 µL nuclease-free water and thoroughly mixed. Due to the "protected contact" principles, the animals chose to occasionally refuse the sampling procedure. Omysha's sampling period started on January 21, 2018, whereas sampling of Ruwani and Umesh started on July 3, 2018 and February 3, 2021, respectively. Routine screening ended with the first detection of serious EEHV1 viremia in Umesh's sample on June 23, 2022.

Historic EDTA blood samples from EHD cases in 2018 (H1 Anjuli and H2 Kanja) were kindly provided by the Hagenbeck zoo in Hamburg, Germany. Cellular fractions were taken as source of DNA extraction for PCR, whereas plasma fractions were used in serology.

Serum samples. Sera from Swiss elephants are routinely collected and archived for health monitoring purposes. Samples from these archives were provided for serological analyses by order of the Basel zoo and in the Zurich zoo, respectively. Samples from the Zurich zoo were collected throughout March 2022. Four sera (A1, A2, A3, A4) from African elephants in Basel zoo were collected throughout January 2022.

Tissue samples. After necropsy of each of the three elephants, 15 mg from each of five tissues (heart, spleen, liver, kidney, lung; kindly provided by Dr. Monika Hilbe, Institute of Veterinary Pathology, University of Zurich) were combined from each individual and minced ~~in a combination of~~with 300 µL of body fluid and 300 µL blood using a ~~TissueLysser~~TissueLyser II (Qiagen).

In addition, a few slices of historic heart and liver tissues from Xian, son to Maxi and Ceyla, who had been lost to EHD in 1999 (partially sequenced genome in GenBank as Xian, EP07), were kindly made available for the present study by Prof. Franco Guscetti, Institute of Veterinary Pathology, University of Zurich.

Real-time PCR detection of EEHV1, EEHV4, and ETNF

~~Per well, 5 µL aliquots from diluted blood droplet samples were used as template for detection of EEHV1, EEHV4 and elephant TNF (ETNF), respectively, by real-time PCR as described previously~~ The previously described real-time PCRs for EEHV1 (targeting U41, the major DNA-binding protein locus) and EEHV4 (targeting U60, the terminase subunit 1 locus), respectively, had been designed for broad range applications, identifying all known subtypes of a particular virus type (~~13, 24~~12, 25)~~. Briefly~~. The elephant TNF-alpha (ETNF) locus was additionally targeted for two purposes, (1) to test for amplifiable elephant DNA in the sample and (2) as a means to quantify the relative amount of viral DNA in the sample

compared to host DNA (12). Briefly, 5 µL aliquots from diluted blood droplet samples were used as template per well. In addition, each 20 µL reaction volume contained 10 µL PrimeTime Gene Expression Master Mix (Integrated DNA Technologies), 0.9 µM of each primer and 200 nM probe, and 5 µL template or nuclease-free water as negative control. Samples were tested in duplicate on a QuantStudio 3 Real-Time PCR System (Applied Biosystems) using the following cycling conditions: 2 min at 50°C, 10 min at 95°C, 40 cycles of 15 sec at 95°C and 1 min at 60°C.

A test was considered valid if ETNF was detected at a Ct value of 39 or lower and if the negative controls for each assay did not yield a Ct value. To compensate for sample variation in volume and elephant cells, the Ct value of ETNF was used as an approximate measure for the number of elephant cells within the sample~~ and, in positive cases~~. Although, the virus target is present at one copy per virus genome, and the host target is present at two copies per diploid genome, the ETNF value is proportional to the cell number in the assay and, thus, can be used to measure the proportion of viral genomes and cellular genomes, respectively. Therefore, the Ct value of the ETNF signal was subtracted from the Ct value for EEHV to get a ΔCt value ($\Delta Ct = Ct_{EEHV}\text{-}Ct_{ETNF}$). ~~Assuming similar test efficiencies, a ΔCt value of zero signified approximately equal amounts of ETNF and EEHV templates, whereas a positive ΔCt value indicated less than one EEHV genome per cell.~~ Accordingly, the ΔCt values grew on the negative side with increasing numbers of EEHV genomes per cell.

Conventional PCRs

~~Two loci on the EEHV1 genome (E36/U79 and E54/EE51 vOX2-1) were targeted by~~Sequence analysis of conventional PCR ~~to characterize~~products can provide rapid information about the identity of the current ~~viral genotype~~virus strain, even very early during ~~the~~an outbreak~~ (Table 1). The PCR conditions were as described~~. In a first round, we used a previously established PCR targeting the E36/U79 locus to discriminate between EEHV1A and EEHV1B strains ~~(24). Briefly, the~~(25). In a second round, we targeted the E54/EE51

vOX2-1 locus, which is expected to remain stable in separate isolates from the same source, while isolates from differing sources may differ from each other by diverging up to 15% at both the nucleotide and the aa level (28). The oligonucleotides used in these PCRs are listed in Table 1. The PCRs were run in a volume of 20 µL, comprising 5 µL template, 10 nM dNTP, 5x Phusion GC buffer, 2 U of Phusion High-Fidelity DNA Polymerase (Thermo Scientific) and each primer at a concentration of 0.5 µM (Table 1). After an initial denaturation for 2 min at 98°C, the samples were cycled 39 times at 98°C for 10 sec, 58.4°C for 20 sec, and 72°C for 7 sec. The runs were completed with 10 min at 72°C before cooling to 10°C. The PCR products were evaluated for their size by agarose gel electrophoresis before being extracted from the gel for cycle sequencing by a commercial company (Microsynth, Balgach, Switzerland).

Table 1. Oligonucleotides used for conventional PCR

Target	Oligo	5' to 3' sequences	Amplicon size
E36/U79	F-E36<br>R-E36	TCC AGG GAT TTC TCC AGT TG<br>GCC ACC TTC TTC TGC TTT TG	126 bp
E54/EE51	AttB1_vOX_R1	GGGGACAAGTTTGTACAAAAAAGCAGGCT<br>ATGCT[T/**C**]CAGAGAAAGTACAGGTAC[a]	930 bp
	AttB2_vOX_L1	GGGGACCACTTTGTACAAGAAAGCTGGGT<br>GTGTTGCCG[T/**C**]CACGATGC**C**TTCTACG[b]	

The primers targeting the vOX2-1 locus, encoded by E54/EE51, were elongated by att-recombination sequences (grey) for facilitated cloning and sequencing after PCR amplification. Moreover, an equimolar mixture of two different forward (R1 with wobbling at position 6)[a] and reverse (L1 with wobbling at position 10)[b] primers was used to compensate for published sequence variations at this site.

LIPS assays

LIPS antigens. The selection and production of LIPS antigens was modified from a previously published protocol (7). Antigen for LIPS serology was harvested from transfected COS7 cells, using synthetic and codon-optimized DNA ~~provided by (~~GenScript Europe ~~(The,~~ the Netherlands). For this purpose, the published amino acid sequences of E39 ~~(~~clades A (Kimba), B (Emelia), C (Raman), and D (Daizy~~))~~) encoding ORF-Q (7) as well as of the gB glycoprotein (EEHV1A, Kimba~~)~~, (18)) were reverse translated, codon-optimized for expression in human cells, and cloned under the control of the cmvIE promoter into the pcDNA3.1(+) vector. Moreover, each construct was fused with the following sequence of C-

terminal tags: V5-epitope, nanoLuciferase (nLuc), and 7-his. Except for ORF-Q clade D~~-E39~~, each construct possessed its native signal peptide, which was included into the gene synthesis design. As clade D~~-E39~~ was not predicted to possess a functional signal sequence but two short transmembrane regions close to the N-terminus, the molecule was shortened to start only at its original amino acid No. 60, which was preceded by the clade A signal peptide. Expression of the desired antigens following transfection was verified by measuring nLuc activity (relative light units, RLU) in the cell culture supernatants as well as by detecting the corresponding proteins in cell lysates after standard Western immunoblotting using a commercial anti-V5 monoclonal antibody (SV5-Pk1 from Invitrogen).

LIPS assay. The protocol for the LIPS assay was adapted from a previously published overview article ~~(27). A commercial kit (Immunoprecipitation Kit, Abcam~~(31). A commercial kit (Immunoprecipitation Kit, Abcam, Cambridge, UK), which provided buffers for protein extraction and solubilization as well as Protein A/G sepharose beads and washing buffers, was used according to the supplier's instructions. Briefly, the secreted antigens were harvested from the cell culture supernatants and mixed with non-denaturing solubilization buffer (NDSB) and protease inhibitors before non-soluble proteins were removed by centrifugation. Prior to the assay, the nLuc activity of each antigen was adjusted to comprise 10^7 RLU per second (RLU/s) in a volume of 50 µL (NanoGlow, Promega, Madison, Wisconsin, USA).

The sera were prediluted with phosphate buffered saline (PBS) to 1:100 in a polystyrene microtiter plate (Nunc F96 MicroWell flat bottom) and mixed well by shaking. ~~Prediluted serum stocks~~These were kept in these plates at -20°C for a maximum of three weeks.

A second polystyrene plate was used for the actual assay. First, 40 µL of NDSB were pipetted into each well before 10 µL of prediluted sera and 50 µL of freshly diluted nLuc-antigen were added. The mixture was incubated ~~with~~by shaking (300 rpm) for one hour at ambient

temperature. Meanwhile, a slurry comprising 30% sepharose A/G beads was prepared in PBS and 5 µL were added to each serum sample to incubate for one more hour with shaking. Eventually, all samples were transferred to a filter plate (~~Merck~~ MultiScreen, Merck, Zug, Switzerland) and washed on a vacuum manifold. Each well was washed ~~8~~eight times with 100 µL of wash buffer, followed by two times washing with PBS. Following the last wash, the vacuum was turned off. The filter plate was removed and blotted dry using a stack of filter paper making sure to remove moisture on the top and bottom of the plate. Eventually, 50 µL of NanoGlow substrate (Promega) was added to each well before measuring in a luminometer (MicroLumat Plus, LB 96 V, up-version 2.0; software WinGlow v. 1.25.000003, Berthold Technologies, Bad Wilbad, DE). ~~Two~~Since horses are expected to be resistant to EEHV-infections and, therefore, free of antibodies against any EEHV, two horse sera (P1 and P2), both known to have antibodies against equine herpesvirus 1 (EHV-1) were used as controls to determine the negative cut-off values of each test. As no true EEHV-negative elephant sera are available, the cut-off decisions were based on a previous publication on marine turtle serology, where a similar problem exists (32).

Sample Preparation and DNA Sequencing

~~Eventually,~~ DNA was extracted from necropsy samples for whole genome sequencing. A previously established viral particle enrichment protocol was used to increase the recovery of viral genetic material (~~28~~33). Then, enrichment of viral nucleic acids was followed by reverse transcription for any RNA viruses (to exclude other causes of death) and sequence-independent single primer amplification for DNA. ~~Sheared~~The nucleic acids were sheared to 500 bp length using the E220 Focused-ultrasonicator (Covaris, Woburn, MA, USA) and prepared with the NEBNext Ultra II DNA Library Prep Kit for Illumina (New England Biolabs, Ipswich, MA, USA) according to the manual. Sequencing libraries were sequenced at the Functional Genomics Center Zurich (FGCZ) in a paired-end 2 × 150 bp, SP flow cell

sequencing run using the NovaSeq 6000 (Illumina, San Diego, CA, USA). The PhiX Control v3 Library (Illumina, San Diego, CA, USA) was added as the control.

Data analysis

Sequenced reads were analyzed using the *de novo* assembly pipeline described previously (~~29~~34). Illumina sequencing adapters and low-quality sequencing ends were trimmed using Trimmomatic (v. 0.39) (~~30~~35). ~~Second~~Subsequently, trimmed reads with average quality above 20 and longer than 40 nt were further cleaned up by trimming away the SISPA primers using cutadapt (v. 3.2). The quality-checked reads were then assembled using metaspades (v. ~~3.12.0) (31). Assembled de novo contigs were compared to the NCBI non-redundant database (nt) using blastn (v. 2.10.1+) and annotated using the best blastn hits.~~3.12.0) (36). Assembled *de novo* contigs were compared to the National Center for Biotechnology Information (NCBI) non-redundant database (nt) using BLAST-N (v. 2.10.1+) and annotated using the best BLAST-N hits. Quality-checked reads were mapped back to the assembled sequences using bwa (v0.7.17) mem ~~(32).~~(37). In addition, contigs from EEHVs were aligned in a metagenomic pipeline of the SeqMan NGen v.17 (DNAStar, Lasergene, Madison, WI, USA) to all available viral genomes of other EEHVs downloaded from the NCBI database (08.2022) and visualized in the SeqMan Ultra (DNAStar, Lasergene, Madison, WI, USA). To determine genome similarity values between our assembled strain and several other available complete EEHV1A strains, as well as EEHV1B (Emelia), and EEHV6 (North American Proboscivirus case number 35; NAP35) we performed ~~BLASTN~~BLAST-N analysis using the VIRIDIC tool (settings: '-word_size 7 -reward 2 -penalty -3 -gapopen 5 -gapextend 2') ~~(Moraru et al., 2020).~~ (38).

Formatted: Font: Italic

Annotation of EEHV Genomes

Typical EEHV genomes comprise about 120 genes (1, 11, 12, 19). Almost 40 of these are also found as conserved but characteristically diverged versions within the majority of other

mammalian herpesvirus sub-families and are referred to as “core” genes with an applied U number nomenclature system derived from that used originally for human HHV6 genome analysis (39).

The remaining 80 or so *Proboscivirus*-specific protein encoding genes that have not been found in any other herpesviruses have been given a sequential unique E number nomenclature system based on that originally applied to the prototype EEHV1A (Kimba) genome (4, 5, 12). Universally agreed upon three-letter abbreviations were used to name proteins with previously available functional or homology data from other well-studies herpesvirus species. Otherwise, we used a combination of either an extended ORF-A to ORF-S system as originally suggested by Ehlers et al (16) or just left them with the same E number as for the designated gene name where little else is known for a novel *Proboscivirus*-specific protein. In terms of detailed gene-by-gene analyses, there are two alternative published physical genetic maps of prototype EEHV1A genomes that Umesh can be compared to with that of EEHV1A (Raman) from Wilkie et al (17) being drawn in the opposite orientation and using a different (EE number) gene nomenclature system. Furthermore, the Umesh genome described here and most other intact annotated AT-rich branch genomes of EEHV1, EEHV2A, EEHV5B and EEHV6 strains in GenBank have been given the same orientation and E number nomenclature as that used for Kimba (18), except for having their R2-segments switched to the extreme left-hand-side compared to our original inadvertent placement on the right-hand-side within a linearized version of the circular EEHV1A (Kimba) genome after the actual packaging cleavage site in Raman DNA was determined (17). Curiously, none of the GC-rich branch *Probosciviruses* EEHV3A, EEHV3B nor EEHV4A or EEHV4B complete genomes even have an R2-segment (19). Note that based on the precedent from Fuery et al (7) of using the term “clades” rather than “subtypes” for multiple E39 (ORF-Q) gene variants, as well as for their corresponding ORF-Q antigens and antibodies, we have used that descriptor throughout

this paper, but only for this particular locus because of its now proven usefulness for differential detection of particular EEHV1 strains in serological and immune response assays.

Sequence sources

~~Publicly available sequences from NCBI were used for comparison of our data. The most relevant cases are listed in Table 2.~~

~~**Table 2. List of EEHV**~~ Publicly available nucleotide as well as protein sequences from NCBI were used for comparative analysis of our data. For the full genomic nucleotide sequences of reference strains used, their case numbers and names as well as origins and year of collection of the individual infected elephants and sources to reference sequence information are listed in Table 2**.** Otherwise, the sources of selected relevant protein and nucleotide sequences used from incomplete sub-genomic loci are provided in the corresponding text passages or figures. Similarly, for those EEHV cases associated with supplementary Table S1 that data for both complete genomes and relevant sub-genomic PCR DNA sequence file accession numbers is provided in Table S2.

Table 2. Used EEHV full genomic sequences

Virus	~~Case~~Case[1]	Individual	Origin	Year	~~ORF-Q~~	Source
EEHV1A	NAP23	Kimba	USA	2004	~~A~~	KC618527.1
~~EEHV1A~~	~~NAP72~~	~~Daizy~~	~~USA~~	~~2015~~	~~D~~	~~KX759150.2~~
EEHV1A	EP24	Raman	UK	2009	~~C~~	KC462165
EEHV1A	EP55	Umesh	Switzerland	2022		OR543011
EEHV1B	EP18	Emelia	UK	2006	~~B~~	KC462164
~~EEHV1A~~	~~EP14~~	~~Plai Kiri~~	~~Germany~~	~~2014~~	~~C~~	~~MH287551.2~~
~~EEHV1A~~	~~EP07~~	~~Xian~~	~~Switzerland~~	~~1997~~	~~D~~	~~MH287543.2~~
						~~MH287538.1~~
EEHV6	NAP35	Miss Bets1	USA	2009	~~6~~	MZ822422

[1]NAP#, North American *Proboscivirus* case number; EP#, European *Proboscivirus* case number

Formatted: Line spacing: Double

Deleted Cells

Formatted: Centered

Formatted: Centered

Formatted: Centered

Deleted Cells

Formatted: Centered

Formatted: Centered

Deleted Cells

Formatted: Centered

Formatted: Centered

Deleted Cells

Formatted: Centered

Results

Weekly screening for EEHV-viremia

~~Starting from 21~~Between January 2018 and June 2022, capillary blood of the juvenile elephants was ~~taken~~analyzed on a weekly basis ~~and analyzed~~ by TaqMan real-time PCR for the presence of EEHV1 and EEHV4 DNA, respectively. ~~Initially, only animal Omysha was monitored but the younger animals were included once they tolerated the sampling procedure.~~ The results of this monitoring program are summarized in Table 3. The average Ct value for ETNF amounted to 31.5 with a standard deviation of 3.1. Although rarely and at low titers~~,~~ (average Ct values ranging between 35.3 and 40), transient (never on subsequent testings) and subclinical viremia by EEHV1 was detected in two of the three monitored animals. Notably, EEHV4 DNA was never detected throughout the screening period.

Table 3. Results of TaqMan real-time PCR targeting EEHV1, EEHV4 and ETNF.

**~~Identification~~Animal**	**Screening**	**~~Detection of EEHV~~Tests (% valid)**	**EEHV1**	**EEHV4**
**Omysha**	231 weeks	211 (98.2%)	~~4~~6 times ~~EEHV1; ACt>4~~pos	Never detected
**Ruwani**	209 weeks	189 (99.5%)	3 times ~~EEHV1; ACt>4~~pos	Never detected
**Umesh**	60 weeks	59 (98.3%)	Never detected	Never detected

EEHV serology using LIPS assay

~~A~~Based on the negative cut-off value determined by horse sera, a LIPS assay using the conserved glycoprotein B of EEHV1 (gB1) as antigen, revealed that all adult individuals possessed antibodies against gB1 (Fig 2A). Interestingly, even adult African elephants reacted positively in this assay, which can be attributed to the close antigenic relationship (89% amino acid identity) of gB1 and EEHV6-gB (gB6). ~~In contrast, horses, which are expected to be resistant to EEHV-infections remained seronegative. Similarly~~In contrast, the sera of two

Formatted: Font: Not Bold

Inserted Cells

Inserted Cells

Inserted Cells

young elephants from Germany who previously had succumbed to EHD did not reveal such antibodies. Interestingly, two of Zurich elephants at risk (Omysha; Umesh) had detectable levels of anti-gB1 antibodies in their sera, whereas the third animal (Ruwani) appeared seronegative.

Next, the elephant sera were analyzed against ORF-Q clade A, B, C, and D antigens (Fig 2B-E). In general, the antibody levels against the ORF-Q antigens were considerably lower than against the conserved glycoprotein B. However, at least one individual (Indi) reacted clearly above background levels against ORF-Q clade A antigen, whereas two individuals (Omysha and Thai) were clearly seropositive against ORF-Q clade B antigen. Moreover, ~~at least~~ two of the individuals at risk (Indi, Chandra) reacted positively against ORF-Q clade C antigen. Interestingly, two of our control sera (one of the German individuals who had died from EHD and one African elephant) also reacted above the background level. Although at least two individuals (Indi, Ceyla) had been present during a previous outbreak of an ORF-Q clade D virus, none of the individuals at risk was presently deemed seropositive against ORF-Q clade D antigen.

As all negative cut-off values were calculated by using horse sera, it seemed sensible to analyze the same panel of sera against an antigen (glycoprotein D, gD), which is suitable to detect antibodies against equine alphaherpesvirus 1 (EHV-1), a highly prevalent pathogen of horses. Indeed, both horse sera tested reacted positively, whereas all elephant sera had no detectable antibodies against the equine viral antigen (Fig 2F).

Together, these serological analyses confirmed both the presence and replication of various EEHV1 subtypes among the animals at risk.

Fig 2. **Prior antibody responses against EEHV1 antigens.** LIPS assays were performed to detect antibodies against various EEHV1 antigens in the sera of elephants and horses. In each panel, the individual elephants are lined up on the x-axis, whereas the reaction of their sera is given in relative light units (RLU) on the y-axis. Yellow bars: Members of the Indi family; green bars: members of the Ceyla family; grey bar: Thai, most recent outside acquisition to the herd, introduced in 2014; red bars: sera from previous EHD cases in the Hagenbeck zoo, Hamburg, Germany; mandarin-colored bars: African elephants from the Basel zoo,

Switzerland; blue bars: horse sera, which were used to determine the negative cut-off value of the test.

Panel A. Antibody responses against the conserved glycoprotein gB1; **Panel B**: antibodies against ORF-Q clade A; **Panel C**: antibodies against ORF-Q clade B; **Panel D**: antibodies against ORF-Q clade C; **Panel E**: antibodies against ORF-Q clade D; **Panel F**: antibodies against equine alphaherpesvirus 1 gD.

Viremia upon EHD

On~~ Thursday,~~ June 16, 2022, all three of the monitored animals tested negative for both EEHV1 and EEHV4, but one week later, on June 23, Umesh tested EEHV1-positive with a ΔCt value of +3.8. Samples from the two following days confirmed its increasingly viremic status with ΔCt values of -0.75 and -2.75, respectively~~. Eventually, the~~, upon which intravenous GCV treatment was initiated. Nevertheless, the ΔCt value of the same animal dropped to -4.1 and to -4.65 before coming back to +0.5 at the day of its death due to EHD, five days after initial detection of viremia (Fig 3). The other two animals remained EEHV1-negative until slight viremia was first detected on June 26. Both went back to negative in their next two samples. On July 3, Omysha's ΔCt value was +6.15 and, after a brief recovery dropped to -1.4 before the animal died 10 days after initial detection of viremia. Beginning from June 30, Ruwani went forth and back with short periods of viremia. Eventually, the ΔCt also dropped to negative values and this animal was also lost to EHD within four weeks (Fig 3).

Fig 3. ~~Relative~~Individual EEHV1 loads~~ in three animals~~ before and throughout development of EHD. Delta Ct values are shown on the y-axis, whereas the date of blood sampling is given on the x-axis.

Analysis of the causative EEHV

Sequencing of the E36/U79 locus

~~The above used TaqMan real-time PCR for detection of EEHV1 DNA targets the U41 gene (encoding the major DNA-binding protein; MDBP) and does not discriminate between the EEHV1 subtypes A or B. Being aware of previous circulation of both subtypes among the elephants at Zurich zoo (24), a potential antiserum treatment was considered. Therefore, it was of immediate relevance to identify the subtype of the present virus. A conventional PCR~~

~~targeting the E36/U79 locus was therefore initiated and the resulting PCR product was sequenced. As shown in Fig 4, the resulting sequence obtained from an early sample of animal Umesh was 100% identical to Xian's previous EEHV1A isolate, whereas it showed (in between the primers) 11 different nucleotides in comparison to Omysha's previous EEHV1B isolate.~~

~~**Fig 4. Sequence alignments**. Alignment of the present E36/U79 sequence (Umesh isolate~~Having established an ongoing viremia with EEHV1, a conventional PCR targeting the E36/U79 locus showed (Fig. 4) that the resulting sequence obtained from an early Umesh sample was 100% identical to Xian's previous EEHV1A isolate. In contrast, 11 different nucleotides in comparison to Omysha's previous EEHV1B isolate were observed, which identified the current isolate clearly as EEHV1A.

Fig 4. Sequence alignments. Alignment of the present E36/U79 sequence (Umesh, EP55) against previous ones. (A) Comparison against the 1A subtype (Xian, EP07~~ isolate~~). (B) Comparison against the 1B subtype (trunk wash from Omysha).

~~Analysis~~Initial analysis of the E54/EE51 vOX2-1 locus

~~As the vOX2-1 locus has~~Having established that Umesh had been ~~reported to be highly variable among strains of EEHV1 (up to 15%) but remains identical in epidemiologically linked cases of EHD (33),~~ affected by an EEHV1A strain and knowing Xian's E54/EE51 vOX2-1 sequences from 1999 (GenBank: MH287538), it was of immediate interest to determine the identity level among the two viruses. Therefore, we amplified ~~an 872 bp~~ DNA ~~fragment~~ from ~~a~~Umesh's blood sample ~~of Omysha~~ by conventional PCR~~.~~ targeting the E54 locus. Sanger sequencing of the PCR product yielded 840 nucleotides, which were compared against ~~the~~Xian's previously determined vOX2-1 sequence ~~obtained from EP07, who died from EHD in the Zurich zoo in 1999~~ as well as against an American (Kimba, 2004) and a European EEHV1A strain (Raman, 2009) and rooted against the subtype 1B prototype (Emelia,2006~~)~~) (~~10, 11~~17, 18). On the nucleotide level, the vOX2-1 sequences of the present Umesh isolate (2022) shared only 92.34 – 93.84% identity with any of the compared

sequences, corresponding to a minimum of 54 to a maximum of 67 ~~nucleotides~~nucleotide polymorphisms per sequence. The closest relationship was observed with the European 1A prototype (Raman), the least with the American 1A prototype (Kimba) (Fig 5). ~~The~~Overall, the present Umesh isolate ~~(EP55) branched with an unrelated European isolate, thus~~differed clearly ~~differing~~ from the previous ~~EHD cases in the zoo~~Xian isolate.

Fig 5. vOX2-1 phylogenetic tree. A phylogenetic tree was obtained from the alignment of the nucleotide sequences of the vOX2-1 locus (E54/EE51) from the American prototype of EEHV1A (~~NAP23~~Kimba), the European EEHV1A prototype (~~EP24~~Raman), the European prototype of EEHV1B (~~EP18~~Emelia) in comparison to the corresponding sequences of the present case (Umesh~~, EP55~~) and ~~a previous~~the 1999 case of EHD in the Zurich zoo (Xian~~, 1999, EP07). Each strain is identified by its case number followed by its GenBank accession number.~~).

Determination of the full viral sequences from all three cases

Eventually, the full sequence of the present EEHV1A virus was determined from ~~material obtained from Umesh.~~tissue samples after necropsy by a next-generation Illumina paired-end procedure Sequences covering the complete viral genome were obtained from Umesh's samples, while the sequencing data obtained from samples from Omysha and Ruwani were less complete. Upon comparing the contigs obtained with the Umesh genome against the contigs from Omysha and Ruwani, 27,488 and 4,679 nucleotides, respectively, were unreadable in the two incomplete EEHV genomes. Excluding non-sequenced fragments, there were also 56 ambiguous positions in the Ruwani data, but otherwise the three genomes were virtually identical within the sequenced blocks. All raw sequencing data generated were uploaded to the Sequence Read Archive (SRA) as BioProject ID: PRJNA1052582. ~~The sequencing data obtained from samples from Omysha and Ruwani was less complete but by comparing the contigs obtained with the Umesh genome, 27'488 and 4679 NNNs were assembled in the two incomplete EEHV genomes, respectively. Excluding non-sequenced fragments, the genomes had a high similarity, e.g., only 56 nt mismatch between~~ The~~Umesh and Ruwani. Therefore, the~~ main focus of our further analysis was on Umesh's complete genome.

Overall ~~size and~~ genome structure and gene content of EEHV1A (Umesh, EP55)

The size of the assembled single complete intact genomic scaffold of Umesh (EP55) ~~obtained by a virion enriched next-generation Illumina paired-end procedure~~ was 179,847-bp (annotated as GenBank accession number OR543011). This included two near identical copies of the 2.9-kb direct terminal redundancy (TR), one located at each end. ~~Most other EEHVs that we have been involved with lacked a second copy of the TR most likely because they were carried out without the virion enrichment step.~~ Probably the most distinctive features of EEHV1 genomes are the two large-scale (20-kb and 40-kb) inverted core gene segments compared to cytomegaloviruses, the putative inverted repeat stem-loop origin of lytic DNA replication (ori-Lyt, 1190-bp), the two most likely novel nuclear immediate-early transcriptional control proteins E40 (ORF-K, 743-aa) and E44 (ORF-L, 1209-aa) and the largest ~~non-core~~ likely tegument protein E34 (ORF-C, 1886-aa), which are all present and intact with close to the same ~~inverted repeat stem~~ structures and ~~amino acid~~aa sequences as those found in all previously studied EEHV1 genomes. Other ~~unique~~unusual but consistent features of EEHV1~~,~~ strains include the likely captured cellular genes E4 (vGCNT1~~),~~) and E47 (vFUT9), ~~U73 (OBP), three~~plus four versions of vOX2-like proteins E23 (vOX2-4), E24 (vOX2-3), E25 (vOX2-2~~)~~), and E54 (vOX2-1), as well as the UL9-like U73 (OBP) and both U27.5 (RRB) and U48.5 (TK) ~~plus a total of 10x~~(also all missing in cytomegaloviruses) and eight conserved *Proboscivirus*-specific vGPCR-like proteins including E3 (vGPCR6), E5 (vGPCR5), E15 (vGPCR4), E20 (vGPCR4A), E21 (vGPCR4B), E26 (vGPCR3), U12 (vGPCR2~~),~~) and U51 (vGPCR1~~), E50.5 (vGPCR9) and E59 (vGPCR10) and~~) within a large family of ~~18 RAIP-~~26 retinoic acid inducible protein 3 (RAIP3)-like 7xTM domain receptors. These are ~~also~~ all present~~. With~~ within the Umesh genome and with just a small number of exceptions, the same applies to the majority of the rest of the expected total of ~~about 120 genes of the virus. Previous assessments of predicted ORFs and of in-frame splicing plus exon donor and acceptor motifs and intron sizes and boundary patterns for the rest of the~~

Formatted: Font color: Auto

~~protein coding genes were originally based largely on other known mammalian herpesviruses and especially on consistency across multiple independent EEHV1 strains~~close to 120 genes of the virus.

Fig 6. Similarity heatmap. BLAST-N alignment of the complete Umesh (EP55) genome against closely related EEHV genomes in the GenBank database using the VIRIDIC tool. The heatmap shows the similarity values for each genome pair, the fraction of genomes aligned and the genome length.

~~Almost 40 of the genes encoded by EEHV1 that are also found (albeit as highly diverged versions) within the majority of other mammalian herpesvirus sub-families are referred to as "core" genes with an applied U number nomenclature system derived from that used originally for human HHV6 genome analysis. In comparison, all of the remaining 78 protein encoding genes that are novel and not found in any other herpesviruses are given a sequential unique E number nomenclature system based on that originally applied to the prototype EEHV1 (Kimba) genome (11). For individual protein names, where feasible we have tried to apply the most universally agreed upon three-letter abbreviation for those proteins when some previously available functional or homology data is available from other well-studied herpesvirus species, and have otherwise used a combination of either an extended ORF-A to ORF-S system as originally suggested by Ehlers (17) or just left them with the same E number as for the designated gene name where little else is known for novel *Proboscivirus*-specific proteins.~~

Comparison with other EEHV1A and EEHV1B genomes

Previous experience in EEHV1 whole genome analysis has revealed that all individual independent strains display ~~unexpectedly~~ high levels of overall genetic divergence associated with a large subset of hypervariable genes. Across the entire population of EEHV1 ~~genomes~~strains examined the most striking feature is that many of these ~~numerous significantly diverged~~ variable genes ~~nearly always~~themselves cluster into multiple highly diverged cladal subsets that ~~can be~~have been designated as specific subtypes. ~~It is also evident~~

~~that~~Furthermore, most (but not all) EEHV1 variable genes often show these large and complex differences at both the protein (amino acid) level as well as at the DNA (nucleotide) level. ~~Fig~~Figure 6 shows the results of a search for the overall level of intergenomic ~~similarities~~DNA sequence divergence, which revealed that the closest ~~matched of~~match among the other nine complete EEHV1A genomes contained ~~in~~within the GenBank database (May 2024 version) is the Indian strain EEHV1A (IP43) [MN366291]. The latter differs from Umesh (EP55) by 2.7% at the nucleotide level (4,856-bp mismatch) as determined by direct pairwise comparisons using the VIRIDIC tool (~~34~~38). It also differs from our prototype EEHV1A (Kimba) genome [KC618527] by 4.6% (9,712-bp mismatch) and is least closely related to the EEHV1A (~~IP143~~IP165) genome [~~MN366294~~MN366292] with 7.5% divergence (13,488-bp mismatch). All of the other intact EEHV1A ~~intact~~ genomes fall within the range between IP43 and ~~IP143~~IP165. For the ~~single~~ prototype chimeric ~~sub-species~~subtype EEHV1B (Emelia) genome [KC462165] from ~~*Elephas maximus*~~Asian elephants the divergence is 8.2% (14,747-bp mismatch) and for the ~~single~~ orthologous prototype EEHV6 (NAP35) genome [MZ822422] from ~~*Loxodonta africana*~~African elephants the divergence is 26.2% (47,120-bp mismatch).

~~After detailed BLASTX~~Analysis of open reading frames and ~~BLASTP comparisons of all 122 genes individually at both~~likely splicing motifs within the EEHV1A (Umesh, EP55) complete genome revealed that it encodes a total of 121 predicted viral proteins that are named and listed under GenBank accession numbers WNZ34470.1 to WNZ34590.1 including 17 that involve in-frame splicing. After detailed BLAST-X and BLAST-P analysis plus clustal-W and visual six-frame inspection of all potential individual ORFs at the DNA and protein level relative to all other ~~sufficiently~~ well-characterized EEHV1 strains, the Umesh genome ~~of Umesh~~ proved to display similar ~~overall~~ features and gene organization as well as the typical ~~sporadic non-uniform~~ patterns of gene content divergence~~. It is noticeable that Umesh~~, but it does not ~~actually contain~~have any extra or novel genes not seen before in at least a subset of

EEHV1 strains. ~~But despite that statement, this does not imply that it contains exactly the same set of genes or gene subtypes as any or all other EEHV1 strains. In fact, there are indeed a small number of overall differences in both gene content and genome organization and no other previously evaluated strains have the exact same subtype patterns for the many designated hypervariable genes. Just like all the other strains evaluated~~Overall, Umesh consists of a complex mosaic pattern of mostly ~~well~~highly-conserved ~~genes~~proteins intermingled together with a distinctive and unique pattern of subtypes for the ~~moderate and highly~~30 or so variable ~~ones.~~

~~In general, although our earlier analysis at several short PCR loci predicted that Umesh would most likely prove to be an example of an EEHV1A not EEHV1B subspecies, to be certain that this was valid~~proteins typically found across ~~the whole genome, we needed to evaluate all 12 individual gene loci that almost universally and consistently define the relatively rare chimeric EEHV1B sub-species as distinct from the more commonly encountered non-chimeric~~ EEHV1A ~~sub-species. These all map within three major non-adjacent multi-gene chimeric domains in the central regions of the genome referred to as CD-I (gB/POL), CD-II (ORF-J/gN/gO/gH and TK) and CD-III (UDG, gL/ORF-O/ORF-P/ORF-Q), which together encompass nearly 13-kb and with one exception (POL) differ by at least 10% at the protein level between the prototypes of the EEHV1A (Kimba) or EEHV1A (Raman) sub-species and the EEHV1B (Emelia) sub-species~~ genomes (~~5, 10~~1, 28)~~.~~

, but has none of the characteristic features typical of EEHV1B diaspora strains (4). Although EEHV1B (Emelia) is the only completely sequenced member of the 1B ~~sub-species,~~subtype there are ~~eight~~seven other partially characterized EEHV1B ~~genomes, namely NAP14 (Kiba), NAP33 = NAP34, NAP38, NAP45, NAP47A, NAP49 and IP93 that almost universally also~~ strains that have ~~B~~nearly identical EEHV1B-specific characteristics at ~~all~~each of these same ~~loci. Furthermore, there are also three other frequently analyzed gene loci that display universal B sub-species features, but only differ by less than 10% from the 1A prototype e.g.~~

~~U51 (vGPCR1), U71 (MyrTeg) or U73 (OBP) that have all proven to have universally linked B-subtype versions also, plus four others that are only partially linked to the rest of the 1B diaspora e.g.E24/E25 and E30/E31. For example, only NAP14 and NAP49, but not Emelia, NAP33 or NAP45 have the highly diverged subtype-B pattern for the E24/E25 locus and only Emelia, NAP14, and NAP49, but not NAP33 or NAP45 have the B-subtype pattern for the E30/E31 locus. Finally, there are also three variable genes among~~ three CD loci. On the other hand, Umesh does contain the novel ~~7 x TM domain proteins that each split cleanly into three distinct subtype clusters, but that have no linkage or obvious connection whatever to the classic 1A versus 1B chimeric patterns nor to each other (E3/vGPCR6), E5/vGPCR5 and E26(vGPCR3).~~E36A (ORF-N) vCXCL chemokine-like protein. The latter is present in all EEHV1A strains as well as in EEHV2, EEHV5 and EEHV6, but is absent from all EEHV1B strains as well as from EEHV3 and EEHV4.

Comparison with main segment EEHV1 variable gene subtype assignments

~~Full evaluation~~

A list of ~~Umesh (EP55) shows that it does not contain even a single gene locus that exhibits a 1B-like pattern and it does also contain~~ the ~~single small E36A (ORF-N) vCXCL chemokine-like protein that is unique to~~ EEHV1A ~~strains and absent from all EEHV1B strains. In contrast, the Umesh~~(Umesh) assigned subtypes at each of the ~~19~~20 most variable ~~gene~~protein loci ~~included above are as follows: E3 (vGPCR3 = A1; E5 (vGPCR5) = C; E24 (vOX2-3 = A, E25 (vOX2-2) = A, E26 (vGPCR3) = B; E30 = C1, E31 = C, E39 (gB)= C1, E38 (POL) = A, E35 (ORF-J) = A, U46 (gN) = C1, U47 (gO) = A1, U48 (gH) = D, U48.5 (TK) = A, U51 (vGPCR1) = E, U71 (MyrTeg) = A, U73 (OBP) = A. U81 (UDG) = A, U82 (gL) = A1, E37 (ORF-O) = C, E38 (ORF-P) = E, and E39 (ORF-Q) = C. A detailed comparison~~from the main segment of ~~this information for Umesh with~~ the ~~equivalent subtype cluster assignments for the same set of 22 variable genes from nine other selected~~ genome is shown ~~EEHV1 strains is presented~~ in the upper ~~section~~Section I of supplementary Table S1. The particular virus

Formatted: Font: Not Bold

genomes chosen as illustrative examples ~~chosen to be shown~~ here include the original three complete genome ~~protoypes~~prototypes from GenBank of EEHV1A (Kimba), EEHV1A (Raman) and EEHV1B (Emelia), plus accumulated ~~gene~~sub-genomic locus data from six of the best characterized but incomplete EEHV1A genomes determined previously by conventional PCR amplification-based DNA sequencing of older lethal EHD cases as carried out by ~~our~~the Johns Hopkins University ~~Laboratory~~Viral Oncology group. Specifically, this data was derived from one sample from India (IP11), one from Europe (EP07, Xian) and four from the United States (NAP18, NAP26, NAP17 and NAP21).

Other key details about these ten EEHV1 strains are listed in Table S2**,** including where appropriate GenBank accession numbers for complete genomes as well as for six selected representative sub-genomic loci. With the exception of the prototype EEHV1A (Kimba) strain, which (although it does have three Cs within CD-II) was otherwise designated to have just A or A1-subtype variable genes across the entire genome, all other non-1B diaspora strains fall into complex mosaic patterns like this with largely unlinked variable gene subtypes.

The most useful region to evaluate here has proven to be the combined ORF-O/ORF-P/ORF-Q complex (part of CD-III) for which we recognize ~~13~~a total of thirteen different ~~triplex~~inserted alternative three-gene patterns, with the same C-E-C subtypes as in Umesh ~~(EP55)~~ being found also in ~~11~~eleven out of a total of 52 other EEHV1A or EEHV1B ~~cases~~strains that have been fully evaluated previously at this locus~~, namely~~. Those include the viruses from European cases Raman~~, EP14,~~ and Plai Kiri as well as from IP43, NAP16, NAP24, NAP43, NAP73, NAP75, NAP88, NAP91 and NAP93. Although only two of those examples are available as complete genomes (Raman and IP43), none of them occur within known EEHV1B strains. In addition, only one of the seven India cases evaluated here (IP43) has that same C-E-C group pattern~~ and only two others have been identified among the relatively few European EEHV1A cases examined in detail so far (i.e. Raman and EP14 but~~

~~not EP07 or Umesh). Beyond those specific loci discussed above that are either fully or partially related to the 1A/1B sub-species chimeric domain patterns there are also another three variable genes among the novel 7 x TM domain proteins that each split cleanly into three distinct subtype clusters, but that have no linkage or obvious connection whatever to the classic 1A versus 1B chimeric patterns nor to each other i.e. E3 (vGPCR6), E5 (vGPCR5 and E26 (vGPCR3).~~.

Formatted: Line spacing: Double

Comparison with other EEHV1 R2-segment gene arrangements

Similarly, many of the ~~11~~eleven potential ~~gene~~protein coding positions located within the short 10.5-kb R2-segment of all EEHV1 genomes display high levels of variability and subtyping that have proven to be ~~either~~ completely unrelated to the linked 1A/1B ~~chimerism diaspora~~chimeric domain pattern explained above ~~(or else are very highly scrambled).~~. These latter non-1A/1B diaspora related hypervariable gene loci all map within the novel *Proboscivirus*-specific segments located near both ends of the linear EEHV1 genomes. ~~Amazingly, although~~Although the R2-segment mapping between E47 (vFUT9) to E55 (vIgFam3) encompasses just 5% of the length of the EEHV1 genome, it has multiple levels of additional complexity ~~that together provide a large fraction of the overall EEHV1 genomic variation. Furthermore, we have interpreted that this part of the complexity within the R2-segment involves multiple alternative two or~~including involving alternative three-gene "cassettes" that define eight distinct clustered R2 sub-groups designated A, B, C, D, E, F, G and H. ~~These latter cluster groups~~Each strain carries just one of these triplex cassettes and there are three different potential locations where they can be inserted. Therefore, these clustered groupings represent ~~a~~ major ~~determinant~~determinants of EEHV1 genomic organization ~~within non-1B strains~~.

~~The presence or absence of these eight types of inserted multigene "cassettes" as well as which of three different potential genomic locations where they are inserted at largely defines~~

~~each R2 group. However, unlike~~Unlike the 20 known available “optional” R2~~-~~-family genes present ~~in~~within the ~~“~~cassettes~~”~~,, which are each found in just a small subset of the overall EEHV1A strain population, there are also eight remaining “constant” R2-segment genes~~,~~ (see Table S1) that are almost always all present~~,~~ but are also mostly highly diverged and form many additional subtype clusters of their own. The latter include two interesting ~~single~~captured host genes E47 (vFUT9) and E54 (vOX2-1) ~~and~~plus six ~~other~~ members of ~~R2-multigene~~ multigene paralogous R2-segment gene families ~~that are largely common to all strains.~~ ~~Therefore, although~~.

Although each individual EEHV1 genome usually contains a total of just ~~11~~eleven genes (often including an average of two or three fragmented or inactivated ORFs) within the entire R2-segment, the overall ~~known~~ population of ~~37~~38 distinct independent EEHV1A plus EEHV1B virus strains that have been evaluated fully across the R2-segment encodes ~~as many as 28 different total alternative available genes.~~a total of 28 different available alternative genes. The presence (+) or absence (-) as well as the relative location and subtype designation of 20 of these alternate genes found within the R2-segments of either Umesh itself or one or more of the set of nine reference EEHV1 genomes are summarized in Section II of the supplementary Table S1**.** Note that only four of the total of eight known organizational patterns of alternative R2-segment insertion cassettes are represented here (i.e. A, B, D and F groups, but not any examples of the C, E, G or H groups).

These other ~~“potential”~~ R2-segment genes include seven ~~paralogous~~ more alternative versions of ~~RAIP-related~~ vGPCR-receptors, namely E48 (vGPCR7), ~~E56 (vGPCR8),~~ E50.5 (vGPCR8/vGPCR9/vGPCR13), E56 (vGPCR12), E59 (vGPCR10~~)~~, and E62 (vGPCR11~~),~~ ~~E56 (vGPCR12) plus~~) from within the large 7 x TM RAIP3-like family that each occurs in just a subset of strains. Every EEHV1 R2-segment includes either just one or two of the latter with Umesh itself having versions of both E50.5 (vGPCR9) and E59 (vGPCR10) but not any of the others. There are also a total of 18 paralogous diverged alternative versions of ~~(~~mostly~~)~~

membrane-bound immunoglobulin-domain (Ig) family ~~(~~proteins (designated vIgFam1 to vIgFam15) ~~proteins, of which some~~distributed amongst known EEHV1 strains. Umesh encodes its own versions of five of these, including three that are constant e.g. E50 (vIgFam1), E53(vIgFam2.5) and E55 (vIgFam3) plus two of the alternates E60 (vIgFam6) and E61 (vIgFam7). Some of these are detectably related to host cell protein CD48~~, and a~~. Finally, there is also a small third family of four other rather non-descript alternative R2-segment membrane proteins (E49, E51, E64 and E68). ~~The presence (+) or absence (-) plus the relative location and subtype designation of twenty representative genes found within the R2-segments of both Umesh and the same set of nine other selected comparative examples of EEHV1 genomes are summarized in the lower section of supplementary **Table S1.** Note that only four of the eight known organization patterns of alternative R2-segment insertion cassettes are listed here (i.e. A, B, D and F groups, but not C, E, or G).~~ From ~~amongst~~among the ~~known~~ total of 28 ~~possible~~known alternative R2-genes, the Umesh ~~(EP55)~~pattern most closely matches that of the Ganesh (NAP26) and IP43 ~~patterns~~ by having a typical group-D E59/60/61 three-gene triplex cassette ~~and encodes~~among the ~~following 11~~total of eleven R2-segment encoded proteins overall ~~with their assigned subtypes as shown i.e. E47 (vFUT9) = B, E50.5 (vGPCR9) = C, E51(membrane) = D1 (frame-shifted), E52 (vIgFam2) = deleted, E59 (vGPCR10) = D, E60 (vIgFam6) = +, E61 (vIgFam7) = + (frame-shifted) , E52.5 (vIgFam2.3) = deleted, E53 (vIgFam2.5) = frame-shifted, E54(vOX2-1) = o and E55 (vIgFam3) = A2.~~(see Table S1). Unlike the other R2 genes described here ~~neither~~three of the vIgFam proteins (E60, E61 ~~or~~and E53 ~~proteins~~) do not show any significant subtype variability, whereas in ~~dramatic~~ contrast the 43 examples of E54 (vOX2-1) proteins evaluated despite collectively exhibiting up to 15% overall variability at the aa level ~~they~~ do not cluster into any readily definable subtype groupings. ~~Furthermore, although~~In contrast there are just two tight major subtype clusters of E47 (vFUT9) in which the prototype EEHV1A (Kimba) version (subtype-A) and the prototype EEHV1B (Emelia)

version (subtype-B) differ by as much as 32% at the protein level. Nevertheless, this particular additional example of subtype clustering is apparently not related in any way (or at least not linkedlocus shows no obvious linkage to) the classic 1A/1B subspecies subtype diaspora. In fact, from among the 35 total E47 (vFUT9) enzyme-encoding genesversions evaluated here there are more24 examples of the B-subtype (24x) thanincluding Umesh) and only eleven of the A-subtype (11x), with both seeminglybeing randomly assorted among the otherwise nominally A sub-species versus B sub-speciesEEHV1A and EEHV1B diaspora genomes.

Overall, we consider the eight different two oralternative three-gene inserted "cassette" types to be groups provide the most definitive way to classify EEHV1 genome patterns within the R2-segment, and note that once again. Here the Umesh strain clusters within the largest set (= group-D) here along with ten other group-D casesexamples (namely Daisy, IP43, EP07Xian, EP20 = EP21, NAP30, NAP31 and NAP41 = NAP47), including three from Europe (EP07Xian, EP20 = EP21), but just one from India (IP43). Moreover, there are no known group-D inserted cassette patterns (i.e. E59/E60/E61) that fall amongst the six known EEHV1B genomes that have been fully evaluated across thefor which R2-segment. However data is available. The next two most abundant cassette patterns are seven group-As and six group-Bs, with the remaining 15 examples being three -Cs, two -Es, four -Fs, two -Gs and four -Hs. Among the latter, there are several moreother anomalous situations where a total of 10ten virus strains havedisplay either just a two-gene cassette (groups-C, -E and -G) andor four others have both a group -B-group like three-gene cassette plus a two-gene group -H-group cassette. The next two most abundant " cassette" patterns beyond the D-group are 7 x As and 6 x Cs, with the remaining 15 examples being 4 x F, 2 x E, 3 x C, 2 x G and 4 x H. (not shown).

Finally, in terms of detailed gene-by-gene analyses, there are two alternative published physical genetic maps of prototype EEHV1A genomes that Umesh (EP55) can be compared

~~to i.e. those of EEHV1A (Raman) from Wilkie et al~~ Finally, for completeness note that Section III of Table S1 includes examples of subtype designations applied previously to five core conserved genes from these and many other EEHV1 genomes based on selected small PCR-sequenced loci including E38 (POL) that just display low nucleotide level variability without significant associated protein level variation (~~10~~1, 3, 4, 35)~~ and EEHV1A (Kimba) from Ling et al (11), but note that they are drawn in opposite orientations and use different gene nomenclature systems. Furthermore, the Umesh genome described here and all other intact annotated EEHV1, EEHV2A, EEHV5B and EEHV6 genomes that we have submitted to GenBank since the original EEHV1A prototype have the same orientation and nomenclature as used for Kimba (11), except that just the R2-segment itself had to be switched to the extreme left-hand-side once the actual linearizing packaging cleavage site was determined (10). Curiously, none of the GC-rich branch *Proboscivirus*es EEHV3A, EEHV3B nor EEHV4A or EEHV4B complete genomes even have the R2-segment (18).~~

.

Overall, the sequencing results confirmed that all three ~~elephants~~ EHD cases here had been ~~hit~~infected by the same novel strain of EEHV subtype 1A. More specific Umesh ~~(EP55)~~ data relating to both E39 (ORF-Q) serology and three viral-encoded enzymes of interest in the context of epidemiology and antiviral treatment are presented below.

~~**Fig 6. Similarity heatmap**. BLASTN alignment of the Umesh (EP55) genome against closely related EEHV genomes in the GenBank database using the VIRIDIC tool. The heatmap shows the similarity values for each genome pair, the fraction of genomes aligned and the genome length.~~

~~Analysis of the E39/EE3 ORF-Q locus~~

~~Antibodies against a particular ORF-Q clade have previously been associated with a mild course of EEHV1 infection, whereas their absence have been linked to severe EHD (7). Having established that the present virus was different from the two previously observed ones, it was of interest to determine its ORF-Q (E39/EE3) sequence. Therefore, the E39 locus~~

of the virus in Umesh's samples was sequenced and the translated amino acid sequences were aligned to generate a phylogenetic tree (Fig 7). According to this analysis, the Umesh (2022) isolate was nearly identical with other ORF-Q Clade C isolates (EP14 and 24; NAP43, 73, 75; IP43) but clearly different from Clades A (NAP11, 16,18, 23, 26, 32, 39, 41, 47, 80; IP11, 164), D (EP07 (Xian), EP21; NAP20,29,30,31,72, IP07), B (NAP45, 49), E (NAP21) and F (NAP17) and also from EEHV6 (NAP35), whose ORF-Q sequences were used for routing of the tree. Unfortunately, these results came too late to consider a serum transfer from one of the Clade C-positive animals. Together, these epidemiologically relevant data stated that a previously unrecognized, newly detected strain of EEHV1A had been the cause of the present EHD outbreak.

Fig 7. ORF-Q phylogenetic tree. A maximum likelihood phylogenetic tree was obtained from the alignment of ORF-Q amino acid sequences using the Neighbor Joining method and 100 replicates to perform bootstrap analysis. The branch length is indicated for major branches, dividing the tree into the known clades A through F. Each strain is identified by its case number followed by its GenBank accession number.

Further analysis of the Umesh E39/EE3 locus and comparison to other ORF-Q subtypes

Antibodies against a particular ORF-Q clade have previously been associated with a mild course of EEHV1 infection, whereas their absence has been linked to severe EHD (7). Having established that the present virus is different from the two previously observed ones, it was of interest to determine Umesh's complete ORF-Q protein sequence. Furthermore, because ORF-Q proteins clearly have significant and relevant antigenic properties as well as providing one of the most extreme and important examples of EEHV1 divergence and subtyping, an updated ORF-Q phylogenetic tree was also generated showing the alignment results for the intact translated protein sequences from 33 independent strains as presented in Figure 7. The year 2022 Umesh isolate proved to be nearly identical to other ORF-Q clade C isolates such as Plai Kiri and Raman plus NAP43, 73, 75 and IP43), but highly diverged from clade A (NAP11, 16, 18, 23, 26, 32, 39, 41, 47, 80; IP11, 164), clade D (Xian, EP21; NAP20, 29, 30, 31, 72; IP07) and clade B (NAP45, 49) strains as well as from clade E1 (NAP21), E2 (IP91,

165) and F (NAP17), and further still from EEHV6 (NAP35). Quantitatively, the average percentage protein identity values when compared against the 364 aa version encoded by Umesh as measured by the NCBI BLAST-P program proved to be 98-99% for the other members of clade C, 77-82% for clade A, 45-47% for clade D, 30% for clade F, 28% for clade E2, 26% for clade E1, 26% for clade B and 33% for EEHV6 (NAP35), whose sequence was used as outgroup for rooting the tree. Unfortunately, these results came too late to consider a serum transfer from one of the clade C-positive animals. Together, these epidemiologically relevant data indicate that a previously unrecognized, newly detected strain of EEHV1A had been the cause of the present EHD outbreak.

Analysis of the U48.5/EE7 ETk locus

As the ETk may contribute to the success of antiviral treatment with nucleoside analogues, it was also of interest to compare the predicted ~~amino acid sequence~~protein sequences of ~~the present EEHV1A isolate~~Umesh Etk against previously known EEHV1 ETks. Indeed, the aa sequence was highly conserved among reference EEHV1A isolates, ~~displaying just~~with only one aa difference (H278N) against the sequence from the European EEHV1A prototype (Raman) and two aa (V152I and H278N) against the American prototype (Kimba). This strong aa conservation of the predicted enzyme among ~~only remotely related~~a wide variety of EEHV1A strains strongly speaks for a biological role of the Etk, although its *in vitro* and *in vivo* activity against the presently used antiviral compounds remains to be determined.

Analysis of the U69 ECPK locus

~~Similarly, the present ECPK~~ The aa sequences ~~were compared against those~~ of ~~other known EEHV isolates. Not unexpectedly, those aa sequences were~~Umesh ECPK are highly conserved ~~among~~ when compared to other EEHV1A strains. Not a single aa difference from the present sequence from Umesh was found in the sequences of the European EEHV1A isolates Raman or Plai Kiri. One single aa exchange (T409A) was detected upon comparison

against the EEHV1B isolate Emelia. The ECPK sequence of Kimba ~~comprised~~contained two aa differences, namely T408P and T409P. Since all these differences map outside of the predicted catalytic domain of the enzyme (aa 166 to 306), it will be of interest to address ~~the~~their residual biological functionality~~ of it~~ in the future.

Analysis of the U38 DNA polymerase locus

The viral DNA polymerase enzyme~~,~~ (POL), encoded by U38~~,~~ is~~ considered~~ essential for viral DNA replication. Phosphorylated nucleoside analogues may either block the enzyme ~~to~~thereby terminating its activity or integrate chain-terminating molecules into the growing DNA, which blocks further elongation. ~~It~~Therefore, it was~~, therefore,~~ of interest to compare this locus ~~in between~~from our three isolates ~~as well as ~~against other published ~~amino acid~~protein sequences in the databases. ~~Notably, the~~The sequence conservation among our three isolates at this locus was 100%, whereas two to eight ~~amino acid~~aa changes were observed upon comparison of ~~our translated sequences~~the Umesh sequence against ~~the amino acid sequences~~those from ~~10~~ten previously published cases. Interestingly, ~~a single one of these differences in our isolates opposed to high conservation among all others, i.e., ~~Leu 792 occurred in our sequence compared to Isoleucine at this position in all ten previously published isolates (L792I). ~~Moreover, this~~This seeming mutation mapped to the center of a conserved domain, which had been associated with predicted enzymatic activities, including elongation, DNA-binding and dNTP binding. All other differences had been previously described for other ~~U38~~Etk proteins.

Discussion

Formatted: Line spacing: Double

Detailed knowledge of circulating viruses may influence treatment options.

With a history of two prior cases of EHD ~~in~~at the zoo and evidence of recurrent episodes of EEHV shedding and viremia between 2014 and June 2022, the risk for another outbreak of

EHD was considered high. In particular, the prevalence of EEHV1A, EEHV1B, and EEHV4 among the adult animals, and the presence of two seronegative animals in the susceptible age~~,~~ ~~the risk for another outbreak of EHD was considered high.~~ were documented (25). During four years of weekly screening, seven instances of low grade, transient viremia were detected, each ~~single one~~ caused by EEHV1, while viremia due to EEHV4 was never detected~~. These findings confirmed earlier observations of the presence and active circulation of EEHV1 among these animals.~~ (Table 3).

EEHV serology has only recently established its proper role in addressing EEHV prevalence among elephants (2, 7, ~~14~~13). Particularly LIPS assays have ~~allowed~~proved to allow us to discriminate between antibodies that cross-react among various EEHV types or strains and other antibodies that represent a specific immune response against a particular virus strain (7). We selected LIPS assays using the conserved, cross-reactive glycoprotein B- (gB) and the ~~strain~~subtype-specific ORF-Q-antigens, respectively, to assess the serological status of the animals against EEHV. The use of ProteinA/ProteinG-beads in the LIPS assays brings a great advantage for the veterinary medical purpose with it because different animal species can be addressed using the same assay. We took this advantage in our case by using horse sera to determine the negative cut-off values for all of our LIPS assays.

~~The sera of all~~All adult elephants had detectable levels of antibodies against ~~glycoprotein B~~gB of EEHV1A (gB1A~~). Moreover, four~~), which indirectly confirms active circulation of EEHV1 among the elephants. Notably, sera from four African elephants, ~~kindly~~which were provided for control purposes by the Basel zoo ~~in Switzerland,~~ , also yielded ~~also~~ varying degrees of antibodies against gB1A. The published ~~amino acid~~protein sequence of the EEHV6 ~~glycoprotein B~~gB (gB6) shares 88 to 89% ~~amino acid~~aa identity with gB1 of the subtype 1A strains from Kimba and Raman, respectively. ~~Interestingly the amino acid~~The protein sequence of gB subtype 1B (Emelia) shares only about 85% aa identity with either EEHV6 (NAP35) or EEHV1A (Kimba, Raman). Consequently, we assume that the gB-

antibodies detected in the ~~sera from the~~ African elephants were possibly a result of cross-reacting ~~and due to~~ subclinical infection with EEHV6.

The following observations were relevant for the three young animals at risk of EHD: At eight years of age and with a history of shedding EEHV1B ~~as well as several occasions of transient viremia, the seropositive status of Omysha could be attributed to having mounted its~~(25) as well as several occasions of transient viremia (Tab. 3), the seropositive status of Omysha could be attributed to having mounted her own active immune response against EEHV1. At two years of age and without previously detected episodes of viremia, the seropositive status of Umesh ~~was somewhat unexpected. Maternal antibodies were expected to be quite low at this age~~may be explained by low maternal antibodies, but as half-brother and playing companion to Omysha, it is possible that this individual had already mounted its own immune response against EEHV1 under the previous cover of maternal antibodies. The seronegative status of Ruwani at the age of five years was a matter of concern. ~~Neither~~No traces of maternal antibodies could be detected~~ any more nor~~, and an active immunity seemed not to have developed, although three episodes of viremia had previously been recorded~~.~~ (Table 3).

LIPS assays using ORF-Q antigens ~~revealed much~~detected fewer seropositive animals than gB-serology. As the subtype EEHV1B had been observed to be shed from the trunk of Omysha ~~(24)~~(25), it was not surprising that this animal showed the highest reaction among the members of the herd against the ORF-Q-B antigen. In contrast, the other young animals at risk did not have such antibodies nor were they seropositive against ORF-Q antigens A, C, or D. Of the older members of the herd, at least one female elephant ~~cow~~ (Ceyla) had significant antibody levels against ORF-Q-A, whereas two ~~elephant cows~~female elephants (Indi; Chandra) had preexisting antibodies against ORF-Q-C. Interestingly, none of the animals seemed to have significant levels of antibodies against ORF-Q-D antigen, although the isolate from a previous case of EHD in the zoo (~~EP07~~Xian, 1999) had been classified to belong to

ORF-Q ~~Clade~~clade D. These results suggested that antibodies against ORF-Q antigens may be short-lived and therefore that ~~the~~ young ~~animals were indeed~~elephants are at risk ~~upon~~during an EEHV outbreak.

Thanks to the preventive viremia-screening program, the 2022 outbreak was detected early. ~~Despite of various~~However, due to the “protected contact” principles, any treatment ~~attempts~~ ~~(~~option depended on the individual elephant’s decision to interact (30). Although Umesh agreed to intravenous GCV treatment (details to be ~~reported~~published elsewhere), the virus burden in the bloodstream increased dramatically (Fig. 3) and ~~6~~six days after viremia was first detected, Umesh ~~succumbed to~~died of EHD. The second animal (Omysha) rebounded twice from viremic to aviremic and back but then still succumbed to EHD. Notably, Omysha did not agree to GCV treatment but consented occasionally to take up some oral dose of FCV. Neither GCV nor FCV had a noticeable effect on the ongoing viremia (Fig. 3). The third animal (Ruwani) had its first ~~episode~~positive reading of viremia at the same day as the second animal (Omysha) but had a survival time of three weeks after ~~the first episode of viremia.~~that, even without consenting to any treatment option. Only ~~upon~~after its fifth ~~episode~~detection of viremia, the virus burden in the blood stream increased with almost ~~each~~every consecutive sample and the animal eventually succumbed to the disease.

Once the outbreak was ongoing, it was of immediate interest to subtype the causative virus more closely. Indeed, initial PCR-sequencing of the E36~~/U79~~ gene locus ~~ruled out any~~ suggested the involvement of a subtype ~~1B~~1A strain ~~and identified, instead,~~rather than a subtype ~~1A~~1B virus as the ~~present~~current disease-causing agent. This conclusion raised new concern about the eight-year-old Omysha, which had been found seropositive for antibodies against the ORF-Q-B antigen but not against other ORF-Q antigens. Moreover, the possibility to use her antibody-rich serum for a potential treatment of the other animals at risk was now ruled out. As maternal antibodies seem to be able to confer protection, a rapid analysis of the prevailing virus subtype may help to identify quickly a suitable herd mate as serum donor (7).

Subsequently, it was inviting to speculate that one of the previously detected 1A ~~strain~~strains, dating back to 1999 and 2003 had re-emerged after long years of latency, which had been the case in Berlin, with Shaina Pali and Ko-Raya (EP23 and EP25) being identical to Plai Kiri ~~(EP14)~~ from 11 years earlier (1). Yet, a comparison of E54~~/EE51~~ sequences, including the nucleotide sequences of the Xian (1999 ~~(EP07~~) isolate and ~~the present virus~~Umesh revealed significant differences. Thus, the present EEHV1A virus was newly detected throughout this outbreak~~, raising~~ ruling out the ~~question about its source.~~re-emergence of a previously identified latent strain. These analyses during the ongoing outbreak had ~~apparently~~ direct effects on our risk assessment ~~as well as~~and may have also expanded our treatment options. However, rapid identification of the ORF-Q subtype of the prevailing virus had not been established at the time of the outbreak and ORF-Q serology using subtype C and D antigens were only established in retrospect. Otherwise, Indi and Chandra, both seropositive for ORF-Q-C antibodies (Fig. 2), may have been used as serum donors for antibody transfer.

Evidence that the same new EEHV1A strain infected all three calves

Only once the outbreak of EHD was over, could the entire EEHV1 genomes from all three cases ~~could~~ be determined~~, upon which the previous~~ and compared with the result of the initial PCR ~~analyses were confirmed.~~analysis. Moreover, the sequences of other genomic loci of interest then became available for interpreting the course and origin of the disease~~, in particular the epidemiological chain, and~~ as well as the failure of treatment.

Concerning the epidemiological chain, it is important to ~~state~~re-emphasize that all three isolates had the exact same genomic sequence, belonging to the EEHV1A subtype ~~1A~~. The E54~~/EE51~~ vOX2-1 locus was 100% conserved among the three current isolates, which confirmed ~~the~~their common origin, but was significantly different from the Xian (1999 ~~EP07~~) isolate. ~~Analysis~~Furthermore, analysis of the E39~~/EE3~~ locus of Umesh ~~(EP55)~~ identified a virus belonging to the ORF-Q-C clade. This finding was very surprising, since no such virus

had previously been identified among these animals~~ The ~~. Instead, the previous lethal EEHV1A~~,~~ strain responsible for the death of ~~EP07~~Xian, belonged to the ORF-Q-D clade, while serological evidence suggests that the EEHV1B isolate detected in earlier trunk-wash samples from Omysha belonged to the ORF-Q-B clade.

The ORF-P and ORF-Q genes of EEHV1, which arose by duplication and have some residual ~~homology~~similarity, are replaced in EEHV3/4 by a single ~~similar~~novel gene at the same location and with the same two exons (E39A, ORF-R), but with minimal or no homology to ORF-P or to ORF-Q itself (5). Similarly, EEHV2 and EEHV5 also lack ORF-Q but do have ORF-P. Accordingly, our serology was unable to evaluate the serological status of the animals towards EEHV3/4, although EEHV4 had been detected in the trunk wash sample from Thai~~.~~ the breeding bull and LIPS assays for the specific detection of antibodies against EEHV2 and EEHV3/4 have recently been established by others on the basis of the E34 encoded protein (14). Unfortunately, the ORF-Q sequence from Aishu, who died from EHD in 2003, was not available. However, the few known parts from the Aishu~~-~~ virus genome, for example the U48.5~~/EE7~~ ETk locus, differ considerably from the present sequences. Overall, these sequences confirmed the presence of a new previously undescribed virus strain among these elephants.

Potential antiviral drug targets

Three different EEHV1 ~~proteins~~encoded enzymes may be involved in the susceptibility or resistance of nucleoside-based antiviral treatment against EEHV: the viral thymidine kinase (U48.5~~/EE7~~, ETk), the conserved protein kinase (U69, ECPK), and the catalytic unit of the viral DNA polymerase (U38, DNA pol~~).~~)(16, 40-42).

Identities and differences, respectively, in the coding sequences within these three genes of our three isolates might not only convey information about potential drug-resistance but also about the origin and sequence of transmission. ~~If~~However, since all sequences from our three

isolates were identical, the source of infection must be the same for all three animals or the virus must have been transmitted from one case to the other without genomic alteration. Therefore, it is safe to assume that all three animals were infected from the same original shedder.

As its name suggests, the ECPK protein was even more conserved than ETk. Moreover, this enzyme ~~showed~~did show some *in vitro* activity, converting GCV and PCV in a dose-dependent manner, thus, making it a prime candidate ~~for~~not only for rendering the virus sensitive to those antiviral drugs but also for a possible emergence of a drug-resistant mutant virus (1, 41, 42). Although a few variations against previous sequences were detected, all the differences had been identified before, and mapped outside of the predicted catalytic domain of the enzyme (aa 166 to 306). ~~Despite of the long survival time of the third case, none of the observed mutations could explain the development of a drug-resistant phenotype and ECPK. Although the details of the treatment attempts during the outbreak were not disclosed to the authors of this communication, it~~In the present case, three different aspects have to be considered, which may explain the obvious inability of the antiviral drugs to confer any measurable effect on viral burdens as well as on the clinical outcome of the disease: (1) ECPK and ETk are less active than HSV Tk in converting the prodrugs to their active form, which may negatively affect the desired drug activities. (2) It is possible that the U38 DNA polymerase may be naturally incompetent to incorporate certain phosphorylated nucleoside analogues into the growing viral DNA. ~~Due to~~Because of the lack of suitable cell cultures, ~~the role of the U38 protein in EEHV replication and its susceptibility or resistance against nucleoside analogue treatment~~this issue has not yet been addressed. Still, an interesting variation was observed within the U38 DNA ~~pol~~polymerase sequence, where a single mutation (L729I) in all our new isolates opposed to the high conservation among ten randomly selected previous isolates. This seeming mutation mapped to the center of a conserved domain, which had been associated with predicted enzymatic activities, including

Formatted: Font color: Red

elongation, DNA-binding, and dNTP binding. (3) Because the treatment efforts strictly depended on the affected animal’s consent, it is also possible that doses of antiviral drugs did not meet the needed concentrations in the bloodstream.

Presumed long-term quiescence in a herd-mate

Having established that all three animals had been infected most likely from the same source with the same but previously unrecognized virus, its most probable origin can be attributed to an elephant who was born outside of the Zurich zoo. Indeed, ORF-Q serology identified two individuals, Indi and her daughter Chandra, which had been ORF-Q-C-seropositive prior to the outbreak, while all other members of the herd, in particular the newest member Thai and the oldest external animal, Ceyla (born 1975 in Sri Lanka, transferred to the Zurich zoo in 1976), had been seronegative. As one of the ORF-Q-C-positive animals Chandra had been born and raised in the zoo, whereas her equally seropositive mother (Indi) had been born 1986 in Myanmar, was then imported to Switzerland in 1988, before being transferred to the Zurich zoo in 1999~~), it~~). It is tempting to speculate that Indi has acquired this “novel” virus in her youth but kept it latent or did not spread it until prior to the present outbreak.

Global protein subtype analysis of the intact genome of EEHV1A (Umesh, EP55)

The large DNA genomes of EEHV1 and the six other identified *Proboscivirus* species (EEHV2 to EEHV7) range between 168 and 206-kb in size encoding approximately 120 genes each and have amongst the most variable DNA genomes of all mammalian herpesviruses. This extreme hypervariability is largely confined to within between six and eight non-adjacent protein encoding loci that we refer to as chimeric domains (CD-I to CD-VIII) whose characteristic number, location and size differ somewhat across each species, as well as the gene content within the 4 to 10-kb R2-segment when present. In particular,

extensive genomic analyses and comparisons of the hypervariable domains of the most variable EEHV1 proteins show that they often cluster tightly into multiple (3 to 8) distinct cladal patterns that have been designated as subtypes A, B, C, D, E etc. In the special situation of the chimeric EEHV1B genomes the individual gene subtype patterns of the non-adjacent CD-1, CD-II and CD-III variable loci across large sections of the genome are linked, but there is very little linkage observed between the subtype patterns across different EEHV1A strains. Here in the case of the lethal EEHV1A (Umesh, EP55) strain that infected three young Asian elephant calf mates within just a few days of one another, we have determined the complete 175-kb genomic DNA sequence with full annotation of the gene coding content and compared it with nine other selected representative well-studied EEHV1 genomes in supplementary Tables S1 and S2). In particular, we have focused especially on the designated subtypes of all of the 33 most significant hypervariable viral proteins including those contributing to the uniquely R2-segment gene content. The results show that the combined individual protein subtypes obtained for EEHV1A (Umesh) conform to the general expectations of a novel overall mosaic pattern with considerable subtype level divergence across just a scattered subset of hypervariable proteins compared to all other evaluated strains. For Umesh this includes falling into the D-subgroup R2-segment cluster together with two other reference strains (Xian and Ganesh) as defined by the presence of the adjacent E59 (vGPCR10) plus E60 (vIgFam6) and E61 (vIgFam7) genes as their characteristic alternative inserted R2-triplex cassettes. Among the particular ten reference EEHV genomes evaluated in Table S1**,** Umesh is also the only one to encode an E-subtype U51 (vGPCR1) protein as well as the only one to encode a D-subtype U48 (gH) protein. It also shares the C-E-C subtype pattern for the ORF-O, ORF-P, ORF-Q main segment glycoprotein complex only with EEHV1A (Raman).

The fact that EHD evidently involves primary infection in inappropriately or insufficiently immune calves (7, 14, 15) appears fully consistent with the universal finding of the identical

species and strain of EEHV infecting two (or more) elephant calves that contracted DNA-positive confirmed EHD nearly simultaneously at the same housing facility. These occurrences have now been documented at least a dozen times previously, including in Germany and multiple times in the United Kingdom and USA plus three times also in Asian range countries India (3, 28), Indonesia (43) and Malaysia (44), although only in Switzerland and Malaysia have the episodes involved as many as three separate lethal EHD cases within young calves with the identical virus strain within a very short time period.

Conclusions

Formatted: Line spacing: Double

Despite considerable preventive efforts and early detection of EEHV1A viremia, the Zurich zoo lost three young elephants to EHD. The reasons for the failure of the preventive measures may be summarized in three points: (1) Presence of a previously undetected EEHV1A strain among the resident elephants. ~~Probably,~~Possibly, it could be deduced that the new virus re-emerged after almost 40 years of latency from one of the oldest elephants in the zoo. (2) Insufficient or absent strain-specific immunity in the affected animals. (3) Lack of efficient antiviral drugs and protective vaccines, due to the unavailability of appropriate cell cultures and animal models. Having determined the complete genomic DNA sequence of EEHV1A (Umesh, EP55) during the course of this outbreak, we also took the opportunity to make detailed comparisons of the overall gene content and hypervariable protein subtype clustering patterns with multiple other EEHV1 strains leading to a new level of appreciation of the often very high levels of polymorphic divergence within a subset of the proteins encoded among and across these genomes, especially within the R2-segment. The complexity of those differences with major and minor variations has to be accounted for in the planning of future strategies for prevention and treatment of EHD. Based on the obvious conservation of the three enzymes responsible for sensitivity and resistance to nucleoside analogues, it seems to be more straightforward and feasible to develop efficient antiviral drugs than to understand

the role of all those proteins encoded in the chimeric domains and the R2 segment, which may have immunologically decisive effects on conferring protection against EHD upon immunization.

Acknowledgments

Formatted: Line spacing: Double

We are grateful to the zoos in Zurich, Basel, and Hamburg for their contributions. Equally, Dr. Monika Hilbe and Prof. Dr. Franco Guscetti, Institute of Veterinary Pathology, University of Zurich are greatly acknowledged for providing necropsy tissue. We also thank Prof. Dr. Cornel Fraefel for his constant support of the viremia screening and antibody testing. ~~Finally, we thank Andreina Schramm for sharing her EHV1 positive horse reference sera.~~ We thank Dr. Andreina Schramm for sharing her EHV1 positive horse reference sera. We also acknowledge the direct collaboration and assistance of Erin Latimer at the Elephant Herpesvirus Laboratory of the Smithsonian National Zoo and J.-C. Zong of the Viral Oncology Program at Johns Hopkins School of Medicine for much of the unpublished sub-genomic PCR sequencing analysis work carried out on earlier EHD cases collected worldwide that was drawn upon from GenBank accession files for the strain differences analyses provided here in Supplementary Material Tables S1 and S2.

References

Formatted: Line spacing: Double

1. Long SY, Latimer EM, Hayward GS. Review of Elephant Endotheliotropic Herpesviruses and Acute Hemorrhagic Disease. ILAR J. 2016;56(3):283-96.
2. Hoornweg TE, Schaftenaar W, Maurer G, van den Doel PB, Molenaar FM, Chamouard-Galante A, et al. Elephant Endotheliotropic Herpesvirus Is Omnipresent in Elephants in European Zoos and an Asian Elephant Range Country. Viruses. 2021;13(2).
3. Zachariah A, Zong JC, Long SY, Latimer EM, Heaggans SY, Richman LK, et al. Fatal herpesvirus hemorrhagic disease in wild and orphan asian elephants in southern India. J Wildl Dis. 2013;49(2):381-93.
4. Richman LK, Zong JC, Latimer EM, Lock J, Fleischer RC, Heaggans SY, et al. Elephant endotheliotropic herpesviruses EEHV1A, EEHV1B, and EEHV2 from cases of hemorrhagic

disease are highly diverged from other mammalian herpesviruses and may form a new subfamily. J Virol. 2014;88(23):13523-46.

5. Ling PD, Long SY, Zong JC, Heaggans SY, Qin X, Hayward GS. Comparison of the Gene Coding Contents and Other Unusual Features of the GC-Rich and AT-Rich Branch Probosciviruses. mSphere. 2016;1(3).

6. Pellett PE. Trunkloads of viruses. J Virol. 2014;88(23):13520-2.

7. Fuery A, Pursell T, Tan J, Peng R, Burbelo PD, Hayward GS, et al. Lethal Hemorrhagic Disease and Clinical Illness Associated with Elephant Endotheliotropic Herpesvirus 1 Are Caused by Primary Infection: Implications for the Detection of Diagnostic Proteins. J Virol. 2020;94(3).

8. Latimer E, Zong JC, Heaggans SY, Richman LK, Hayward GS. Detection and evaluation of novel herpesviruses in routine and pathological samples from Asian and African elephants: identification of two new probosciviruses (EEHV5 and EEHV6) and two new gammaherpesviruses (EGHV3B and EGHV5). Vet Microbiol. 2011;147(1-2):28-41.

9. Zong JC, Heaggans SY, Long SY, Latimer EM, Nofs SA, Bronson E, et al. Detection of Quiescent Infections with Multiple Elephant Endotheliotropic Herpesviruses (EEHVs), Including EEHV2, EEHV3, EEHV6, and EEHV7, within Lymphoid Lung Nodules or Lung and Spleen Tissue Samples from Five Asymptomatic Adult African Elephants. J Virol. 2015;90(6):3028-43.

10. Zong JC, Latimer EM, Long SY, Richman LK, Heaggans SY, Hayward GS. Comparative genome analysis of four elephant endotheliotropic herpesviruses, EEHV3, EEHV4, EEHV5, and EEHV6, from cases of hemorrhagic disease or viremia. J Virol. 2014;88(23):13547-69.

~~10~~11.~~ Wilkie GS, Davison AJ, Watson M, Kerr K, Sanderson S, Bouts T, et al. Complete genome sequences of elephant endotheliotropic herpesviruses 1A and 1B determined directly from fatal cases. J Virol. 2013;87(12):6700-12.~~

~~11. Ling PD, Reid JG, Qin X, Muzny DM, Gibbs R, Petrosino J, et al. Complete Genome Sequence of Elephant Endotheliotropic Herpesvirus 1A. Genome Announc. 2013;1(2):e0010613.~~

~~12~~. Stanton JJ, Zong JC, Latimer E, Tan J, Herron A, Hayward GS, et al. Detection of pathogenic elephant endotheliotropic herpesvirus in routine trunk washes from healthy adult Asian elephants (*Elephas maximus*) by use of a real-time quantitative polymerase chain reaction assay. Am J Vet Res. 2010;71(8):925-33.

~~13~~12. Stanton JJ, Zong JC, Eng C, Howard L, Flanagan J, Stevens M, et al. Kinetics of viral loads and genotypic analysis of elephant endotheliotropic herpesvirus-1 infection in captive Asian elephants (*Elephas maximus*). J Zoo Wildl Med. 2013;44(1):42-54.

~~14~~13. Hoornweg TE, Perera VP, Karunarathne RNS, Schaftenaar W, Mahakapuge TAN, Kalupahana AW, et al. Young elephants in a large herd maintain high levels of elephant endotheliotropic herpesvirus-specific antibodies and do not succumb to fatal haemorrhagic disease. Transbound Emerg Dis. 2022;69(5):e3379-e85.

~~15~~14. Pursell T, Spencer Clinton JL, Tan J, Peng R, Qin X, Doddapaneni H, et al. Primary Infection May Be an Underlying Factor Contributing to Lethal Hemorrhagic Disease Caused by Elephant Endotheliotropic Herpesvirus 3 in African Elephants (*Loxodonta africana*). Microbiol Spectr. 2021;9(2):e0098321.

~~16~~15. Fuery A, Tan J, Peng R, Flanagan JP, Tocidlowski ME, Howard LL, et al. Clinical Infection of Two Captive Asian Elephants (*Elephas Maximus*) with Elephant Endotheliotropic Herpesvirus 1b. J Zoo Wildl Med. 2016;47(1):319-24.

Formatted: Font: Italic

Formatted: Font: Italic

Formatted: Font: Italic

Formatted: Font: Italic

~~17~~16. Ehlers B, Dural G, Marschall M, Schregel V, Goltz M, Hentschke J. Endotheliotropic elephant herpesvirus, the first betaherpesvirus with a thymidine kinase gene. J Gen Virol. 2006;87(Pt 10):2781-9.

~~18~~17. Wilkie GS, Davison AJ, Watson M, Kerr K, Sanderson S, Bouts T, et al. Complete genome sequences of elephant endotheliotropic herpesviruses 1A and 1B determined directly from fatal cases. J Virol. 2013;87(12):6700-12.

18. Ling PD, Reid JG, Qin X, Muzny DM, Gibbs R, Petrosino J, et al. Complete Genome Sequence of Elephant Endotheliotropic Herpesvirus 1A. Genome Announc. 2013;1(2):e0010613.

19. Ling PD, Long SY, Fuery A, Peng RS, Heaggans SY, Qin X, et al. Complete Genome Sequence of Elephant Endotheliotropic Herpesvirus 4, the First Example of a GC-Rich Branch Proboscivirus. mSphere. 2016;1(3).

~~19~~20. Atkins L, Zong JC, Tan J, Mejia A, Heaggans SY, Nofs SA, et al. Elephant endotheliotropic herpesvirus 5, a newly recognized elephant herpesvirus associated with clinical and subclinical infections in captive Asian elephants (*Elephas maximus*). J Zoo Wildl Med. 2013;44(1):136-43.

~~20~~21. Dastjerdi A, Seilern-Moy K, Darpel K, Steinbach F, Molenaar F. Surviving and fatal Elephant Endotheliotropic Herpesvirus-1A infections in juvenile Asian elephants - lessons learned and recommendations on anti-herpesviral therapy. BMC Vet Res. 2016;12(1):178.

~~21~~22. Brock AP, Isaza R, Hunter RP, Richman LK, Montali RJ, Schmitt DL, et al. Estimates of the pharmacokinetics of famciclovir and its active metabolite penciclovir in young Asian elephants (*Elephas maximus*). Am J Vet Res. 2012;73(12):1996-2000.

~~22~~23. Schmitt DL, Hardy DA, Montali RJ, Richman LK, Lindsay WA, Isaza R, et al. Use of famciclovir for the treatment of endotheliotrophic herpesvirus infections in Asian elephants (*Elephas maximus*). J Zoo Wildl Med. 2000;31(4):518-22.

~~23~~24. Wissink-Argilaga N, Dastjerdi A, Molenaar FM. Using in-House Hematology to Direct Decision-Making in the Successful Treatment and Monitoring of a Clinical and Subsequently Subclinical Case of Elephant Endotheliotropic Herpesvirus 1b. J Zoo Wildl Med. 2019;50(2):498-502.

~~24~~25. Ackermann M, Hatt JM, Schetle N, Steinmetz H. Identification of shedders of elephant endotheliotropic herpesviruses among Asian elephants (*Elephas maximus*) in Switzerland. PLoS One. 2017;12(5):e0176891.

~~25~~26. Ackermann M, Hatt J-M. To treat or not to treat? Veterinary Record Case Reports. 2019;7(3):e000847.

~~26~~27. Ossent P, Guscetti F, Metzler AE, Lang EM, Rubel A, Hauser B. Acute and fatal herpesvirus infection in a young Asian elephant (*Elephas maximus*). Vet Pathol. 1990;27(2):131-3.

~~27~~28~~. Burbelo PD, Ching KH, Klimavicz CM, Iadarola MJ. Antibody profiling by Luciferase Immunoprecipitation Systems (LIPS). J Vis Exp. 2009(32).~~

~~28. Kubacki J, Fraefel C, Bachofen C. Implementation of next-generation sequencing for virus identification in veterinary diagnostic laboratories. J Vet Diagn Invest. 2021;33(2):235-47.~~

~~29. Kubacki J, Flacio E, Qi W, Guidi V, Tonolla M, Fraefel C. Viral Metagenomic Analysis of Aedes albopictus Mosquitos from Southern Switzerland. Viruses. 2020;12(9).~~

~~30. Bolger AM, Lohse M, Usadel B. Trimmomatic: a flexible trimmer for Illumina sequence data. Bioinformatics. 2014;30(15):2114-20.~~

~~31. Nurk S, Meleshko D, Korobeynikov A, Pevzner PA. metaSPAdes: a new versatile metagenomic assembler. Genome Res. 2017;27(5):824-34.~~

Formatted: Font: Italic

Formatted: Font: Italic

Formatted: Font: Italic

Formatted: Font: Italic

Formatted: Font: Italic

Formatted: German (Switzerland)

~~32. Altschul SF, Gish W, Miller W, Myers EW, Lipman DJ. Basic local alignment search tool. J Mol Biol. 1990;215(3):403-10.~~
~~33~~. Zachariah A, Sajesh PK, Santhosh S, Bathrachalam C, Megha M, Pandiyan J, et al. Extended genotypic evaluation and comparison of twenty-two cases of lethal EEHV1 hemorrhagic disease in wild and captive Asian elephants in India. PLoS One. 2018;13(8):e0202438.
~~34~~29. Oo ZM, Aung YH, Aung TT, San N, Tun ZM, Hayward GS, et al. Elephant Endotheliotropic Herpesvirus Hemorrhagic Disease in Asian Elephant Calves in Logging Camps, Myanmar. Emerg Infect Dis. 2020;26(1):63-9.
30. Rubel A, Zingg R. Kaeng Krachan Elephant Park for Asian elephants (*Elephas maximus Linnaeus*, 1758) at Zurich Zoo. (Kaeng Krachan Elefantenpark fuer Asiatische Elefanten (*Elephas maximus Linnaeus*, 1758) im Zoo Zuerich.) Zoologische Garten. 2015;84(1-2):1-12.
31. Burbelo PD, Ching KH, Klimavicz CM, Iadarola MJ. Antibody profiling by Luciferase Immunoprecipitation Systems (LIPS). J Vis Exp. 2009(32).
32. Work TM, Dagenais J, Balazs GH, Schettle N, Ackermann M. Dynamics of Virus Shedding and In Situ Confirmation of Chelonid Herpesvirus 5 in Hawaiian Green Turtles With Fibropapillomatosis. Vet Pathol. 2015;52(6):1195-201.
33. Kubacki J, Fraefel C, Bachofen C. Implementation of next-generation sequencing for virus identification in veterinary diagnostic laboratories. J Vet Diagn Invest. 2021;33(2):235-47.
34. Kubacki J, Flacio E, Qi W, Guidi V, Tonolla M, Fraefel C. Viral Metagenomic Analysis of Aedes albopictus Mosquitos from Southern Switzerland. Viruses. 2020;12(9).
35. Bolger AM, Lohse M, Usadel B. Trimmomatic: a flexible trimmer for Illumina sequence data. Bioinformatics. 2014;30(15):2114-20.
36. Nurk S, Meleshko D, Korobeynikov A, Pevzner PA. metaSPAdes: a new versatile metagenomic assembler. Genome Res. 2017;27(5):824-34.
37. Altschul SF, Gish W, Miller W, Myers EW, Lipman DJ. Basic local alignment search tool. J Mol Biol. 1990;215(3):403-10.
38. Moraru C, Varsani A, Kropinski AM. VIRIDIC-A Novel Tool to Calculate the Intergenomic Similarities of Prokaryote-Infecting Viruses. Viruses. 2020;12(11).
39. Dominguez G, Dambaugh TR, Stamey FR, Dewhurst S, Inoue N, Pellett PE. Human herpesvirus 6B genome sequence: coding content and comparison with human herpesvirus 6A. J Virol. 1999;73(10):8040-52.
40. Burrel S, Deback C, Agut H, Boutolleau D. Genotypic characterization of UL23 thymidine kinase and UL30 DNA polymerase of clinical isolates of herpes simplex virus: natural polymorphism and mutations associated with resistance to antivirals. Antimicrob Agents Chemother. 2010;54(11):4833-42.
41. Komatsu TE, Pikis A, Naeger LK, Harrington PR. Resistance of human cytomegalovirus to ganciclovir/valganciclovir: a comprehensive review of putative resistance pathways. Antiviral Res. 2014;101:12-25.
42. Topalis D, Gillemot S, Snoeck R, Andrei G. Thymidine kinase and protein kinase in drug-resistant herpesviruses: Heads of a Lernaean Hydra. Drug Resist Updat. 2018;37:1-16.
43. Stremme C, Priadi A, Hayward GS, Zachariah A. Identification of Two Lethal Cases of Elephant Endotheliotropic Herpesvirus Hemorrhagic Disease in Sumatran Elephant Calves in Indonesia. J Zoo Wildl Med. 2021;51(4):985-93.
44. Lee MH, Nathan S, Benedict L, Nagalingam P, Latimer E, Hughes T, et al. The first reported cases of elephant endotheliotropic herpesvirus infectious haemorrhagic disease in Malaysia: case report. Virol J. 2021;18(1):231.

Formatted: German (Switzerland)

Supporting information

~~S1~~ **Table** S1. EEHV1A (Umesh~~, EP55~~) Variable Gene Subtype Comparisons.

~~+ = Inserted alternative gene casettes that do not display subtyping.~~

~~– = Absence of any inserted gene casettes at these loci.~~

~~Del, fr or fs = deleted, fragmented or frame-shifted protein versions.~~

~~O = Intact E54 proteins which display up to 15% variability at the aa level, but without any clustal subtyping patterns.~~

~~* E37(ORF-O), E38 (ORF-P) and E39 (ORF-Q) proteins are also part of the CD-III chimeric domain.~~

~~# Just four of the eight known alternative inserted three-gene cassette groups (A, B, D and F) in the R2-segment were selected for inclusion here (from a total of 30 EEHV1 strains examined).~~

Table S2. Details about the Ten EEHV1 Genomes Used in Table S1.

Formatted: English (United States)

Formatted: Normal

Formatted: Font: Not Bold, English (United States)

Formatted: Font: Not Bold, English (United States)

Formatted: English (United States)

Formatted: Font: Bold

Rebuttal Letter PONE-D-24-09462

First, we would like to thank the reviewers for their careful and considerate work, providing together a total of 227 comments.
As reviewer #2 provided "only" 40 comments, we chose to address his issues first, followed by addressing the 187 issues raised by reviewer #1.

**Reviewer #2**	**Our Response**
	Line references correspond to the reviewer's own wording
L30: "This virus probably re-emerged" (it is not a "new" virus")	Agreed: "new" replaced by "newly identified"
L73: "knowledge of …is also important"	Corrected ("are" replaced by "is")
L80. Define "ECPK"	the EEHV homologue to CPK (ECPK)
L85: delete "at least"	Done
L103: "to admit"	"admitting" replaced by "admit"
L183-186. A real-time PCR assay was used to obtain relative quantitation of virus and host genome copy numbers. The virus target is present at one copy per virus genome, and the host target is present at two copies per diploid genome. The text should be modified to make this clear, as well as its influence on data interpretation.	Text adapted accordingly
L315. Is "at least" needed here?	"at least" deleted
L403. I do not know the meaning of "27'488 and 4679 NNNs".	Text adjusted
L405. A denominator is needed for the 56 nt mismatch.	Text adjusted
(comments L417-LK420)	Indeed, we have reorganized and rewritten the entire section (original L408 to 443)
L417. Not sure what is meant by "non-core" likely tegument protein	Deleted in the course of the section's reorganization
L420. Roseoloviruses also encode OBPs	"unique" replaced by "unusual". We are not trying to point out all resemblances or differences here – just to HCMV
The L417 and L420 items end up being mentioned in lines 431-434, but those areas might be able to be more tightly integrated.	Some rewriting has been carried out here and 25 lines from this section have been moved to DNA Annotations section of M&M (as request by Reviewer #1)
L448. Is the hypervariability within isolates or strains, or between isolates or strains?	No, it is between strains not within strains. "between them" has been added after "genetic divergence and associated with".
The complex string spanning **lines 509-513** contains what seem to be inconsistencies in usage of commas, semicolons, periods, and parentheses	This whole section was removed.

L528. p. 22. Not sure what is meant by “triplex patterns”, which are mentioned here and on p. 22 line 528.	Triplex refers to the inserted alternative three gene cassettes. Within the R2 segment. Re-phrased as “recognize 13 different three gene cassette (triplex) patterns ...and on L583 as ..typical group-D E59/60/61 triplet cassettes....
L642. “routing” to “rooting”	Done
L670. “sequence of Kimba contained”	Done
L672. “address their biological function in the future	Corrected
L675. delete “considered”	Done
L676-677. “enzyme, thereby terminating its activity, or”	Corrected
L682. I don’t understand the sentence that begins with “Interestingly”	Clarified as follows: Interestingly, Leu792 in our sequence compared to Isoleucine at this position in all ten previously published isolates (L792I). This seeming mutation mapped to the center of a conserved domain, ….
L695. delete “single one”	Done
Having defined its abbreviation in line 702, use “gB” thereafter.	Done
L713. “of gB subtype 1B”	Corrected
L728. “revealed much” to “detected”	Done
L762. Is “apparently” needed?	“apparently” deleted
L778. “homology” to “similarity”	Done
L786. “new” to “previously undescribed”	Done
L792. “convey information about”	Corrected
L793. “If all” to “Since”	Done
L802. “Despite the long”	“of” deleted
Lines 805, 807, and 809 say the same thing three different ways, which is confusing. “U38 DNA polymerase”, “U38 protein”, and U38<br>DNA pol. Pick a winner.	Done as follows: … the U38 DNA polymerase may be naturally incompetent to incorporate certain phosphorylated nucleoside analogues into the growing viral DNA. Due to the lack of suitable cell cultures, this issue has not yet been addressed. Still, an interesting variation was observed within the U38 DNA polymerase sequence, where a single mutation (L729I) in all our isolates opposed …
1. It seems like Umesh’s sequence should be included in Table 2.	Done
2. In Fig. 1, the orange used for Thai, is too similar to Indi’s yellow for the parentage of Umesh to be unambiguous. A similar situation exists for Ruwani. It would be helpful to indicate which animals died from	Figure revised accordingly

EHD. Are the two prior cases those of Xian and Aishu? If so, please say so.	
3. Parts of the case descriptions are difficult to follow because of the several nomenclatures used. Lines 382-390 provide several illustrations of this:<br>• In line 382, "EP07" is used as a name for an animal. In line 389, "EP55" is described as the name of an isolate. In Table 2, "EP55" is described as a "Case". From context, I wondered whether similar designations connected to Genbank entries. Too much work is required to decipher the information. It would be helpful to adopt a consistent scheme for such things.<br>• It would also be helpful to include a comprehensive expanded version of Table 2 that includes all of the animals mentioned in the paper, their birth and death years, virus and strain information, sequence accessions, lengths, and genes (for short sequences), etc.	We agree. Using the names of the animals is helpful to understand, where and how the animals were bred and kept and how various cases interrelate. Importantly, most of the complete EEHV genomes available in public databases are also named after animals. Moreover, much of these "personal" data are publicly available on EleWiki. Therefore, we decided to use predominantly elephant names in our paper, but we use Table 2 to clarify alternative naming such as case numbers and GenBank entries.<br><br>Background info and acc nos for the ten genomes used in Supplemental Table S1 are also now similarly listed in the new supplementary Table S2
4. A single, large "EEHV1 Loads" could be added parallel to the y-axes on the left side of Fig. 3	We prefer using the term "ΔCt", which is what we measured.
5. Fig. 4 would be easier to understand if it included the names of the animals, in place of, or at least in addition to codes such as "E361A_EP07".	Done
6. Fig. 5 would be improved by addition of animal names and where they are from (American, Zurich, or European).	Animal names included. Origin explained in the caption
7. Fig. 6 would be improved by a lighter color scheme in the upper right half and a larger font in the lower left half. The color scheme for the aligned genome fraction provides little information due to the high degree of similarity across the collection. A color gradient that ranges from 80% or 90% to 100% might be more informative.	Done
8. The red text in Figure 7 is illegible. A larger font will increase the vertical dimension of the figure, but that is OK.	The text is readable (or can be enlarged to the effect) in the 300 dpi version of the Figure

**Reviewer #1**	**Our Response**
	Line references correspond to the reviewer's pdf file

The results section does not clearly match this title	Part of the wording of the manuscript title has been changed from “Virological Analysis” to “Viral Genomic Analysis” to cover the extraordinarily large amount of overall protein divergence that is now being recognized here across the full length of intact genomes of multiple EEHV1A strains and EHD cases worldwide.
In **the** summer **of** 2022	Done (both title page and Abstract on page 9)
“*Elephas maximus*” in Italics	The term is actually in Italics in the Abstract of our word document and pdf version (page 9), but Italics were lost upon transfer of the Abstract to the title page and assembly of pdf files by PLOS One. We shall keep an eye on that issue during re-submission and publication. Thank you.
“The conclusions from these analyses may be summarized in three points” replace by “The results show”	Done (both title page and Abstract on page 9)
“In particular, ways need to be found to circumvent the present unavailability of appropriate cell cultures and animal models.” (Title page) This sentence does not reflect the topic researched and should therefore be deleted. (Page 9, lines 34-35) This is a stand alone opinion that is not in the discussion or in the research topic, so suggest to delete	Done (both on title page and Abstract on page 9)
Page 2 of the assembled pdf “donation from the late Prof. Dr. Robert Wyler”	Done. In addition, “Finanical disclosure” was revised to include financial support for the Johns Hopkins University Investigators
Line 35. “circumvent” this is a strange word choice.	Entire sentence deleted
Line 44. (EEHV1B),	Semicolon added. Thank you.
Line 45. “serologic**al** level”	Corrected as suggested
Line 50. Include reference	The case had been presented at the EEHV workshop 2022. Unfortunately, the corresponding author did not reply to our several requests to give us permission to cite their case. Therefore, we decided to modify the text, citing a more general paper.
Line 51. Use either African elephants or *L. africana*, but not sometimes one and sometimes the other. The Latin name is only required once at the start of the paper, straight after the common name.	Corrected. Thank you.
Line 52. (same comment as above)	Same correction. Thank you.

Line 56. “no other susceptible species have been identified”	Done. Thank you.
Line 57. Try to avoid emotive words throughout the paper, such as 'suprisingly'. A better sentence: Not until recently was the full EEHV genomic sequence identified	Point taken. Sentence adjusted in our own words.
Line 60-62. Delete “for poorly understood reasons,” from “All known EEHV strains replicate primarily in the respiratory tract but, for poorly understood reasons, can cause viremia, which is the major precondition for developing EHD.” Instead complete the sentence with “...EHD, and the pathogenesis is poorly understood.”	We disagree. The suggested wording changes the message of our argument. Replication in the respiratory tract serves the purpose of virus excretion and transmission. In contrast, the viremic phase gives no obvious advantage to the virus’s life cycle. Therefore, we worded “for poorly understood reasons”. We re-worded our text to make it easier to understand.
Line 63. Delete “is associated to”	Done
Line 64. ....antibodies. During this period, subclinical infections.....	Agreed. Thank you.
Line 65. ...place, which induces active.....	Agreed. Thank you.
Line 66. ...later in life.	Agreed. Thank you.
Line 67. ...EDH during and right after weaning (2-8y), in association with the waning of maternal antibodies.	Agreed. Thank you.
Line 70. Replace “quite” by “usually”	“Quite” deleted without replacement
Line 72. In the same zoo?	Yes
Line 73. Insert reference	Unfortunately, this story has not been published. To compensate, we inserted individual GenBank references.
Line 73. Delete “However”	Done
Line 85. Delete “at least”	Done
Line 92. Delete “including ours”	Done
Line 93. ....strains, and to	Done
Line 103 ff. Please describe the entire herd and husbandry without emotive words and anonimise the names for ease of reading. E.g. A herd is made up of two males and seven females (2.7), then you can give the ages groups etc. the cases you describe should be Case 1A, 1B, 1C, for example. You should give details on these in the text, e.g. age at the time of the incident you are describing. The rest of the herd can be described impersonal as well with details in an appendix: e.g. 'breeding bull'. If its important that he had newly arrived, this can be mentioned in the introduction but	**We disagree** for the following reasons: Most of the complete EEHV genomes available are named after animals. Due to the website EleWiki the “personal” details of the elephants, including their cause of death, are publicly available (https://www.google.com/url?sa=t&source=web&rct=j&opi=89978449&url=https://elefanten.fandom.com/wiki/Hauptseite&ved=2ahUKEwjOvl-orO-HAxWUgv0HHSY6AOYQFnoECB0QAQ&usg=AOvVaw2Ss-w-RLV5SEX_JQsiDlGY) Naming the animals is also helpful to track previous as well as further cases, to appreciate how the animals were kept and

this fact does not need to be mentioned in the discussion again.<br>If the family group is clinically important, call them Group I and Group II or similar, with their origin, but steer clear of names.<br>If the actual names of the animals are important for international discussions, put those in an appendix.	bread, and to find information about the correlation between animals.<br>As stated by reviewer 2, we have already a complicated situation due to case numbers (for example EP55) and GenBank entry numbers, which refer to the same animal but neither of them is available for unaffected animals. Using just animal names throughout the text will make reading much easier. Anyway, EleWiki makes names and backgrounds publicly available.
Lines 103-104. This is called a 'closed collection'. Rephrase to use these proper phrases - it makes the text more succinct and clear	Rephrased
Line 104. Report actual age	Done
Line 104. Replace “bull” with “male”	Done
Line 104. A new breeding bull was **introduced** to the herd....	… a 10-year-old male was introduced to the herd …
Lines 105-106. Delete – irrelevant	We disagree, these were the exact reasons to acquire a new breeder from outside
Line 106. There were xxx juveniles at risk in the herd (ages)	In 2014, it was exactly the one animal. Yet, we rephrased the text to note the advent of two additional calves (2017 and 2020, respectively) and their integration into preventive surveillance.
Lines 106-107. Delete “it seemed appropriate”	Done
Lines 107 ff. To protect at-risk herd members, a EEHV preventative protocol was instigated, which included	Rephrased
Line 110. Replace “Thai” with “new breeding bull”	We disagree with the arguments discussed above.
Lines 111-112. by EEHV 1 and EEHV 4 PCR	Done
Lines 112-113. “Serology was established “en route” as it became available from literature”<br>delete this sentence - it does not mean much.<br>State clearly when serological samples were collected from	We disagree. Our screening program evolved over time, in particular as it concerns serology. Reports about valuable ELISAs and LIPS assays and their proper interpretation became only available between 2020 and 2022 (as we cite properly).
Lines 113-114. Replace present text with “A EEHV reactive protocol was additionally in place, which included stocking of gancyclovir and famcyclovir for treatment of clinical cases.”	Rephrased
Lines 115-120. Replace present text with “Eight years later, three juveniles elephants	Rephrased as suggested

from this herd died of EHV caused by a previously undeteced EEHV1A strain. This paper describes the attempts at identification of the shoudre of this outbreak through serological surveyance.”	
Line 115. Please review your entire paper and remove all emotive expressions	Done
Lines 122 ff (Materials and Methods):<br>1) describe herd and give all cases a number for ease of reading. Do not use animal names throughout the paper. Include details on the described cases in this paper (3 cases), put all other details in an appendix or as supplemental material	The reviewer requested already in Line 103 to refrain from using animal names. Here, even the introduction of a case number is requested.<br>However, Reviewer 2 criticizes in his issue #3 that “*Parts of the case descriptions are difficult to follow because of the several nomenclatures used*.” Indeed, each elephant has its own name but only animals that succumbed to EHD have a case number, for example EP55 in the case of the present case Umesh. Even cases of which no GenBank entries exist, may have been provided with a case number, for example EP16 Aishu. Importantly, Genbank entries of full EEHV genomes provide most often elephant names, for example the American prototype EEHV1A “Kimba” and the European prototypes EEHV1A “Raman” and EEHV1B “Emelia”.<br>As “personal” information about many elephants (including all individuals from our zoo), is publicly available on Elewiki (for example https://elefanten.fandom.com/wiki/Umesh), the introduction of anonymizing case numbers for the sake of this paper would only complicate matters. We feel that this type of transparent information is required to gain and spread scientific information about EHD. Consequently, we suggest to use the elephant’s names consistently throughout this paper.
2) clearly describe the husbandry setting in this section, including access, frequency of testing, methods of sampling etc	Description of husbandry setting included and referenced. Sampling frequency clarified.
3) put all methods in this section, including thought process on why certain methods were used. Remove method descriptions from the results section	We agree to a certain extent, having adjusted M+M accordingly. However, the purpose of the experiment is at times very useful, just prior to describing the observation in the Results section.

Line 144. If the family tree is important, please explain so in the discussion	Arguments used in the revised discussion.
Lines 153-154. Were these used as controls?<br>Your title implies that you are only looking at samples from Switzerland, but you have also analysed samples from German cases. This is confusing. Please consider how these samples influenced your thought process and include rationale on why these samples were analysed for this study	Yes, these were made available to us as positive controls (positive in terms of: this sample comes from an elephant, who succumbed to EHD). Text adjusted accordingly.
Line 159. Serum samples from four individual African elephants from a separate Swiss zoo were collected that same year.	As the sera were collected throughout January, why not state the fact?
Line 162. acknowledgements, not main text body	Agree
Line 164. “*and minced in a combination of 300 µL of body fluid and 300 µL blood*” replace “in a combination of” by “with”	Done
Line 167. acknowledgements, not main text body	Done
Line 171. TNF-alpha?	Yes, added to the revised manuscript. Thank you
Line 178. reference for cut off value	The decision was not made on previously published results, but it is an arbitrary but important factor for interpreting our data, in particular since we used diluted blood droplets as starting material.
Line 190. The first day of viraemia of your first case (index case) is Day 0.<br>From Day 0, time all the other cases. descriptors such as 'very early' are for the discussion	Probably, we did not state it clearly enough, but it was indeed our intention to sub characterize the causative virus as early as possible because this knowledge had the potential to influence our therapeutic options. For example, if the virus had been identified as subtype 1B, we might have had the option to use Omysha’s serum (the elephant who had been identified as shedding subtype 1B prior to the outbreak) for treating Umesh. Similarly, if the virus had been closely related to the previous Xian isolate, we might have had the option to use the serum of one of the animals that had been there at the time.<br>While the reasons behind the decision are better placed in the discussion, the fact “early” clearly belongs here into the M+M section.

Line 191. PCR technique was	We prefer “*parameters*” because more than bare technical aspects are affected. (“*conditions*” replaced accordingly)
Line 207. Please include published reference	Done
Line 208. (GenScripts Europe, the Netherlands).	Replaced according to your suggestion
Line 224. add address of manufacturer	Done
Line 234. These were kept in	Replaced
Line 237. With -> by	Replaced
Line 240. manufacturers address	Inserted
Line 241. 8 -> eight	Done
Line 249. Insert reference	Done, including explanation
Line 252. Delete “*Eventually*”	Done
Line 256. What was sheared? The noun is missing from this sentence	The nucleic acids (inserted)
Line 265. italics	Done
Line 267. Subsequently,	Done
Line 270. Italics	Done
Line 270. NCBI: Write out in full the first time used in text	Done
Line 289. The results section is too elaborate and includes text that belongs in materials and methods and some which belongs in the discussion.<br>ONLY list the actual results from your study, in a clear and concise way. Interpretation is for the discussion section. Some parts can be moved to supplemental materials.	Done. The results section largely remains intact, although rewritten and rearranged extensively throughout to maintain an integrated and cohesive section on strain and gene subtyping. However, some text has been moved out to M&M entitled Annotation of EEHV Genomes. In addition, a new section of text covering background and history of EEHV subtyping for the Introduction and another as a summary and comments on EEHV subtyping for the Discussion.
Line 291. Between x date and y date, ...	Done
Lines 293-294. 'tolerated sampling procedure'<br>Too emotive<br>In materials and methods, it should be described that sampling, based on animal access and training, commenced on Day x for case 1B and Day y for case 1C.	The paragraph has been adjusted.
Line 295. 1)How long were the elephants viraemic for?<br>2)How high was the viraemia? Was a qPCR done on positive samples?<br>3)What was the time between viraemic events?<br>4)Did treatment influence viraemia?	Ad 1) not repeatedly, until outbreak.<br>Ad 2) the screening was done by Taqman PCR; ΔCt values were used to estimate the viral load (as described in M+M). The paragraph was re-written to clarify.<br>Ad 3) Very irregular. Details will be seen on our data sheet.

	Ad 4) no effects at all observed.
Line 306. Rationale for using horse serum should be in materials and methods	Done
Line 307/308. If these sera were part of the study, and not published elsewhere, these cases, with different case numbers, should be included in that table.	Unfortunately, specific data about the EHD cases of Kanja and Anjuli have not been published. But blood samples have been made available to us to serve as positive controls (as described in M+M). These sera were, therefore, not part of our study.
Lines 312-313. Discussion sentence	We disagree. We describe an observation.
Lines 313-321. Discussion - this is interpretation - interpreting one result against another. The actual results are not clear from this section.<br>Please list only the results first, of all the samples tested for this study, and include a table of ALL the results. Interpretation can come afterwards, separately	We disagree. The results of all serological assays are combined in Fig. 2. In the text, we point out the reactions of individuals in a descriptive manner (as appropriate for the Results section).
Lines 322-324. Materials and methods	Done. See section “LIPS assay”
Lines 325-326. Is this a result of your tests? If so, include it in the listing of all results plus include in the table, to give the reader an overview.	Yes, the results are presented together with all other serological results in Fig. 2.
Lines 327-328. Discussion	We disagree. It’s summing up all of the serological data in one sentence.
Line 345. This date is irrelevant, the weekday especially.<br>If Day 0 was the first day of viraemia of your index case, which day was this? Day -7?	The weekday may be irrelevant, although screenings were usually done on Thursdays. However, on this day of routine screening, all of the elephants were still aviremic, while exactly one week later, the outbreak was first detected. As the screening took place on a weekly schedule, we believe that this information is important.
Line 346. this is Day 0, presumable.<br>A clear timeline is very important for your reader to interpret and critically assess your findings	As explained above, we do not really know Day 0 or Day 1.
Line 351. Day 3	See above.
Line 352. Day x	See above.
Line 354. Day y	See above.
Line 355. delete<br>This word does not belong in the results section	We disagree.
Line 357. Case 1A, 1B and 1C	Adjusted, individual names used
Lines 365-369. Materials and methods<br>In the results section, only state:<br>Conventional PCR targeting the E36/U79 locus showed	Adjusted

Lines 378-380. Materials and methods	Adjusted
Lines 383-384. materials and methods<br><br>In the results section, only state: Comparison of Case 1A with X and Y showed	Adjusted (names)
Line 406. Rationale for this should be in M&M, not in results	We disagree. This decision was not planned but was a direct consequence of the results described in this section.
Lines 412-427. Abridge and move to discussion	We disagree. The description of the genomic organization is an important major lead-in here to the whole large results section describing multiple separate findings about levels and patterns of divergence found within numerous hypervariable regions within both Umesh itself and many diverged reference genomes.
Lines 428-430. (Highlighted but no written comment)	?? (probably part of the previous and next comment?)
Lines 431-443. Abridge and move to materials and methods	This section has been moved to M&M as suggested under the new subtitle of Annotation of EEHV Genomes.
Lines 446-452. Introduction?	Whilst it could be used in that way, there is already a new section in the Introduction covering earlier work on some aspects of strain variability in the Introduction, whereas, this section remains as a necessary lead-in to the major new results section immediately following this about detailed qualitative and sometimes quantitative differences from other complete EEHV genomes both globally (Fig 6) as well as at key variable gene loci.
Lines 465-466. M&M	Thank you, see new Chapter "Annotation of EEHV genomes" in M+M
Line 468. Delete *„it is noticeable that"*	Changed to "Furthermore, "
Lines 470-476. interpretations<br>Abridge please, the results section is not clear	Lots of rewriting here, with parts also moved to Introduction.
Lines 477-481. M&M	Now omitted
Lines 488-498. relevance to title of the paper is not clear. Supplmental materials?	The previous detailed comparisons to EEHV1B have been largely omitted, with some of the rest moved to M&M. The title of the paper has been updated to include "Viral Genomics" and fully integrate the gene subtyping results with the rest of the text as a major part of the manuscript
Line 498. Delete *"finally"*	Entire section deleted

Lines 505-513. Delete 6 highlighted expressions	Done in the course of other changes in the text
Line 513. Replace “*of this information for*”by “*of Case 1A*”	Disagree, name still used (see elsewhere)
Lines 516-556. Distill the actual result and provide the full explanation in supplemental materials	Both Table S1 and the new added Table S2 are now given as Supplemental Materials accompanying the text for the overall Gene Subtyping results and comparisons sections presented as a coherent.
Lines 559-569. Distill any results and move the explanation to supplemental materials	Same as for 516-575 but considerably rephrased and reorganized as a single cohesive section under the subheadings of Comparison with main segment EEHV1 variable gene subtype arrangements and Comparison with other EEHV1 R2 segment arrangements.
Lines 570-575. M&M?	Same as for 516-575 but considerably rephrased and reorganized as a single cohesive section as above
Lines 581-598. This section is very verbose and the result, and its relevance to the title of the paper, are not clear	The title of the paper has been updated to include “Viral Genomics” covering the now fully integrated gene subtyping results within the main results text as a major part of the manuscript
Lines 604-610. supplemental materials	We disagree. This background info is best kept together with the rest of the clarifying info re overall Umesh R2 segment subgrouping results for different strains given in Supplementary Table S1.
Lines 611-615. Discussion	Moved
Lines 615-620. Not a result - move elsewhere	Moved to end of Annotation section in M&M.
Lines 620-621. Discussion	We disagree. It would be weird to isolate this brief comment from its overall context just above.
Lines 624-626. Repetition	Serves as a necessary linker statement between the end of the extensive Umesh versus other strain difference comparison results above and the switch to focus on targeted questions about potential pharmacological inhibitor issues.
Lines 633-634. Introduction	Again, serves as a lead-in to a new subsection.
Lines 635-636. Delete	Serves as part of a brief preamble to the new enzymatic assessment section.
Lines 643-644. Delete	No, retained as a relevant follow-up comment that would be lost if moved to another section¨

Lines 644-646. This can be the first line of your discussion	We do not want to separate this from being a summary of the previous extensive results in front of it.
Lines 654-656. Materials & methods	Retained = preamble to results
Line 656. Delete *"Indeed, "*	Done
Line 657. Replace "*displaying just*" by ",.... *with only one*...."	Done
Lines 660-662. Discussion	No, retained as a relevant adjunct comment on the result
Lines 665-666. Delete several words	Done
Lines 671-672. Discussion	Difference from Umesh = Part of the results
Lines 678-679. M&M	It's also the rationale and preamble for the present experiment
Line 679. Delete *"Notably,"*	Done
Lines 685-688. relevance unclear	The entire section has be re-organized for clarification
Line 703. Elephants.	Done
Line 704-706. delete<br>instead: advantage, as it is not species specific.	Done
Line 706. Delete<br>instead: Therefore, horse sera could be used to determine the negative cut off value.	Done
Line 708. Delete (and replace with):<br>All adult elephants had ...	Done
Line 709. Delete *"sera from"*	No, it is needed to make sense
Line 709. Acknowledgements	Deleted from Discussion
Line 710. Delete *"also"*	Done
Line 715. Delete *"the sera from"*	Done
Line 715. (Insert) "...., possibly due to subclinical infection with EEHV6"	Argument rephrased
Line 716. were the samples tested for this? If not, please provide a reference why this assumption was made.	No testing for EEHV6 was done (serology assay not available yet). Almost all adult African elephants have EEHV6 (plus EEHV2 and EEHV3) DNA in saliva, but EEHV6 is the most likely to cross-react with EEHV1 reagents. Text rephrased accordingly.
Line 721. delete.4<br>....may be explained by	Sentence modified accordingly
Lines 725-727. Delete	Rephrased but retained
Line 733. keep names out of the discussion - not clear	Disagree as explained above.
Line 738. short-lived, and therefore young elephants are at risk during an EEHV outbreak.	Rewritten to clarify our opinion

Line 739. reference increased shedding here? You are only discussing viraemia - viraemia does not equate shedding - shedding puts others at risk<br><br>also, treatment of a sick elephant can cause stress of the other juveniles due to routine disruption - that can set off latent/low level viraemia	We agree but viremia in a very young elephant does not occur without prior transmission, due to shedding from an adult. Both shedding and viremia may proceed unnoticed. A more suitable reference was inserted.<br>True, but according to the screening results, apparent stress episodes (for example killing of a newborn by members of the herd, which occurred in Winter 2021) had not evoked any viremic phases. Moreover, EHD involves primary infection not reactivation
Line 741. This should be included or published in tandem - treatment may influence viraemia. If this information cannot be published with this paper, this paper should focus only on identifying the source, and not speculate on onset of EHD, viraemic levels throughout EHD, etc. It is important to know what veterinary interventions were done so interpret the viraemic levels seen.	We agree to a certain extent. However, the zoo declined to publish precise data about the treatment. We know that Umesh was not treated with antivirals prior to June 24th, whereas Omysha was not treated prior to July 5th. Ruwani refused at all to being treated. Obviously, no measurable effect of the drug treatments can be seen.<br>In contrast, measuring the viremic levels was part of our own investigations. Neither these data nor their interpretation should be withheld. The text was modified accordingly.
Line 743. emotive<br>use instead: died on Day x	"succumbed" changed to "died of
Line 743. qPCR results would be useful here. CBC results as well. If none of these can be incorportaed in thsi paper, this paragraph may need to be deleted as speculations are made without proper data to support it.	These results are actually shown in Fig. 3. We inserted the corresponding cross reference into the text.
Lines 756-759. Delete	We disagree. This is an important aspect of the full story here.
Line 761. delete<br>instead:<br>...ruling out a re-emergence of a previously latent strain	Done
Lines 762-763. delete, repetition	We disagree. It is important to state the fact in the context.
Line 782. the breeding bull	Done
Line 783. Case 1B?	As explained above, we have decided to use animal's names, whose background information is presented in Fig. 1
Line 790. Reference	4 references inserted (16, 40-42)
Line 796. Follow on from here with line 814	Disagree. We believe that it makes sense to keep the issues of potential drug resistance

	apart from the potential role in epidemiology
Line 802. Delete highlighted word	Done
Lines 804-805. this is necessary to interpret the results. Refrain from all speculation with regards to effects of treatment on viraemia if this data is not available to you<br><br>consider a tandem publication so that the case becomes clearer as an alternative, or publish as a single paper of more scientific power	Disagree. It is a general and very important issue that needs to be addressed.<br><br>We did consider it. To no avail.
Line 814. connect ot 796	Disagree (as argued in our response to Line 796 comment)
Line 819. M&M	Disagree. This information is important in the context. With the exception of three elephants (Ceyla, Indi, Thai), all members of the herd had been born in our zoo. Therefore, only Ceyla, Indi, and Thai can bring a "new virus" into circulation. Therefore, it is important to discuss, where they came from and when.
Line 824. Any idea what (husbandry) event could have triggered shedding of a latent infection?	Indi, at the age of 38, is getting old. However, reactivation events are always asymptomatic. To our best knowledge, the true triggering factors have not yet been identified.
Line 829. (Replace by) "*Possibly*"	Done
Line 830. by deduction - or do you have firmer evidence? Has this elephant ever shed this strain?	As stated above, Indi and her daughter Chandra were the only members in the herd to have antibodies against ORF-Q-C and, thus, to have encountered the virus prior to the outbreak. Chandra was born in the zoo, while Indi originates from Myanmar, leaving Indi as the most likely original source of the virus. As stated above, occasional asymptomatic periods of shedding go unnoticed.
Line 831. This may be a conclusion if the treatment protocol was incorporated in the paper, which it isn't	Disagree. This general knowledge is reconfirmed by the present case. It is also a very significant point that the reader needs to be reminded about
Line 832. delete<br>This is not in the discussion and not in the results	But it is generally known and influenced preventive measures as well treatment options
Lines 840 ff. (References section) Please put all latin names in italics	Agree. The format became lost during upload of the manuscript.
