# Supplementary material for: Epidemiological, serological, and viral genomic analysis of an outbreak of elephant hemorrhagic disease in Switzerland
Source: PLoS One. 2025 Apr 7;20(4):e0301247. doi: 10.1371/journal.pone.0301247 (PMC11975098; doi:10.1371/journal.pone.0301247)
Supplement: Attachment — Submitted filename: Rebuttal Table.docx [file pone.0301247.s005.docx]

Rebuttal Letter PONE-D-24-09462

First, we would like to thank the reviewers for their careful and considerate work, providing together a total of 227 comments.

As reviewer #2 provided “only” 40 comments, we chose to address his issues first, followed by addressing the 187 issues raised by reviewer #1.

| **Reviewer #2** | **Our Response** |
| --- | --- |
|  | Line references correspond to the reviewer’s own wording |
| L30: “This virus probably re-emerged” (it is not a “new” virus”) | Agreed: “new” replaced by “newly identified” |
| L73: “knowledge of …is also important” | Corrected (“are” replaced by “is”) |
| L80. Define “ECPK” | the EEHV homologue to CPK (ECPK) |
| L85: delete “at least” | Done |
| L103: “to admit” | “admitting” replaced by “admit” |
| L183-186. A real-time PCR assay was used to obtain relative quantitation of virus and host genome copy numbers. The virus target is present at one copy per virus genome, and the host target is present at two copies per diploid genome. The text should be modified to make this clear, as well as its influence on data interpretation. | Text adapted accordingly |
| L315. Is “at least” needed here? | “at least” deleted |
| L403. I do not know the meaning of “27’488 and 4679 NNNs”. | Text adjusted |
| L405. A denominator is needed for the 56 nt mismatch. | Text adjusted |
| (comments L417-LK420) | Indeed, we have reorganized and rewritten the entire section (original L408 to 443) |
| L417. Not sure what is meant by “non-core” likely tegument protein | Deleted in the course of the section’s reorganization |
| L420. Roseoloviruses also encode OBPs | “unique” replaced by “unusual”. We are not trying to point out all resemblances or differences here – just to HCMV |
| The L417 and L420 items end up being mentioned in lines 431-434, but those areas might be able to be more tightly integrated. | Some rewriting has been carried out here and 25 lines from this section have been moved to DNA Annotations section of M&M (as request by Reviewer #1) |
| L448. Is the hypervariability within isolates or strains, or between isolates or strains? | No, it is between strains not within strains. "between them" has been added after “genetic divergence and associated with”. |
| The complex string spanning **lines 509-513** contains what seem to be inconsistencies in usage of commas, semicolons, periods, and  parentheses | This whole section was removed. |
| L528. p. 22. Not sure what is meant by “triplex patterns”, which are mentioned here and on p. 22 line 528. | Triplex refers to the inserted alternative three gene cassettes. Within the R2 segment. Re-phrased as “recognize 13 different three gene cassette (triplex) patterns ...and on L583 as ..typical group-D E59/60/61 triplet cassettes.... |
| L642. “routing” to “rooting” | Done |
| L670. “sequence of Kimba contained” | Done |
| L672. “address their biological function in the future | Corrected |
| L675. delete “considered” | Done |
| L676-677. “enzyme, thereby terminating its activity, or” | Corrected |
| L682. I don’t understand the sentence that begins with “Interestingly” | Clarified as follows: Interestingly, Leu792 in our sequence compared to Isoleucine at this position in all ten previously published isolates (L792I). This seeming mutation mapped to the center of a conserved domain, …. |
| L695. delete “single one” | Done |
| Having defined its abbreviation in line 702, use “gB” thereafter. | Done |
| L713. “of gB subtype 1B” | Corrected |
| L728. “revealed much” to “detected” | Done |
| L762. Is “apparently” needed? | “apparently” deleted |
| L778. “homology” to “similarity” | Done |
| L786. “new” to “previously undescribed” | Done |
| L792. “convey information about” | Corrected |
| L793. “If all” to “Since” | Done |
| L802. “Despite the long” | “of” deleted |
| Lines 805, 807, and 809 say the same thing three different ways, which is confusing. “U38 DNA polymerase”, “U38 protein”, and U38  DNA pol. Pick a winner. | Done as follows: … the U38 DNA polymerase may be naturally incompetent to incorporate certain phosphorylated nucleoside analogues into the growing viral DNA. Due to the lack of suitable cell cultures, this issue has not yet been addressed. Still, an interesting variation was observed within the U38 DNA polymerase sequence, where a single mutation (L729I) in all our isolates opposed … |
| 1. It seems like Umesh’s sequence should be included in Table 2. | Done |
| 2. In Fig. 1, the orange used for Thai, is too similar to Indi’s yellow for the parentage of Umesh to be unambiguous. A similar situation exists for Ruwani. It would be helpful to indicate which animals died from EHD. Are the two prior cases those of Xian and Aishu? If so, please say so. | Figure revised accordingly |
| 3. Parts of the case descriptions are difficult to follow because of the several nomenclatures used. Lines 382-390 provide several illustrations of this:  • In line 382, “EP07” is used as a name for an animal. In line 389, “EP55” is described as the name of an isolate. In Table 2, “EP55” is described as a “Case”. From context, I wondered whether similar designations connected to Genbank entries. Too much work is required to decipher the information. It would be helpful to adopt a consistent scheme for such things.  • It would also be helpful to include a comprehensive expanded version of Table 2 that includes all of the animals mentioned in the paper, their birth and death years, virus and strain information, sequence accessions, lengths, and genes (for short sequences), etc. | We agree. Using the names of the animals is helpful to understand, where and how the animals were bred and kept and how various cases interrelate. Importantly, most of the complete EEHV genomes available in public databases are also named after animals. Moreover, much of these “personal” data are publicly available on EleWiki. Therefore, we decided to use predominantly elephant names in our paper, but we use Table 2 to clarify alternative naming such as case numbers and GenBank entries.  Background info and acc nos for the ten genomes used in Supplemental Table S1 are also now similarly listed in the new supplementary Table S2 |
| 4. A single, large “EEHV1 Loads” could be added parallel to the y-axes on the left side of Fig. 3 | We prefer using the term “∆Ct”, which is what we measured. |
| 5. Fig. 4 would be easier to understand if it included the names of the animals, in place of, or at least in addition to codes such as  “E361A_EP07”. | Done |
| 6. Fig. 5 would be improved by addition of animal names and where they are from (American, Zurich, or European). | Animal names included. Origin explained in the caption |
| 7. Fig. 6 would be improved by a lighter color scheme in the upper right half and a larger font in the lower left half. The color scheme for the aligned genome fraction provides little information due to the high degree of similarity across the collection. A color gradient that ranges from 80% or 90% to 100% might be more informative. | Done |
| 8. The red text in Figure 7 is illegible. A larger font will increase the vertical dimension of the figure, but that is OK. | The text is readable (or can be enlarged to the effect) in the 300 dpi version of the Figure |

| **Reviewer #1** | **Our Response** |
| --- | --- |
|  | Line references correspond to the reviewer’s pdf file |
| The results section does not clearly match this title | Part of the wording of the manuscript title has been changed from “Virological Analysis” to “Viral Genomic Analysis” to cover the extraordinarily large amount of overall protein divergence that is now being recognized here across the full length of intact genomes of multiple EEHV1A strains and EHD cases worldwide. |
| In **the** summer **of** 2022 | Done (both title page and Abstract on page 9) |
| “*Elephas maximus*” in Italics | The term is actually in Italics in the Abstract of our word document and pdf version (page 9), but Italics were lost upon transfer of the Abstract to the title page and assembly of pdf files by PLOS One. We shall keep an eye on that issue during re-submission and publication. Thank you. |
| “The conclusions from these analyses may be summarized in three points” replace by “The results show” | Done (both title page and Abstract on page 9) |
| “In particular, ways need to be found to circumvent the present unavailability of appropriate cell cultures and animal models.” (Title page) This sentence does not reflect the topic researched and should therefore be deleted. (Page 9, lines 34-35) This is a stand alone opinion that is not in the discussion or in the research topic, so suggest to delete | Done (both on title page and Abstract on page 9) |
| Page 2 of the assembled pdf “donation from the late Prof. Dr. Robert Wyler” | Done. In addition, “Finanical disclosure” was revised to include financial support for the Johns Hopkins University Investigators |
| Line 35. “circumvent” this is a strange word choice. | Entire sentence deleted |
| Line 44. (EEHV1B), | Semicolon added. Thank you. |
| Line 45. “serologic**al** level” | Corrected as suggested |
| Line 50. Include reference | The case had been presented at the EEHV workshop 2022. Unfortunately, the corresponding author did not reply to our several requests to give us permission to cite their case. Therefore, we decided to modify the text, citing a more general paper. |
| Line 51. Use either African elephants or *L. africana*, but not sometimes one and sometimes the other. The Latin name is only required once at the start of the paper, straight after the common name. | Corrected. Thank you. |
| Line 52. (same comment as above) | Same correction. Thank you. |
| Line 56. “no other susceptible species have been identified” | Done. Thank you. |
| Line 57. Try to avoid emotive words throughout the paper, such as 'suprisingly'.  A better sentence:  Not until recently was the full EEHV genomic sequence identified | Point taken. Sentence adjusted in our own words. |
| Line 60-62. Delete “for poorly understood reasons,” from “All known EEHV strains replicate primarily in the respiratory tract but, for poorly understood reasons, can cause viremia, which is the major precondition for developing EHD.” Instead complete the sentence with “...EHD, and the pathogenesis is poorly understood.” | We disagree. The suggested wording changes the message of our argument. Replication in the respiratory tract serves the purpose of virus excretion and transmission. In contrast, the viremic phase gives no obvious advantage to the virus’s life cycle. Therefore, we worded “for poorly understood reasons”. We re-worded our text to make it easier to understand. |
| Line 63. Delete “is associated to” | Done |
| Line 64. ....antibodies. During this period, subclinical infections..... | Agreed. Thank you. |
| Line 65. ...place, which induces active..... | Agreed. Thank you. |
| Line 66. ...later in life. | Agreed. Thank you. |
| Line 67. ...EDH during and right after weaning (2-8y), in association with the waning of maternal antibodies. | Agreed. Thank you. |
| Line 70. Replace “quite” by “usually” | “Quite” deleted without replacement |
| Line 72. In the same zoo? | Yes |
| Line 73. Insert reference | Unfortunately, this story has not been published. To compensate, we inserted individual GenBank references. |
| Line 73. Delete “However” | Done |
| Line 85. Delete “at least” | Done |
| Line 92. Delete “including ours” | Done |
| Line 93. ....strains, and to .... | Done |
| Line 103 ff. Please describe the entire herd and husbandry without emotive words and anonimise the names for ease of reading.  E.g. A herd is made up of two males and seven females (2.7), then you can give the ages groups etc. the cases you describe should be Case 1A, 1B, 1C, for example. You should give details on these in the text, e.g. age at the time of the incident you are describing.  The rest of the herd can be described impersonal as well with details in an appendix: e.g. 'breeding bull'. If its important that he had newly arrived, this can be mentioned in the introduction but this fact does not need to be mentioned in the discussion again.  If the family group is clinically important, call them Group I and Group II or similar, with their origin, but steer clear of names.  If the actual names of the animals are important for international discussions, put those in an appendix. | **We disagree** for the following reasons:  Most of the complete EEHV genomes available are named after animals.  Due to the website EleWiki the “personal” details of the elephants, including their cause of death, are publicly available (<https://www.google.com/url?sa=t&source=web&rct=j&opi=89978449&url=https://elefanten.fandom.com/wiki/Hauptseite&ved=2ahUKEwjOvI-orO-HAxWUgv0HHSY6AOYQFnoECB0QAQ&usg=AOvVaw2Ss-w-RLV5SEX_JQsiDlGY>)  Naming the animals is also helpful to track previous as well as further cases, to appreciate how the animals were kept and bread, and to find information about the correlation between animals.  As stated by reviewer 2, we have already a complicated situation due to case numbers (for example EP55) and GenBank entry numbers, which refer to the same animal but neither of them is available for unaffected animals. Using just animal names throughout the text will make reading much easier. Anyway, EleWiki makes names and backgrounds publicly available. |
| Lines 103-104. This is called a 'closed collection'. Rephrase to use these proper phrases - it makes the text more succinct and clear | Rephrased |
| Line 104. Report actual age | Done |
| Line 104. Replace “bull” with “male” | Done |
| Line 104. A new breeding bull was **introduced** to the herd.... | … a 10-year-old male was introduced to the herd … |
| Lines 105-106. Delete – irrelevant | We disagree, these were the exact reasons to acquire a new breeder from outside |
| Line 106. There were xxx juveniles at risk in the herd (ages) | In 2014, it was exactly the one animal. Yet, we rephrased the text to note the advent of two additional calves (2017 and 2020, respectively) and their integration into preventive surveillance. |
| Lines 106-107. Delete “it seemed appropriate” | Done |
| Lines 107 ff. To protect at-risk herd members, a EEHV preventative protocol was instigated, which included ..... | Rephrased |
| Line 110. Replace “Thai” with “new breeding bull” | We disagree with the arguments discussed above. |
| Lines 111-112. by EEHV 1 and EEHV 4 PCR | Done |
| Lines 112-113. “Serology was established “en route” as it became available from literature”  delete this sentence - it does not mean much.  State clearly when serological samples were collected from | We disagree. Our screening program evolved over time, in particular as it concerns serology. Reports about valuable ELISAs and LIPS assays and their proper interpretation became only available between 2020 and 2022 (as we cite properly). |
| Lines 113-114. Replace present text with “A EEHV reactive protocol was additionally in place, which included stocking of gancyclovir and famcyclovir for treatment of clinical cases.” | Rephrased |
| Lines 115-120. Replace present text with “Eight years later, three juveniles elephants from this herd died of EHV caused by a previously undeteced EEHV1A strain. This paper describes the attempts at identification of the shoudre of this outbreak through serological surveyance.” | Rephrased as suggested |
| Line 115. Please review your entire paper and remove all emotive expressions | Done |
| Lines 122 ff (Materials and Methods):  1) describe herd and give all cases a number for ease of reading. Do not use animal names throughout the paper. Include details on the described cases in this paper (3 cases), put all other details in an appendix or as supplemental material | The reviewer requested already in Line 103 to refrain from using animal names. Here, even the introduction of a case number is requested.  However, Reviewer 2 criticizes in his issue #3 that “*Parts of the case descriptions are difficult to follow because of the several nomenclatures used*.” Indeed, each elephant has its own name but only animals that succumbed to EHD have a case number, for example EP55 in the case of the present case Umesh. Even cases of which no GenBank entries exist, may have been provided with a case number, for example EP16 Aishu. Importantly, Genbank entries of full EEHV genomes provide most often elephant names, for example the American prototype EEHV1A “Kimba” and the European prototypes EEHV1A “Raman” and EEHV1B “Emelia”.  As “personal” information about many elephants (including all individuals from our zoo), is publicly available on Elewiki (for example <https://elefanten.fandom.com/wiki/Umesh>), the introduction of anonymizing case numbers for the sake of this paper would only complicate matters. We feel that this type of transparent information is required to gain and spread scientific information about EHD. Consequently, we suggest to use the elephant’s names consistently throughout this paper. |
| 2) clearly describe the husbandry setting in this section, including access, frequency of testing, methods of sampling etc | Description of husbandry setting included and referenced. Sampling frequency clarified. |
| 3) put all methods in this section, including thought process on why certain methods were used. Remove method descriptions from the results section | We agree to a certain extent, having adjusted M+M accordingly. However, the purpose of the experiment is at times very useful, just prior to describing the observation in the Results section. |
| Line 144. If the family tree is important, please explain so in the discussion | Arguments used in the revised discussion. |
| Lines 153-154. Were these used as controls?  Your title implies that you are only looking at samples from Switzerland, but you have also analysed samples from German cases. This is confusing. Please consider how these samples influenced your thought process and include rationale on why these samples were analysed for this study | Yes, these were made available to us as positive controls (positive in terms of: this sample comes from an elephant, who succumbed to EHD). Text adjusted accordingly. |
| Line 159. Serum samples from four individual African elephants from a separate Swiss zoo were collected that same year. | As the sera were collected throughout January, why not state the fact? |
| Line 162. acknowledgements, not main text body | Agree |
| Line 164. “*and minced in a combination of 300 μL of body fluid and 300 μL blood*” replace “in a combination of” by “with” | Done |
| Line 167. acknowledgements, not main text body | Done |
| Line 171. TNF-alpha? | Yes, added to the revised manuscript. Thank you |
| Line 178. reference for cut off value | The decision was not made on previously published results, but it is an arbitrary but important factor for interpreting our data, in particular since we used diluted blood droplets as starting material. |
| Line 190. The first day of viraemia of your first case (index case) is Day 0.  From Day 0, time all the other cases. descriptors such as 'very early' are for the discussion | Probably, we did not state it clearly enough, but it was indeed our intention to sub characterize the causative virus as early as possible because this knowledge had the potential to influence our therapeutic options. For example, if the virus had been identified as subtype 1B, we might have had the option to use Omysha’s serum (the elephant who had been identified as shedding subtype 1B prior to the outbreak) for treating Umesh. Similarly, if the virus had been closely related to the previous Xian isolate, we might have had the option to use the serum of one of the animals that had been there at the time.  While the reasons behind the decision are better placed in the discussion, the fact “early” clearly belongs here into the M+M section. |
| Line 191. PCR technique was .... | We prefer “*parameters*” because more than bare technical aspects are affected. (“*conditions*” replaced accordingly) |
| Line 207. Please include published reference | Done |
| Line 208. (GenScripts Europe, the Netherlands). | Replaced according to your suggestion |
| Line 224. add address of manufacturer | Done |
| Line 234. These were kept in .... | Replaced |
| Line 237. With -> by | Replaced |
| Line 240. manufacturers address | Inserted |
| Line 241. 8 -> eight | Done |
| Line 249. Insert reference | Done, including explanation |
| Line 252. Delete “*Eventually*” | Done |
| Line 256. What was sheared? The noun is missing from this sentence | The nucleic acids (inserted) |
| Line 265. italics | Done |
| Line 267. Subsequently, | Done |
| Line 270. Italics | Done |
| Line 270. NCBI: Write out in full the first time used in text | Done |
| Line 289. The results section is too elaborate and includes text that belongs in materials and methods and some which belongs in the discussion.  ONLY list the actual results from your study, in a clear and concise way.  Interpretation is for the discussion section. Some parts can be moved to supplemental materials. | Done. The results section largely remains intact, although rewritten and rearranged extensively throughout to maintain an integrated and cohesive section on strain and gene subtyping. However, some text has been moved out to M&M entitled Annotation of EEHV Genomes. In addition, a new section of text covering background and history of EEHV subtyping for the Introduction and another as a summary and comments on EEHV subtyping for the Discussion. |
| Line 291. Between x date and y date, .... | Done |
| Lines 293-294. 'tolerated sampling procedure'  Too emotive  In materials and methods, it should be described that sampling, based on animal access and training, commenced on Day x for case 1B and Day y for case 1C. | The paragraph has been adjusted. |
| Line 295. 1)How long were the elephants viraemic for?  2)How high was the viraemia? Was a qPCR done on positive samples?  3)What was the time between viraemic events?  4)Did treatment influence viraemia? | Ad 1) not repeatedly, until outbreak.  Ad 2) the screening was done by Taqman PCR; ΔCt values were used to estimate the viral load (as described in M+M). The paragraph was re-written to clarify.  Ad 3) Very irregular. Details will be seen on our data sheet.  Ad 4) no effects at all observed. |
| Line 306. Rationale for using horse serum should be in materials and methods | Done |
| Line 307/308. If these sera were part of the study, and not published elsewhere, these cases, with different case numbers, should be included in that table. | Unfortunately, specific data about the EHD cases of Kanja and Anjuli have not been published. But blood samples have been made available to us to serve as positive controls (as described in M+M). These sera were, therefore, not part of our study. |
| Lines 312-313. Discussion sentence | We disagree. We describe an observation. |
| Lines 313-321. Discussion - this is interpretation - interpreting one result against another. The actual results are not clear from this section.  Please list only the results first, of all the samples tested for this study, and include a table of ALL the results. Interpretation can come afterwards, separately | We disagree. The results of all serological assays are combined in Fig. 2. In the text, we point out the reactions of individuals in a descriptive manner (as appropriate for the Results section). |
| Lines 322-324. Materials and methods | Done. See section “LIPS assay” |
| Lines 325-326. Is this a result of your tests? If so, include it in the listing of all results plus include in the table, to give the reader an overview. | Yes, the results are presented together with all other serological results in Fig. 2. |
| Lines 327-328. Discussion | We disagree. It’s summing up all of the serological data in one sentence. |
| Line 345. This date is irrelevant, the weekday especially.  If Day 0 was the first day of viraemia of your index case, which day was this? Day -7? | The weekday may be irrelevant, although screenings were usually done on Thursdays. However, on this day of routine screening, all of the elephants were still aviremic, while exactly one week later, the outbreak was first detected. As the screening took place on a weekly schedule, we believe that this information is important. |
| Line 346. this is Day 0, presumable.  A clear timeline is very important for your reader to interpret and critically assess your findings | As explained above, we do not really know Day 0 or Day 1. |
| Line 351. Day 3 | See above. |
| Line 352. Day x | See above. |
| Line 354. Day y | See above. |
| Line 355. delete  This word does not belong in the results section | We disagree. |
| Line 357. Case 1A, 1B and 1C | Adjusted, individual names used |
| Lines 365-369. Materials and methods  In the results section, only state:  Conventional PCR targeting the E36/U79 locus showed .... | Adjusted |
| Lines 378-380. Materials and methods | Adjusted |
| Lines 383-384. materials and methods  In the results section, only state:  Comparison of Case 1A with X and Y showed ..... | Adjusted (names) |
| Line 406. Rationale for this should be in M&M, not in results | We disagree. This decision was not planned but was a direct consequence of the results described in this section. |
| Lines 412-427. Abridge and move to discussion | We disagree. The description of the genomic organization is an important major lead-in here to the whole large results section describing multiple separate findings about levels and patterns of divergence found within numerous hypervariable regions within both Umesh itself and many diverged reference genomes. |
| Lines 428-430. (Highlighted but no written comment) | ?? (probably part of the previous and next comment?) |
| Lines 431-443. Abridge and move to materials and methods | This section has been moved to M&M as suggested under the new subtitle of Annotation of EEHV Genomes. |
| Lines 446-452. Introduction? | Whilst it could be used in that way, there is already a new section in the Introduction covering earlier work on some aspects of strain variability in the Introduction, whereas, this section remains as a necessary lead-in to the major new results section immediately following this about detailed qualitative and sometimes quantitative differences from other complete EEHV genomes both globally (Fig 6) as well as at key variable gene loci. |
| Lines 465-466. M&M | Thank you, see new Chapter "Annotation of EEHV genomes" in M+M |
| Line 468. Delete „*it is noticeable that*” | Changed to “Furthermore, “ |
| Lines 470-476. interpretations  Abridge please, the results section is not clear | Lots of rewriting here, with parts also moved to Introduction. |
| Lines 477-481. M&M | Now omitted |
| Lines 488-498. relevance to title of the paper is not clear. Supplmental materials? | The previous detailed comparisons to EEHV1B have been largely omitted, with some of the rest moved to M&M. The title of the paper has been updated to include “Viral Genomics” and fully integrate the gene subtyping results with the rest of the text as a major part of the manuscript |
| Line 498. Delete “*finally*” | Entire section deleted |
| Lines 505-513. Delete 6 highlighted expressions | Done in the course of other changes in the text |
| Line 513. Replace “*of this information for*”by “*of Case 1A*” | Disagree, name still used (see elsewhere) |
| Lines 516-556. Distill the actual result and provide the full explanation in supplemental materials | Both Table S1 and the new added Table S2 are now given as Supplemental Materials accompanying the text for the overall Gene Subtyping results and comparisons sections presented as a coherent. |
| Lines 559-569. Distill any results and move the explanation to supplemental materials | Same as for 516-575 but considerably rephrased and reorganized as a single cohesive section under the subheadings of Comparison with main segment EEHV1 variable gene subtype arrangements and Comparison with other EEHV1 R2 segment arrangements. |
| Lines 570-575. M&M? | Same as for 516-575 but considerably rephrased and reorganized as a single cohesive section as above |
| Lines 581-598. This section is very verbose and the result, and its relevance to the title of the paper, are not clear | The title of the paper has been updated to include “Viral Genomics” covering the now fully integrated gene subtyping results within the main results text as a major part of the manuscript |
| Lines 604-610. supplemental materials | We disagree. This background info is best kept together with the rest of the clarifying info re overall Umesh R2 segment subgrouping results for different strains given in Supplementary Table S1. |
| Lines 611-615. Discussion | Moved |
| Lines 615-620. Not a result - move elsewhere | Moved to end of Annotation section in M&M. |
| Lines 620-621. Discussion | We disagree. It would be weird to isolate this brief comment from its overall context just above. |
| Lines 624-626. Repetition | Serves as a necessary linker statement between the end of the extensive Umesh versus other strain difference comparison results above and the switch to focus on targeted questions about potential pharmacological inhibitor issues. |
| Lines 633-634. Introduction | Again, serves as a lead-in to a new subsection. |
| Lines 635-636. Delete | Serves as part of a brief preamble to the new enzymatic assessment section. |
| Lines 643-644. Delete | No, retained as a relevant follow-up comment that would be lost if moved to another section¨ |
| Lines 644-646. This can be the first line of your discussion | We do not want to separate this from being a summary of the previous extensive results in front of it. |
| Lines 654-656. Materials & methods | Retained = preamble to results |
| Line 656. Delete “*Indeed,* ” | Done |
| Line 657. Replace “*displaying just*” by “,.... *with only one*....” | Done |
| Lines 660-662. Discussion | No, retained as a relevant adjunct comment on the result |
| Lines 665-666. Delete several words | Done |
| Lines 671-672. Discussion | Difference from Umesh = Part of the results |
| Lines 678-679. M&M | It’s also the rationale and preamble for the present experiment |
| Line 679. Delete “*Notably*,” | Done |
| Lines 685-688. relevance unclear | The entire section has be re-organized for clarification |
| Line 703. Elephants. | Done |
| Line 704-706. delete  instead: advantage, as it is not species specific. | Done |
| Line 706. Delete  instead: Therefore, horse sera could be used to determine the negative cut off value. | Done |
| Line 708. Delete (and replace with):  All adult elephants had ... | Done |
| Line 709. Delete “*sera from*” | No, it is needed to make sense |
| Line 709. Acknowledgements | Deleted from Discussion |
| Line 710. Delete “*also*” | Done |
| Line 715. Delete “*the* *sera from*” | Done |
| Line 715. (Insert) “...., possibly due to subclinical infection with EEHV6” | Argument rephrased |
| Line 716. were the samples tested for this? If not, please provide a reference why this assumption was made. | No testing for EEHV6 was done (serology assay not available yet). Almost all adult African elephants have EEHV6 (plus EEHV2 and EEHV3) DNA in saliva, but EEHV6 is the most likely to cross-react with EEHV1 reagents. Text rephrased accordingly. |
| Line 721. delete.4  ....may be explained by .... | Sentence modified accordingly |
| Lines 725-727. Delete | Rephrased but retained |
| Line 733. keep names out of the discussion - not clear | Disagree as explained above. |
| Line 738. short-lived, and therefore young elephants are at risk during an EEHV outbreak. | Rewritten to clarify our opinion |
| Line 739. reference increased shedding here? You are only discussing viraemia - viraemia does not equate shedding - shedding puts others at risk  also, treatment of a sick elephant can cause stress of the other juveniles due to routine disruption - that can set off latent/low level viraemia | We agree but viremia in a very young elephant does not occur without prior transmission, due to shedding from an adult. Both shedding and viremia may proceed unnoticed. A more suitable reference was inserted.  True, but according to the screening results, apparent stress episodes (for example killing of a newborn by members of the herd, which occurred in Winter 2021) had not evoked any viremic phases. Moreover, EHD involves primary infection not reactivation |
| Line 741. This should be included or published in tandem - treatment may influence viraemia. If this information cannot be published with this paper, this paper should focus only on identifying the source, and not speculate on onset of EHD, viraemic levels throughout EHD, etc. It is important to know what veterinary interventions were done so interpret the viraemic levels seen. | We agree to a certain extent. However, the zoo declined to publish precise data about the treatment. We know that Umesh was not treated with antivirals prior to June 24^th^, whereas Omysha was not treated prior to July 5^th^. Ruwani refused at all to being treated. Obviously, no measurable effect of the drug treatments can be seen.  In contrast, measuring the viremic levels was part of our own investigations. Neither these data nor their interpretation should be withheld. The text was modified accordingly. |
| Line 743. emotive  use instead: died on Day x | “succumbed” changed to “died of |
| Line 743. qPCR results would be useful here. CBC results as well. If none of these can be incorportaed in thsi paper, this paragraph may need to be deleted as speculations are made without proper data to support it. | These results are actually shown in Fig. 3. We inserted the corresponding cross reference into the text. |
| Lines 756-759. Delete | We disagree. This is an important aspect of the full story here. |
| Line 761. delete  instead:  ...ruling out a re-emergence of a previously latent strain | Done |
| Lines 762-763. delete, repetition | We disagree. It is important to state the fact in the context. |
| Line 782. the breeding bull | Done |
| Line 783. Case 1B? | As explained above, we have decided to use animal’s names, whose background information is presented in Fig. 1 |
| Line 790. Reference | 4 references inserted (16, 40-42) |
| Line 796. Follow on from here with line 814 | Disagree. We believe that it makes sense to keep the issues of potential drug resistance apart from the potential role in epidemiology |
| Line 802. Delete highlighted word | Done |
| Lines 804-805. this is necessary to interpret the results. Refrain from all speculation with regards to effects of treatment on viraemia if this data is not available to you  consider a tandem publication so that the case becomes clearer as an alternative, or publish as a single paper of more scientific power | Disagree. It is a general and very important issue that needs to be addressed.  We did consider it. To no avail. |
| Line 814. connect ot 796 | Disagree (as argued in our response to Line 796 comment) |
| Line 819. M&M | Disagree. This information is important in the context. With the exception of three elephants (Ceyla, Indi, Thai), all members of the herd had been born in our zoo. Therefore, only Ceyla, Indi, and Thai can bring a “new virus” into circulation. Therefore, it is important to discuss, where they came from and when. |
| Line 824. Any idea what (husbandry) event could have triggered shedding of a latent infection? | Indi, at the age of 38, is getting old. However, reactivation events are always asymptomatic. To our best knowledge, the true triggering factors have not yet been identified. |
| Line 829. (Replace by) “*Possibly*“ | Done |
| Line 830. by deduction - or do you have firmer evidence? Has this elephant ever shed this strain? | As stated above, Indi and her daughter Chandra were the only members in the herd to have antibodies against ORF-Q-C and, thus, to have encountered the virus prior to the outbreak. Chandra was born in the zoo, while Indi originates from Myanmar, leaving Indi as the most likely original source of the virus. As stated above, occasional asymptomatic periods of shedding go unnoticed. |
| Line 831. This may be a conclusion if the treatment protocol was incorporated in the paper, which it isn't | Disagree. This general knowledge is reconfirmed by the present case. It is also a very significant point that the reader needs to be reminded about |
| Line 832. delete  This is not in the discussion and not in the results | But it is generally known and influenced preventive measures as well treatment options |
| Lines 840 ff. (References section) Please put all latin names in italics | Agree. The format became lost during upload of the manuscript. |
