# Supplementary material for: Epidemiological, serological, and viral genomic analysis of an outbreak of elephant hemorrhagic disease in Switzerland
Source: PLoS One. 2025 Apr 7;20(4):e0301247. doi: 10.1371/journal.pone.0301247 (PMC11975098; doi:10.1371/journal.pone.0301247)
Supplement: Attachment — Submitted filename: 2nd_Rebuttal Letter PONE_submit.docx [file pone.0301247.s007.docx]

Rebuttal Letter PONE-D-24-09462_R1

We thank the reviewers as well as the editor for their careful and considerate work. While reviewer #2 did not raise any further issues but positively commented our revisions as follows: “*The authors are to be commended for the meticulous work done in dealing with the unfortunate clinical situations and in the depth and manner in which they have organized the compiled associated dataset and narrative, including submission of the numerous DNA sequences to Genbank. The original reviewers made numerous suggestions for improving the manuscript. As detailed in their Rebuttal letter, the authors have done a very commendable job of responding to the suggestions*. *I have nothing to add.*”

In contrast, reviewer #1 raised four editorial issues that need to be addressed in the following table. Furthermore, he pointed out spelling and language-related issues, which we globally corrected in the manuscript. Thank you again. In addition, the editor raised four issues, which will also be addressed in this table.

| **Reviewer #1** | **Our Response** |
| --- | --- |
| Introduction is 6 pages A4, please abridge to max 3 pages but referring to appropriate references. The introduction is too comprehensive, and should only cover  1) The global issue of EEHV  2) The difficulties with the genome, in broad lines  3) The presenting case, which leads into:  4) What this study is looking at | We shortened and restructured the Introduction down from 150 lines to just 100 lines with some of the rest being moved to a new Supplementary Information section. |
| Try to remove all animal names from the text. Use Calf A, Calf B, Calf C. Bull 1, Bull 2, etc. You can have a tablet or figure in which these neutral phrases are connected to the animal’s name. By doing so, it will be easier for the reader to critically assess the course of the disease and results of what you’ve found. | We disagree with the same logic and reasoning as before, and note that at many of the now numerous International EEHV Conferences and Annual Workshops a vast majority of the participants always make a plea for maximum transparency for everybody’s benefit. |
| Results – keep these factual by only reporting them, not interpretating them until the Discussion section, excluding the comparisons part of your study. Do not use emotive words, or interpretative words in this section. | Done including removal of all emotive and interpretative terminology. |
| Treatment of the calves is touched on in the discussion but details are not included. Either include the treatment in your case description, or if it is novel and warrants a  separate publication, publish both papers in tandem so that you can refer too it. | We disagree.   - (Q) Is it possible that antiviral treatment has influenced the observed viral sequence? (A) Yes, it is possible that a viral mutant, resistant to the treatment, has emerged. - (Q) Did this possibility alter in any way our sequencing results? (A) No, as the complete sequence was obtained from tissues obtained at necropsy, our data are not affected. - (Q) Does the knowledge about attempted antiviral treatment affect our interpretation of the results? (A) Yes, without treatment, the likelihood of an emerging resistant mutant would be out of question. Therefore, it is important to mention the fact of attempted treatment. Not mentioning the fact would be unethical. - (Q) Does knowledge about treatment details alter the answer to the above question? (A) No, with more detailed knowledge, we have neither more nor less proof for or against the emergence of a resistant mutant. However, to discuss the issue, we need to mention the fact of attempted treatment, at least as far as we have knowledge of it. |
| Lines 77-83 of R1 manuscript: Add reference to “*Viral genomic sequences have been very useful to determine transmission chains among elephants, which eventually lead to interconnected cases of EHD. For example, the PCR DNA sequences were shown to be identical over at least 3000 bp encompassing the E38/POL, U48/gH-TK), U51/vGPCR1 and U71/gM loci between three cases of EHD at the Berlin Zoo that occurred 11 years apart: Plai Kiri (European Proboscivirus case number 14; EP14) in the year 2000, Shaina Pali (EP23) and Ko-Raya (EP25) in 2011 (e.g. GenBank KT705234.1, KT705238.1 and KT705239.1)*.” | Unfortunately, these data were never published in peer-reviewed journals. However, the facts have been stated in the publicly available GenBank entries. Therefore, we refer to these GenBank entries, stating it clearly in the revised manuscript. The facts speak for themselves and this section has been moved to the Discussion. |
| Lines 112-118: In this manuscript, please try to stay away from names. It makes it very unreadable. Describe the case without names, e.g. Bull 1, Cow 4, Calf A, Calf B, Calf C. You can then use a table of figure to identify the name of each elephant. This will make the manuscript more readable and easier for the reader to critically assess the course of events. | We disagree as stated in our previous rebuttal table. Using the names of the animals is helpful to understand, where and how the animals were bred and kept and how various cases interrelate. Importantly, most of the complete EEHV genomes available in public databases are named after animals. Therefore, alternative designations such as “Bull 1” or “Cow 4” will probably not help but complicate the reading process.  However, understanding the issue from the reviewer’s standpoint, we introduce Fig. 1 here, which sets the names of the individuals into perspective and gives an overview of the relationships between the individual elephants as well as of their life spans and origins. |
| Line 123: A non-peer reviewed letter in a journal is not an appropriate reference. | We disagree with reference to the PLOS submission guidelines:  “*Any and all available works can be cited in the reference list. Acceptable sources include:*   - *Published or accepted manuscripts* - *Manuscripts on preprint servers, providing the manuscript has a citable DOI or arXiv URL.* “   Moreover, the mentioned reference does not convey irrational statements or speculations, but gives more details about the screening process and of our way of thinking and decision-making prior to the outbreak. |
| Line 123 “en route” Suggestion: Serological analysis occurred later on in the disease process. | No, the reviewer’s suggestion does not cover the meaning of “en route”. We changed the wording in the sentence to make it clear and precise. |
| Lines 131 ff. The part below this line is too extensive. It should not be a detailed review of the genome thus fur, but abridged to only highlight the importance of comparisons that will take place in this paper. | Shortened |
| Lines 157 ff. Please add reference or, if this are the authors' thoguhts, move to the discussion | Moved to supplementary materials |
| Lines 171 ff. This is a discussion, not an introduction | Moved to supplementary materials |
| Lines 186 to 188. Delete. | We disagree with reference to the PLOS submission guidelines:  “*Manuscripts reporting animal research must state in the Methods section:*   - *The full name of the relevant ethics committee that approved the work, and the associated permit number(s).* - *Where ethical approval is not required, the manuscript should include a clear statement of this and the reason why. Provide any relevant regulations under which the study is exempt from the requirement for approval*. …” |
| Lines 194 and 195: please add the origin of the elephant | Done |
| Line 200: 'the study collection'  Try in a manuscript to steer away from animal names, places and specific dates. The aim is to convey the details of the disease progression in the first place, which can be reported more neutral. Zurich Zoo can be e.g. in the acknowledgements of in a table. | We disagree. Animal movement is very important in the context, in particular with regard to the source of the outbreak. The newest animal from outside had been introduced in 2014 but the outbreak occurred in 2022. Thus, the source must have been within the herd and silent over a very long time. Therefore, specific dates, names, and places are not trivial. |
| Line 367: Do only the North American EEHV cases have numbers, not the European ones? If they do, please use numbers for all. | Yes, there are EP# and some were already included in Table2.  Definitions have now also been addressed in the Supplemental Information text.   Again, the senior authors have agreed publicly to support the strong elephant and EEHV community calls to share all information about cases and maintain as much transparency as possible within this field.  This includes generating legacy research databases listing basic facts about known EEHV cases worldwide (NAP#s, EP#s and IP#s etc) identifying which EEHV species, strains and symptoms were associated with individual lethal and surviving EHD cases. |
| Lines 381 ff: Is it possible to stick to just one nomenclature? | Unfortunately, there is not (yet) a single nomenclature that seems to be acceptable for all. |
| Lines 384 ff: This feels like a discussion of nomenclature, which may not be best placed in M&M but better in the diiscussion | We disagree. This section describes, how exactly we proceeded with annotations to the newly sequenced genome. As others have drawn the genomic map in the opposite orientation, it is also important to explain our strategy to the reader. Thus, this is pure M+M. |
| Lines 446-447: This should be in your introduction or materials and methods. Alternative, have a timeline of events in a Figure. | Thank you, we cross-reference to Figure 1. |
| Line 472: Dates are very confusing to the reader. You can use e.g. first day of the first viraemia Day 0, as the start of the outbreak, and then say, e.g. Calf B became viraemic on Day x of the outbreak, and died two weeks later. That sentence makes it completely clear, whereas dates do not, as the reader then needs to calculate how long it took. | Thank you. We have added a timeframe to the outbreak in Materials and Methods, where we defined the first occurrence of terminal viremia as day 1 of the outbreak. Subsequently, we changed the text in other sections of the manuscript accordingly. |
| Lines 500 to 501: delete as too emotive for a results section “*it was of immediate interest to determine the identity level among the two viruses*” | We disagree because this knowledge might influence your treatment options, for example, passive antibody transfer makes sense if serum can be taken from herd mates with prior immunity to the present virus. |
| Lines 535-541: Is this specific for this study or in general? If the latter, please move to the discussion | We describe the present viral genome. The text was adapted to make it clear. |
| Lines 557-563: Discussion | Shortened to the present observations. |
| Line 592: delete “*but is absent from all EEHV1B strains as well as from EEHV3 and EEHV4*.” this is not a result from this specific study | Yet, it is a distinguishing feature in the description of the present viral genome and, therefore, worth mentioning in the description. |
| Line 698: This is the most important of your results, and should be the last line of your abstract, and the first line of your discussion |  |
| Line 762: Delete “*All other differences had been previously described for other Etk proteins*.” Not a result | Yes and no. Above, we noted a total of two to eight different amino acids between our ETk sequence and ten previously published ETk sequences. One of those differences was “new” (not reported previously), whereas the remaining seven had been observed previously, although not as “difference”. Therefore, it makes sense to mention the fact at this place. We adapted the text to clarify. |
| Lines 767 ff: This is just a repetition of the results. Start the Discussion with your most important result and compare that to previous publications | We disagree. If we want to learn anything from this outbreak, it is important to properly embed the events into their context. |
| Lines 778-783: Move to M&M; suggested re-wording | We disagree. Here, we discuss the use of the LIPS assays in the context. These assays are not commercially available. It makes sense to explain the use of horse sera to determine the cut-off values, in particular since “true-negative” control sera from elephants are not available. |
| Line 790: delete “consequently” | We disagree. We did not just assume that antibodies against gB6 may cross-react with gB1. We did so, as explained in the previous sentence, on the basis of amino acid identity between the two molecules. |
| Lines 802-803: replace “*although three episodes of viremia had previously been recorded*” by “*despite three episodes of viremia*” | We disagree. Three episodes had been recorded but more could have occurred without being noticed. |
| Line 819: Either publish in tandem with this second paper, so that citation is possible, or include the details in this paper | As explained in our Rebuttal Table (answer to Reviewer #2, Line 741 of the original manuscript), the zoo explicitly declined to publish precise data about the treatment. Indeed, we invited the zoo veterinarian to contribute to our initial manuscript by precisely describing the course of antiviral treatments. However, he declined then and remained adamant later. We assume that this decision was motivated because of the “protected contact principles” that are followed in our zoo with elephants. Depending on the voluntary consent of the animals, these principles make it almost impossible to reach a strict course of treatment. In our first revision of the manuscript, we have added as much of the treatment details as we know of. In contrast, we have no knowledge of the treatment details because the zoo has declined to share those with us. |
| Line 826: delete “*even without consenting to any treatment option*” | We disagree. This animal remained untreated because it declined to be treated. It is an important detail because antiviral treatment may lead to the emergence of drug-resistant mutant(s), whereas absence of treatment has no such possible influence on the observed viral sequences. |
| Lines 834-835: replace “*Moreover, the possibility to use her antibody-rich serum for a potential treatment of the other animals at risk was now ruled out*.” by “*Ruling out the possibility antibody-rich serum for a potential treatment of the other animals at risk*.” | The suggested wording does not make sense. |
| Lines 840-841: delete “*with Shaina Pali and Ko-Raya (EP23 and EP25) being identical to Plai Kiri from*” | We disagree. Without naming the cases, the reader will be unable to look up the proper sources. |
| Line 841: delete “*Yet, a*” and connect the two sentences by inserting “*but comparison*” | We disagree. The first sentence refers to a historical case and is independent from the present case, which is addressed in the second sentence. |
| Lines 857-859: repetition | Yes and no. The “repetition” is stated in a different context. Earlier, conventional PCR had shown that this locus differed between Xian and Umesh. Here, we confirm the same result, based on full genome sequencing and extending it to all three genomes. |
| Lines 862-864: suggested deletion and “significance unclear”. | We inserted a sentence and a reference to clarify the issue. |
|  |  |
| **Editor** |  |
| The introduction needs to succinct and not a full literature review on the EEHV genome. | Introduction was considerably shortened and structured |
| Interpretation of the data should be restricted to the discussion | Done |
| The treatment details should be added to this paper, as these are referred to in the discussion | We disagree (see Q&A above) |
| The abstract needs to clearly reflect the main findings | Done |
